# Expanding the Myxochelin Natural Product Family by Nicotinic Acid Containing Congeners

**DOI:** 10.3390/molecules26164929

**Published:** 2021-08-14

**Authors:** Nicolas A. Frank, Márió Széles, Sergi H. Akone, Sari Rasheed, Stephan Hüttel, Simon Frewert, Mostafa M. Hamed, Jennifer Herrmann, Sören M. M. Schuler, Anna K. H. Hirsch, Rolf Müller

**Affiliations:** 1 Helmholtz-Institute for Pharmaceutical Research Saarland (HIPS), Helmholtz Centre for Infection Research (HZI), Saarland University, Campus E8 1, 66123 Saarbrücken, Germany; Nicolas.Frank@helmholtz-hips.de (N.A.F.); Mario.Szeles@helmholtz-hips.de (M.S.); SergiHerve.Akone@helmholtz-hips.de (S.H.A.); Sari.Rasheed@helmholtz-hips.de (S.R.); stephanhuettel@gmx.de (S.H.); Simon.Frewert@gmx.de (S.F.); Mostafa.Hamed@helmholtz-hips.de (M.M.H.); Jennifer.Herrmann@helmholtz-hips.de (J.H.); Anna.Hirsch@helmholtz-hips.de (A.K.H.H.); 2 German Center for Infection Research (DZIF), Partner Site Hannover-Braunschweig, 38124 Braunschweig, Germany; 3 Department of Chemistry, Faculty of Science, University of Douala, Douala P.O. Box 24157, Cameroon; 4 Evotec International GmbH, 37079 Göttingen, Germany; soeren.schuler@evotec.com

**Keywords:** myxobacteria, myxochelin, nicotinic acid, secondary metabolites, natural product discovery, precursor-directed biosynthesis, total synthesis

## Abstract

Myxobacteria represent a viable source of chemically diverse and biologically active secondary metabolites. The myxochelins are a well-studied family of catecholate-type siderophores produced by various myxobacterial strains. Here, we report the discovery, isolation, and structure elucidation of three new myxochelins N1–N3 from the terrestrial myxobacterium *Corallococcus* sp. MCy9049, featuring an unusual nicotinic acid moiety. Precursor-directed biosynthesis (PDB) experiments and total synthesis were performed in order to confirm structures, improve access to pure compounds for bioactivity testing, and to devise a biosynthesis proposal. The combined evaluation of metabolome and genome data covering myxobacteria supports the notion that the new myxochelin congeners reported here are in fact frequent side products of the known myxochelin A biosynthetic pathway in myxobacteria.

## 1. Introduction

Myxobacteria are Gram-negative bacteria with a variety of remarkable characteristics. They are able to glide on surfaces and show coordinated swarming [1]. Unlike many other bacteria, they are able to form macroscopic fruiting bodies and exhibit unique multicellular behavior mediated through chemical communication [2]. Myxobacteria are an excellent source for secondary metabolites with a broad variety of chemical scaffolds as well as biological activities [3], such as the antibacterial cystobactamids [4], the antifungal soraphens [5], the antiplasmodial chlorotonils [6], and the cytotoxic epothilones [7]. In addition, myxobacteria are also known to produce secondary metabolites with other functions such as pigments, signaling molecules, and iron-chelating siderophores [8].

The myxochelins represent a class of catecholate-type siderophores produced by various myxobacterial strains that have been reported in literature for more than 30 years [9]. The corresponding biosynthetic gene cluster (BGC) encodes the enzymes MxcCD assembling 2,3-dihdroxybenzoic acid (DHBA), MxcE activating DHBA by adenylation and loading it to the ArCP domain of MxcF and the non-ribosomal peptide synthetase (NRPS) subunit MxcG condensing DHBA with the two primary amino groups of l-lysine. The NADPH-dependent reductase domain of MxcG releases an aldehyde intermediate from the assembly line by reduction of the thioester, which is further reduced by MxcG to form myxochelin A or transaminated by MxcL to form myxochelin B [10,11]. Although it has been shown that the promiscuity of MxcE allows the activation and loading of other benzoic acid derivatives to the assembly line to form precursor-derived non-natural myxochelin derivatives [12,13,14], the incorporation of heteroaromatic carboxylic acid precursors has so far not been described.

We hereby report the discovery, isolation, and full structure elucidation of myxochelins N1–N3 from crude organic extracts of the myxobacterial producer *Corallococcus* sp. MCy9049, featuring an unusual nicotinic acid moiety. In order to probe the biosynthetic origin of the newly discovered myxochelins and to achieve higher yields in the natural producer strain, precursor-directed biosynthesis (PDB) was carried out utilizing nicotinic acid as well as nicotinamide as substrates. In addition, we utilized a total synthesis entry to provide higher quantities of the compounds, to prove the chemical structure of the isolated natural products, and to facilitate the generation of further derivatives.

## 2. Results

### 2.1. Discovery of Myxochelin N1

**Figure 1:**
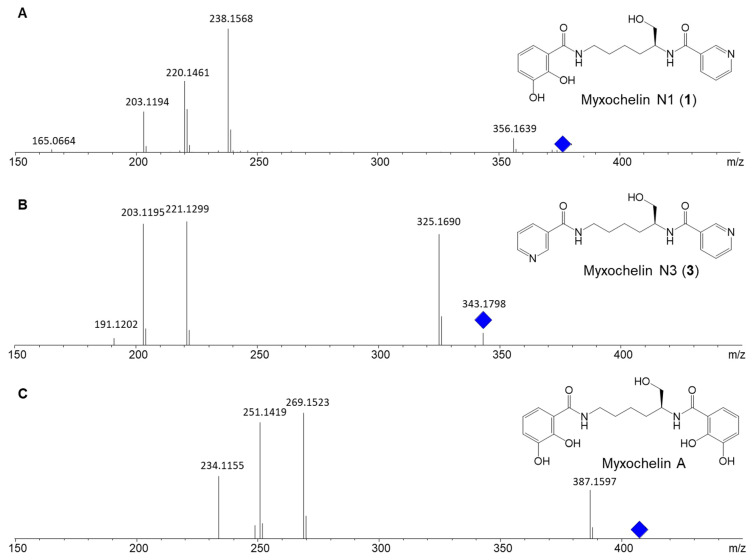
MS^2^ fragmentation spectra of myxochelin N1 (**A**), myxochelin N3 (**B**), and myxochelin A (**C**). The blue diamond represents the *m/z* of the precursor ion.

Analysis of the secondary metabolome of Cystobacterineae species *Corallococcus* sp. MCy9049 [15] revealed the secondary metabolite **1** featuring an ion peak in the liquid chromatography-mass spectrometry (LC-MS) chromatogram at mass-to-charge ratio (*m/z*) 374.1710 [M + H]^+^, supporting the deduced molecular formula C_19_H_24_N_3_O_5_^+^ at a retention time of 3.5 min. The peculiar UV absorption at 219, 249, and 309 nm (Figure S1 in Supplementary Materials) sparked further interest in isolation of the compound. The MS^2^ fragment pattern of **1** was characterized by *m/z* 356.1638 (C_19_H_22_N_3_O_4_^+^), *m/z* 238.1563 (C_12_H_20_N_3_O_2_^+^), *m/z* 220.1457 (C_12_H_18_N_3_O^+^), and *m/z* 203.1189 (C_12_H_15_N_2_O^+^), which according to neutral losses is similar to the known natural product myxochelin A (Figure 1). The quantity of **1** in the crude organic extracts of *Corallococcus* sp. MCy9049, however, was initially insufficient for full structure elucidation and evaluation of biological activities. Therefore, we employed a two-pronged approach to access this alleged new secondary metabolite, using both precursor-directed biosynthesis and total synthesis.

### 2.2. Precursor-Directed Biosynthesis and Isolation of Myxochelin N1 and N3

**Figure 2:**
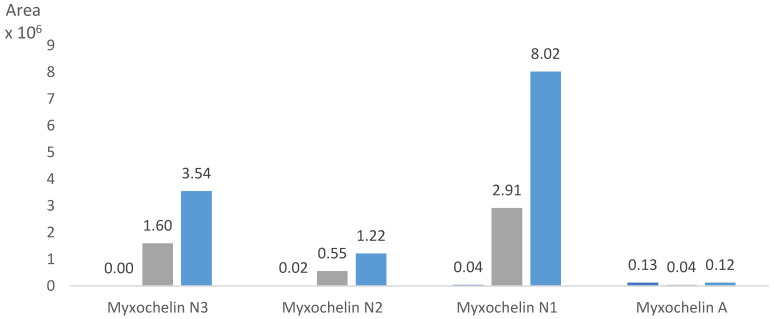
LC-MS chromatogram peak areas of myxochelin N1–N3 and myxochelin A in control (dark blue) and with nicotinic acid (gray) and nicotinamide (light blue) supplementation. Numbers above bars indicate EIC peak area × 10^6^.

In order to access sufficient quantities of **1** for structure elucidation by NMR, a PDB approach was conducted aiming at increasing the yield available from the natural producer. Comparison of the deduced molecular formulae of **1** (C_19_H_23_N_3_O_5_) and myxochelin A (C_20_H_24_N_2_O_7_) as well as their MS^2^ fragments suggested the formal presence of a pyridinecarboxylic acid instead of one of the two DHBA moieties present in myxochelin A. As different hypothetical placements of the carboxyl group give rise to three constitutional isomers that cannot be distinguished from each other with the data available, PDB was conducted by feeding of 2-, 3-, and 4-pyridinecarboxylic acid, respectively, as well as nicotinamide to the culture. While 2- and 4-pyridinecarboxylic acid were only incorporated to a minor degree and showed ion peaks at different retention times (Figure S34), supplementing the producing culture with 3-pyridinecarboxylic acid led to a 70-fold increase in production of compound **1**. Surprisingly, feeding of nicotinamide even resulted in a 193-fold increase of production in comparison to the non-supplemented culture (Figure 2). In addition, the PDB approach revealed the two additional metabolites **2** and **3** with *m/z* 374.1712 (C_19_H_24_N_3_O_5_^+^, retention time 3.2 min) and *m/z* 343.1766 (C_18_H_23_N_4_O_3_^+^, retention time 2.0 min), respectively, possessing similar MS^2^ fragmentations compared to **1** and myxochelin A (Figures S14–S17 and S32–S34).

**Figure 3:**
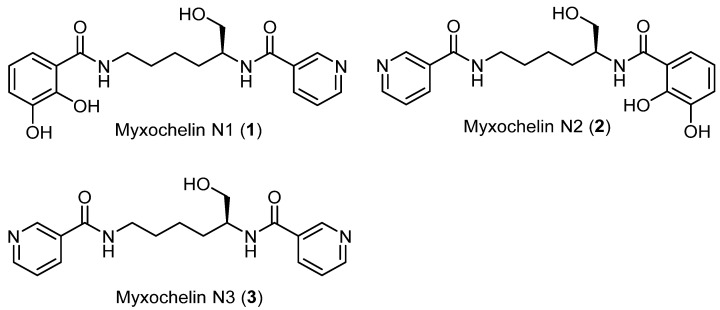
Chemical structure of the natural products **1**–**3.** Structure elucidation of **1** and **3** was performed with the isolated compounds (Section 2.3), structure elucidation of **2** was performed with its synthetic homolog **9b** (Section 2.4 and Section 5.6).

In order to isolate **1**–**3** (Figure 3), *Corallococcus* sp. MCy9049 was cultivated in larger-scale batch fermentation in shake flasks with feeding of nicotinamide. Purification of the secondary metabolites was performed by liquid/liquid partitioning of the crude extract followed by semi-preparative HPLC with UV detection. However, only compounds **1** and **3** were obtained in sufficient amounts allowing their full structure elucidation by NMR.

### 2.3. Structure Elucidation

**Figure 4:**
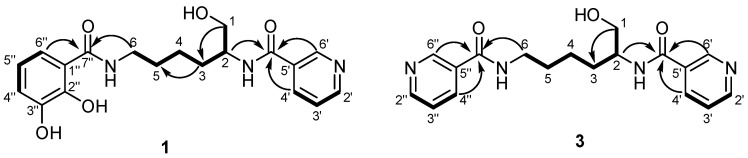
Atom numbering and selected COSY (bold) and HMBC (plain) correlations of **1** and **3**.

Compound **1** was obtained as a brownish oil. Its molecular formula was assigned as C_19_H_23_N_3_O_5_ based on a prominent ion peak at *m/z* 374.1710 ([M + H]^+^, calcd for C_19_H_24_N_3_O_5_^+^, 374.1711) in HRESIMS, which requires ten degrees of unsaturation. Inspection of the ^1^H NMR spectrum (Table 1) displayed two separate aromatic systems. The first consisted of the signals at δ_H_ 7.16 (d, *J* = 8.1 Hz, H-6″), 6.90 (d, *J* = 8.0 Hz, H-4″), and 6.67 (t, *J* = 8.0 Hz, H-5″), which is typical of a 1,2,3-trisubstituted benzene system (Table 1). This observation was confirmed by the HMBC correlations from H-5″ to C-1″ (δ_C_ 115.3) and C-3′ (δ_C_ 145.9), as well as from H-4″ and H-6″ to C-2″ (δ_C_ 148.6) (Figure 4). Based on the HMBC correlations from H-6″ to C-7″ (δ_C_ 170.1), it was proven that this aromatic group was attached to an amide group at C-1″, confirming the presence of a benzamide moiety in **1** (Figure 4). The second spin system consisted of the signals at δ_H_ 8.96 (brd, *J* = 2.2 Hz, H-6′), 8.65 (dd, *J* = 4.8, 1.5 Hz, H-2′), 8.20 (brd, *J* = 7.7 Hz, H-4′), and 7.49 (dd, *J* = 8.1, 5.0 Hz, H-3′) ascribed to a pyridine moiety as underpinned by the HMBC correlations from H-6′ to C-4′ (δ_C_ 135.7), C-2′ (δ_C_ 151.0), as well as from H-3′ to C-5′ (δ_C_ 130.9) (Figure 4). The HMBC correlation from H-6′ and H-4′ to C-7′ (166.7) confirmed the presence of a nicotinic acid moiety in **1**. The remaining signals of three aliphatic methylene groups at δ_H_ 1.49 (m, H_2_-4), 1.63 (m, H_2_-3), 1.72 (m, H_2_-5), an aminomethylene group at δ_H_ 3.39 (t, *J* = 7 Hz, H_2_-6), an oxygenated methylene group at δ_H_ 3.63 (brd, *J* = 3.4 Hz, H_2_-1a) and δ_H_ 3.61 (brd, *J* = 3.8 Hz, H_2_-1b), and an aminomethine proton at δ_H_ 4.15 (m, H_2_-2) were attributed to a 2,6-diaminohexan-1-ol moiety. This assumption was supported by the long spin system, observed in the ^1^H-^1^H COSY spectrum starting from the oxymethylene proton H-1a and H-1b via the aminomethine proton H-2, terminating at the aminomethylene proton H_2_-6 (Figure 4); as well as by the HMBC correlations from H-1a and H-1b to C-3 (δ_C_ 30.2), H_2_-3 to C-1 (δ_C_ 64.4), and C-5 (δ_C_ 28.9). The linkage between the hexanol moiety and the nicotinic acid residue was elucidated based on the HMBC correlations from H-2 to C-7′ (δ_C_ 166.7). Moreover, the 1-hexanol moiety was also found to be attached to the benzamide moiety as an HMBC correlation was observed from H_2_-6 to C-7″ (δ_C_ 170.1). These data were similar to those reported for myxochelin A [16], with the only difference being the replacement of one of the two DHBA moieties by a nicotinic acid moiety, which is consistent with the MS^2^ spectrum of **1** showing the loss of the dihydroxybenzoyl group (Figure 1).

**Figure 5:**
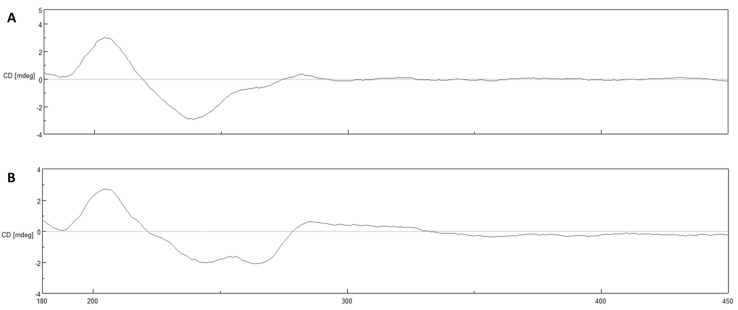
CD spectra of **1** (**A**) and its synthetic analogue **9a** (**B**).

Despite the fact that from a biogenetic point of view, compound **1** should possess the same stereochemistry as the co-occurring myxochelin A, we confirmed the absolute configuration of **1** through its total synthesis (Section 2.4). The total synthesis doubtlessly revealed the absolute configuration of **1** to be *S* as reported for myxochelin A [16]. This finding was further confirmed by comparison of the CD spectra (Figure 5) and optical rotation of **1** and its synthetic analogue **9a**. Thus, compound **1** was confirmed as a new myxochelin derivative featuring an unusual nicotinic acid moiety.

Compound **3** was isolated as colorless oil. Its molecular formula was determined as C_18_H_22_N_4_O_3_ as a prominent protonated ion peak was observed at *m/z* 343.1766 [M + H]^+^ in HRESIMS spectrum bearing ten degrees of unsaturation. The ^1^H NMR spectrum of **3** displayed resonances of two aromatic spin systems consisting of overlapping signals due to protons with a similar chemical environment (Table 2). These signals included resonances at δ_H_ 8.95 (H-6′/6″), 8.66 (H-2′/2″), 8.20 (H-4′/4″), and 7.51 (H-3′/3″) suggesting the presence of two pyridine moieties in **3**. This observation was confirmed by the HMBC correlations from H-6′ to C-4′ (δ_C_ 136.4) and C-2′ (δ_C_ 151.7), as well as H-3′ to C-5′ (δ_C_ 131.2) (Figure 4). Furthermore, the ^1^H NMR also exhibited the signals of three aliphatic methylene groups at δ_H_ 1.49 (m, H_2_-4), 1.64 (m, H_2_-3), 1.74 (m, H_2_-5), an aminomethylene at δ_H_ 3.42 (m, H_2_-6), an oxygenated methylene group at δ_H_ 3.62 (dd, *J* = 5.4, 10.7 Hz, H_2_-1a) and δ_H_ 3.64 (dd, *J* = 5.2, 10.6 Hz, H_2_-1b), and an aminomethine proton at δ_H_ 4.15 (m, H_2_-2) assigned to a 2,6-diaminohexan-1-ol moiety. Comparison of the NMR and UV data of **1** and **3** revealed a high degree of similarity except for the replacement of the DHBA moiety by an additional nicotinic acid moiety in **3**. This assumption was further underpinned by the absence of the signals of the DHBA moiety in the proton spectrum of **3** as well as by the HMBC correlations from H-4′/4″ and H-6/6″ to C-7′/7″ (δ_C_ 167.2) connecting the hexanol moiety on its both sides with the nicotinic acid moieties (Figure 4). Moreover, this assumption is consistent with the MS^2^ spectrum of **3** showing the loss of the nicotinic acid group (Figure 1).

From a biogenetic point of view, compound **3** should also retain the same stereochemistry as that of the co-occurring **1**, which is in congruence with the negative [α]_D_ values measured for both compounds. Thus, compound **3** is a double nicotinic acid derivative of **1**.

### 2.4. Total Synthesis

In parallel to PDB, we sought to establish a total synthesis route for the new myxochelin congeners. This decision was motivated by the finding that none of the published PDB approaches with myxochelin had previously shown incorporation of heteroaromatic carboxylic acids [12] and thus success with the strategy described in Section 2.2 was all but guaranteed beforehand. Indeed, despite our success with **1** and **3**, we were not able to obtain **2** through PDB with yields sufficient for structure elucidation by NMR.

In 2009, Miyanaga et al. reported the total synthesis of several myxochelin analogues starting from either methyl l-lysinate or methyl α*-N*-Boc-l-lysinate in case of target compounds with different aryl moieties on the α- and ε-amino groups. They then used benzotriazol-1-yloxytris(dimethylamino)phosphonium hexafluorophosphate (BOP) to perform an amide coupling first on the ε-position, followed by Boc deprotection and subsequent coupling to the α-position. Finally, to obtain the target compounds, they removed the benzyl protecting groups of the 2,3-bis(benzyloxy)benzamide moiety under reductive conditions [17]. Recently, Schieferdecker et al. reported a fast and efficient synthetic route to synthesize myxochelin A and several derivatives. They used l-lysine ethyl ester hydrochloride as a starting point and subjected it to amide coupling conditions to employ mainly identical aryl groups on the α- and ε-amino groups. Finally, myxochelin A and derivatives were obtained after reduction of the ethyl ester to the corresponding primary hydroxyl group with lithium borohydride and demethylation of the 2,3-dimethoxybenzamide moiety with boron tribromide [18]. Later, the same group also used this strategy to synthesize a large set of myxochelin analogues to explore the structure–activity relationship with 5-lipoxygenase, an enzyme associated with various types of cancer [19].

**Scheme 1:**
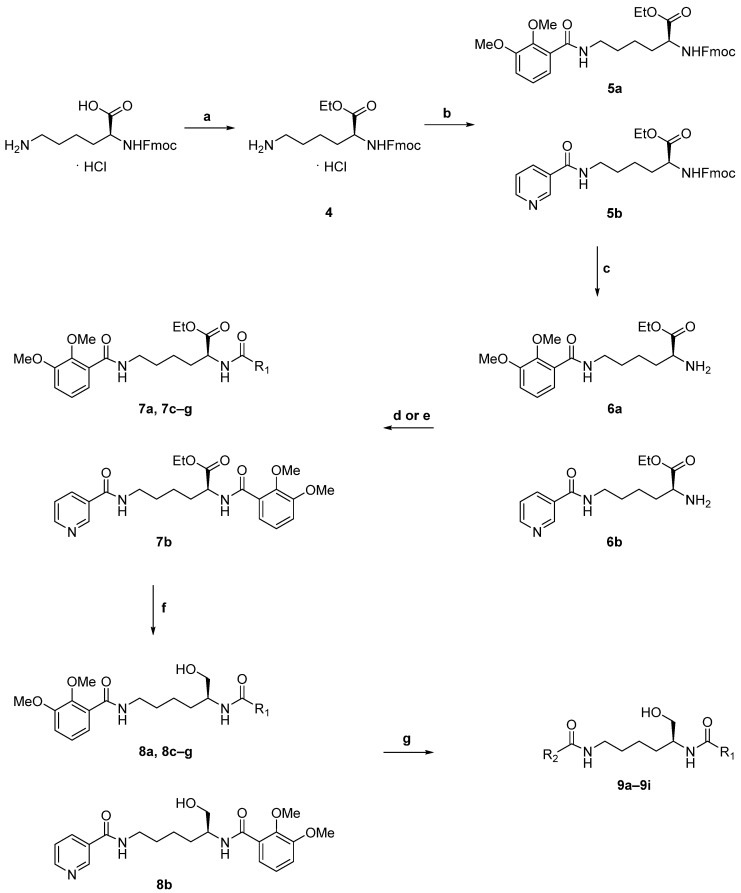
**a**: TMSCl, EtOH, r.t., 16 h, quant.; **b**: HATU, TEA, DMF, 2,3-dimethoxybenzoic acid (for **5a**) or nicotinic acid (for **5b**), r.t., 16 h, 51–83%; **c**: DMA, DCM (1:1), r.t., 2 h; **d**: HATU, TEA, DMF, R^1^COOH, r.t., 16 h, 17–53% over two steps for **7a**–**7d**; **e**: HATU, TEA, DMF, R^2^COOH, r.t., 16 h, then DMA, r.t., 2 h, 13–14% over two steps for **7e**–**7g**; **f**: LiBH_4_, THF, EtOH, 4 °C, 15 min, then r.t., 2 h, 49–71%; **g**: BBr_3_, DCM, r.t., 16 h, 18–27%.

Based on the methodologies reported in the literature, in the present study we synthesized the new myxochelins **1** and **2**. Moreover, we used the established synthesis platform to generate six novel and one literature reported analogues, which are not readily accessible by PDB (Scheme 1). After converting the free carboxylic acid of Fmoc- l-lysine hydrochloride to the corresponding ethyl ester **4 [20]**, the side-chain ε-amino group was subjected to amide coupling using either 2,3-dimethoxybenzoic acid or nicotinic acid. We achieved the fastest conversion with hexafluorophosphate azabenzotriazole tetramethyl uronium (HATU) as activating agent, triethylamine (TEA) as base, and *N*,*N*-dimethylformamide (DMF) as solvent. Afterwards, the α-amino group of **5a** and **5b** was deprotected by removing the fluorenylmethoxycarbonyl (Fmoc) group under basic conditions [21]. By applying the same amide coupling conditions, the second aryl building block was attached providing intermediates **7a**–**g**.In case of aryl substituents with an Fmoc-protected amino group, the Fmoc group was removed one-pot under basic conditions after completion of the amide coupling. The subsequent reduction of the ethyl ester moiety with lithium borohydride yielded the primary hydroxyl group of **8a**–**g** in a pure reaction profile after optimization of the conditions. Lastly, boron tribromide was used to achieve partial or complete demethylation of the 2,3-dimethoxybenzamide system (Table 3). HRMS data of the synthetic products **9a/9b** and the isolated natural products **1**/**2**, and NMR data of **9a** and **1** were identical**.** As aforementioned, to prove the absolute configuration of myxochelin **1**, optical rotation was measured and circular dichroism spectrophotometry was utilized to compare the spectrum with that of synthetic compound **9a** (Figure 5). Finally, the seven target compounds **9a**–**g** of the total synthetic approach were obtained over six steps in sufficient amounts enabling further investigation.

### 2.5. Biological Activities

Myxochelin A has been described to possess weak antimicrobial activity [9] which might be contributed to its iron-chelating properties. Although the new myxochelin derivatives **9a**–**9i** lack the symmetrical 2,3-dihydroxybenzoyl moieties, we found some marginal antibacterial activity in fractions of the extract of the natural producer strain containing myxochelin N1 (**1**) (data not shown). Thus, all synthetic derivatives were tested against a small panel of rather sensitive indicator strains and we also assessed potential synergistic activity of **9a** ( = **1**) with selected reference antibiotics in *Staphylococcus aureus* str. Newman (Table S3). Unfortunately, **9a**–**9i** did not show any activity (minimum inhibitory concentration, MIC > 128 µg/mL) against *Escherichia coli*, *S. aureus*, *Candida albicans*, and *Mucor hiemalis*. Furthermore, **9a** was also not able to potentiate the activity of the reference drugs ciprofloxacin, linezolid, gentamicin, and daptomycin against *S. aureus*.

In 2015, it was found that myxochelins target human 5-lipoxygenase and inhibit the growth of human K-562 leukemic cells at low micromolar concentrations [22]. Here, we assessed the antiproliferative activity of **9a**–**9i** using human HCT-116 colorectal carcinoma and human HepG2 hepatocellular carcinoma cells (Table 4). Intriguingly, derivatives **9c**, **9f**, and **9g** efficiently inhibited the growth of both cell lines at low to mid micromolar concentrations being in line with previously reported activities for myxochelin A [22,23]. Moreover, **9b**, **9d**, and **9e** also showed some cytotoxic activity, while **9a**, **9h**, and **9i** were inactive against both cell lines (IC_50_ > 37 µg/mL). In summary, it can be stated that compounds with a nicotinic acid moiety or fewer free phenolic OH groups appeared to be less cytotoxic.

### 2.6. Biosynthetic Origin of the New Myxochelins ***1**–**3***

The production of myxochelin A by myxobacteria is widespread among the *Cystobacterineae* suborder, but also *Nannocystineae* and *Sorangiineae* species are able to biosynthesize myxochelins (Figure S71). We performed an extended survey covering the occurrence of **1**–**3** across myxobacterial taxa, using a previously established collection of high-resolution LC-MS datasets from ca. 2600 myxobacterial strains [24]. This data evaluation was based on exact mass, isotope pattern, and retention time matching, whereas search parameters were: exact mass deviation below 5 ppm and retention time deviation below 0.3 min. Our findings indicate that similar to myxochelin A, the new congeners **1**–**3** occur predominantly in *Cystobacterineae* species and exclusively co-occur with iron-chelating myxochelin A (Figure S72). Furthermore, supplementing the fermentation media with ferric ethylenediaminetetraacetic acid (EDTA) led to complete abolishment of myxochelin production, including the nicotinic acid congeners (Figure S33), indicating that negative regulation of biosynthetic genes via iron availability took place.

**Figure 6:**
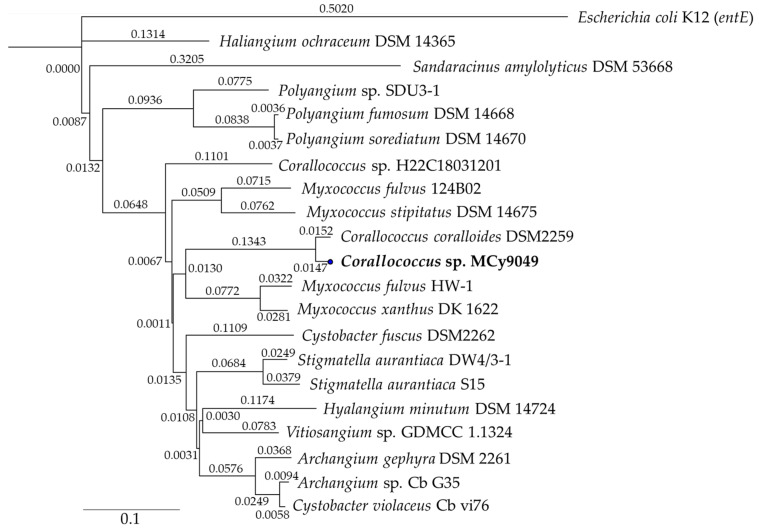
Phylogenetic tree based on translation of *mxcE* genes in myxobacterial genomes using *Escherichia coli* K12 enterobactin synthase *entE* (UniProt P10378) as an outgroup.

Analysis of the genome of MCy9049 further showed one myxochelin-like biosynthetic gene cluster (BGC) that could be responsible for the biosynthesis of **1**–**3**. In order to investigate the loading module MxcE in silico, the sequence of *mxcE* was extracted, translated, and aligned with *mxcE* homologs from published myxobacterial genomes in a phylogenetic tree based on primary amino acid sequence similarity (Figure 6). Additionally, the web server tool NRPSpredictor2 [25] was used to extract the Stachelhaus specificity-conferring codes as well as the 8 Å signature sequences from translated *mxcE* homologs, revealing highly conserved A domain specificity (Table S2).

**Figure 7:**
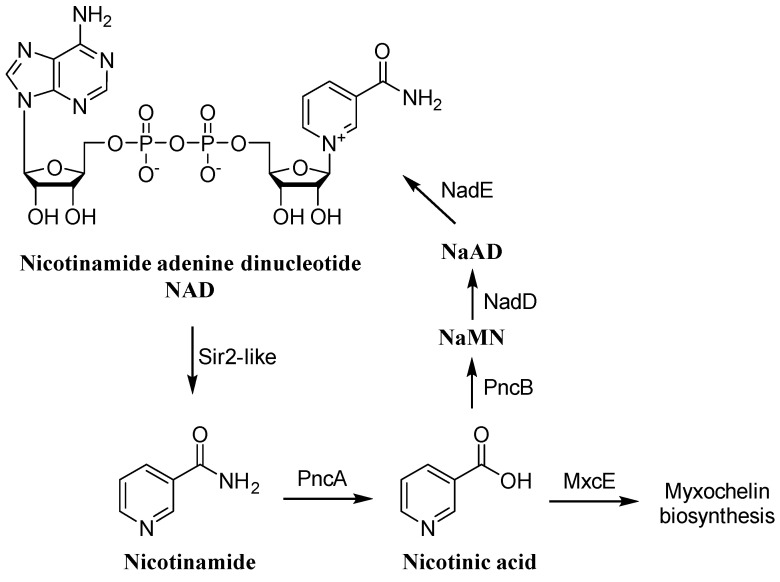
Nicotinamide adenine dinucleotide (NAD) salvage pathway similar to [29,30,31].

We conclude from these findings that **1**–**3** are likely produced via the canonical myxochelin biosynthesis pathway, subject to the availability of nicotinic acid as an alternative precursor. Under standard laboratory growth conditions, nicotinamide as well as nicotinic acid appear in the nicotinamide adenine dinucleotide (NAD) salvage pathway (Figure 7). NAD-consuming enzymes such as silent information regulator 2 (Sir2)-like deacetylases [26] or ADP-ribosyltranferases [27] produce nicotinamide as enzymatic product. Nicotinamide deaminase (PncA) converts nicotinamide to nicotinate, which can then be used for the biosynthesis of **1**–**3**. Besides that, the presence of a *pncA* homolog in the genome of MCy9049 could explain why supplementation of nicotinamide leads to an increase in production of **1**–**3**, albeit NRPS adenylation domains specifically select carboxylic acid moieties for incorporation [28].

Further salvation involves nicotinate phosphoribosyltransferase (PncB) to synthesize nicotinamide mononucleotide (NaMN), and nicotinate mononucleotide adenylyltransferase (NadD) and NAD synthetase (NadE) to re-synthesize NAD via nicotinate adenine dinucleotide (NaAD).

**Figure 8:**
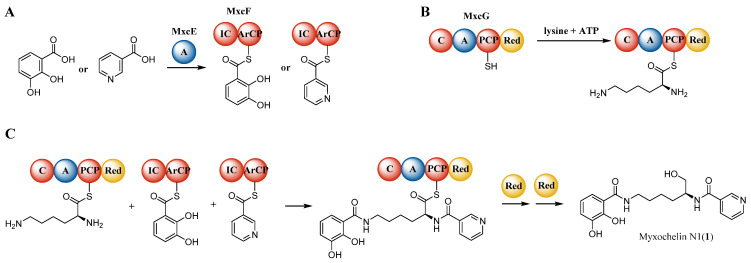
Proposed biosynthesis of **1** similar to [11]. (**A**) Activation and loading of DHBA or nicotinic acid to MxcF; (**B**) Activation and loading of lysine to MxcG; (**C**) Condensation and reductive release of the product **1**.

Based on the elucidated chemical structure of **1**, we propose the natural product to be biosynthesized as shown in Figure 8. MxcE activates nicotinic acid and DHBA by adenylation and loads it to the ArCP domain of MxcF (Figure 8A). The non-ribosomal peptide synthetase (NRPS) subunit MxcG activates and loads lysine (Figure 8B) and further condenses nicotinic acid and DHBA with the two primary amino groups of l-lysine (Figure 8C). The NADPH-dependent reductase domain of MxcG finally releases an aldehyde intermediate from the assembly line by reduction of the thioester and further reduces it to form **1.** The biosynthesis of **2** and **3** occurs likewise with the other (**2**) or both (**3**) primary amino groups of l-lysine substituted with nicotinic acid instead of DHBA.

## 3. Discussion

In this study, we report the isolation and structure elucidation of **1** and **3**, novel secondary metabolites featuring an unusual nicotinic acid moiety, from crude organic extracts of the myxobacterium *Corallococcus* sp. MCy9049 as well as the total synthesis of **1**, **2**, and the artificial derivatives **9c**–**9i**.

Whenever new variants of known natural products—such as nicotinic acid containing congeners of myxochelins in this study—are found, the question emerges how their appearance could be explained by the underlying biosynthetic pathway. From an evolutionary perspective, plausible explanations include the mutational change of substrate specificity in a building block-activating domain to recruit new moieties, possibly following a duplication of the domain or the entire biosynthetic gene cluster. However, the combined evidence from metabolome- and genome-based analysis spanning 105 and 20 myxobacteria, respectively, led us to dismiss these hypothetical explanations in this case. Firstly, we found no indication of a second myxochelin biosynthetic gene cluster in any publicly accessible myxobacterial genome; secondly, the loading module MxcE seems highly conserved across the myxobacteria clade and specificity-conferring residues show little variability; and lastly, production of new myxochelins **1**–**3** appears stringently coupled to formation of myxochelin A as judged on the basis of LC-MS analysis. We therefore reason that the nicotinic acid containing myxochelin congeners **1**–**3** reported here are common byproducts of myxochelin A synthesis, wherein their formation is enabled by substrate promiscuity of the A domain MxcE and biogenic precursor availability. A similar production mode depending on availability of nicotinic acid as precursor has also been assumed for asperphenamate biosynthesis in *Penicillium astrolabium* [32].

Nicotinic acid is a rarely occurring precursor for biosynthesis of natural products. Among the few known exceptions are a fungal polyketide synthase (PKS) that incorporates nicotinyl-CoA for meroterpenoid biosynthesis [33], a PKS from a marine mollusk that uses nicotinic acid as a starter unit to synthesize 3-alkylpyridine alkaloids [34], an NRPS-unit from *Micromonospora* sp. that incorporates nicotinic acid to produce kosinostatin [35], and a fungal NRPS that uses either benzoic acid or nicotinic acid for the biosynthesis of asperphenamates [32]. Furthermore, also in the biosynthesis of bacillibactins, the incorporation of nicotinic or benzoic acid instead of DHBA has been reported and assumed to happen due to a promiscuous DhbE [36], which has a pairwise identity of 67.7% (coverage 99.1%) to MxcE as well as the identical A domain Stachelhaus specificity-conferring code of PLPAQGVVNK (Table S2).

In myxochelin biosynthesis, it has been demonstrated previously that the enzymatic promiscuity of MxcE allows the activation and loading of several benzoic acid derivatives to the assembly line to form precursor-derived non-natural myxochelin derivatives [12,13,14]. However, incorporation of heteroaromatic carboxylic acid precursors to form natural or artificial myxochelins has so far not been described. Unlike in bacillibactin biosynthesis, where also picolinic acid can be incorporated [37], feeding of pyridinecarboxylic acids only gave relevant yields for nicotinic acid and nicotinamide. This might be an effect of either differences in enzyme promiscuity between DhbE and MxcE, or due to precursor availability as nicotinic acid and nicotinamide, can be actively transported into bacterial cells by niacin transport proteins NiaX, PnuC, NiaP, or NiaY [38]. Surprisingly, supplementation with nicotinamide in our study resulted in about 3-fold higher yield of **1**–**3** compared to nicotinic acid, which is likely due to decreased cell density in the nicotinic acid-supplemented culture. This is consistent with the finding that also the peak area of myxochelin A is about 3-fold lower with nicotinic acid than with nicotinamide and in the control (Figure 2).

The nicotinic acid containing myxochelins **9a** and **9b** as well as the synthetic analogues **9c**–**9i** displayed no antimicrobial activity at concentrations up to 128 µg/mL against a small test panel of bacteria and fungi. The reported antibacterial activity of myxochelin A [16] and other catecholate-type iron chelators [39] is attributed to iron competition, and thus, dependent on the presence of both DHBA moieties. As **1**–**3/9a**–**9i** are lacking one catecholate moiety needed for iron chelation, these compounds are presumably not functioning as siderophores anymore. This is supported by the finding that the peak area of myxochelin A of cultures supplemented with nicotinamide versus the respective control barely differs (Figure 2). Natural myxochelin A inhibits the growth of various cancer cell lines [9,23]. Encouragingly, three of the new synthetic derivatives (**9c**, **9f**, **9g**) also displayed prominent antiproliferative activity against human cancer cell lines at concentrations in the one-digit µg/mL range.

## 4. Conclusions

Taken together, the combined evaluation of metabolome and genome data in this study supports the notion that **1**–**3** are previously overlooked byproducts of myxochelin A biosynthesis, which are commonly produced by myxobacteria upon precursor availability. Although the biological function of these new myxochelin congeners remains elusive at present, the presented discovery of **1**–**3**, featuring an unusual nicotinic acid moiety, as well as the total synthesis of **9a**–**9i**, set the stage for further biosynthetic studies, the generation of new myxochelin derivatives, and identification of the biological function of **1**–**3**.

## 5. Materials and Methods

### 5.1. Myxobacterial Fermentation and Small-Scale Precursor Feeding

*Cystobacterineae* species *Corallococcus* sp. MCy9049 was routinely cultivated at 30 °C in VY/2S medium (1.0% baker’s yeast (F. X. Wieninger GmbH, Passau, Germany), 0.5% soluble starch (Carl Roth GmbH, Karlsruhe, Germany), 0.1% CaCl_2_ × 2 H_2_O (VWR International GmbH, Darmstadt, Germany), 0.1% MgSO_4_ × 7 H_2_O (Grüssing GmbH, Filsum, Germany), 10 mM TRIS (Sigma-Aldrich Chemie GmbH, Taufkirchen, Germany) adjusted to pH 7.2 with 1 M HCl before autoclaving). Liquid cultures were grown in Erlenmeyer shake flasks without baffles on an orbital shaker at 180 rpm for seven days. Feeding of precursors was performed by cultivating the strain in 50 mL VY/2S medium using 1 mL inoculum (2% inoculum volume). The cultures were supplemented with 1 mL (*v/v*) sterile amberlite resin XAD-16 (Sigma-Aldrich Chemie GmbH, Taufkirchen, Germany) and with either 500 µL of a 0.1 m aqueous solution of nicotinic acid, nicotinamide, or picolinic acid, or with 1.25 mL of a 0.04 m solution of isonicotinic acid (abcr GmbH, Karlsruhe, Germany), resulting in a final concentration of 1 mm each. After seven days, the combined cells and resin were separated from the supernatant by centrifugation. While the supernatant was discarded, the pellet was extracted with 50 mL methanol, stirred for 1 h, and filtered through filter paper. After the solvent was evaporated under vacuum, the extracts were redissolved in 1.5 mL of methanol, further diluted (1:5 with methanol, *v/v*) and centrifuged, and the supernatant was analyzed by high-performance liquid chromatography (HPLC) coupled to a mass spectrometer (MS) (Bruker Daltonics, Billerica, MA, USA).

### 5.2. Analysis of the Secondary Metabolome of Bacterial Crude Extracts

The secondary metabolome of bacterial crude extracts was analyzed on a Dionex UltiMate 3000 rapid separation liquid chromatography (RSLC) system (Thermo Fisher Scientific, Waltham, MA, USA) coupled to a Bruker maXis 4G ultra-high-resolution quadrupole time-of-flight (UHR-qTOF) MS equipped with a high-resolution electrospray ionization (HRESI) source (Bruker Daltonics, Billerica, MA, USA). Separation of 1 µL sample was achieved with a linear 5–95% gradient of acetonitrile with 0.1% formic acid in ddH_2_O with 0.1% formic acid on an ACQUITY BEH C18 column (100 × 2.1 mm, 1.7 µm d_p_) (Waters, Eschborn, Germany) at a flow rate of 0.6 mL/min and 45 °C over 18 min with detection by a diode array detector at 200–600 nm. The LC flow was split to 75 µL before entering the mass spectrometer. Mass spectrograms were acquired in centroid mode ranging from 150–2500 *m/z* at an acquisition rate of 2 Hz in positive MS mode. Source parameters were set to 500 V end plate offset; 4000 V capillary voltage; 1 bar nebulizer gas pressure; 5 L/min dry gas flow; and 200 °C dry gas temperature. Calibration was conducted automatically before every HPLC-MS run by injection of sodium formate and calibration on the respective clusters formed in the ESI source. All MS analyses were acquired in the presence of the lock masses C_12_H_19_F_12_N_3_O_6_P_3_, C_18_H_19_O_6_N_3_P_3_F_2_, and C_24_H_19_F_36_N_3_O_6_P_3_, which generate the [M + H]^+^ ions of 622.0289; 922.0098, and 1221.9906. The HPLC-MS system was operated by HyStar 5.1 (Bruker Daltonics, Billerica, MA, USA) and LC chromatograms as well as UV spectra and mass spectrograms were analyzed with DataAnalysis 4.4 (Bruker Daltonics, Billerica, MA, USA).

For statistical metabolomics analysis, both the strain and medium blanks were cultivated and extracted in triplicates as described before. Each extract was measured as technical duplicates giving a total number of six replicates each for the bacterial and medium blank extracts. T-ReX-3D molecular feature finder of MetaboScape 6.0.2 (Bruker Daltonics, Billerica, MA, USA) was used to obtain molecular features. Detection parameters were set to intensity threshold 5 × 10^3^ and minimum peak length of five spectra. Identification of bacterial features was performed with the built in t-test routine and filtered to appearance in all six bacterial extracts and in none of medium blank extracts.

### 5.3. Compound Isolation

For compound isolation, *Corallococcus* sp. MCy9049 was cultivated in 10 L VY/2S medium containing 1 mm Nicotinamide and 2% XAD-16 resin (*v/v*) as described before. After seven days of fermentation, combined cells and resin were harvested by centrifugation and extracted with 1 L methanol twice. After drying under vacuum, the extract was redissolved in 500 mL methanol and partitioned using 500 mL *n*-hexane. The methanol layer was dried, redissolved in 500 mL ddH_2_O, and further partitioned using 500 mL chloroform. The aqueous layer was once more partitioned using 500 mL ethyl acetate. The ethyl acetate layer was dried to yield 55.2 mg of extract and redissolved in 2 mL methanol. Purification was performed using a Dionex UltiMate 3000 semi-preparative system equipped with an automated fraction collector (Thermo Fisher Scientific, Waltham, MA, USA). Compound separation was achieved with a gradient of acetonitrile with 0.1% formic acid (B) in ddH_2_O with 0.1% formic acid (A) on an ACQUITY BEH C18 column (250 × 10 mm, 5 µm d_p_) (Waters, Eschborn, Germany) at a flow rate of 5.0 mL/min and 45 °C. The initial gradient was held at 5% B for 1 min and then elevated to 35% B within 15 min. After that, the B level was increased to 95% and held there for one minute. Finally, the gradient was ramped back to 5% B in 1 min and re-equilibrated for the next injection for 3 min. Detection was performed using the 3D plot of a DAD detector by absorption at 220, 245, 260, and 310 nm. The isolated pure compounds were dried by lyophilization.

### 5.4. Structure Elucidation, Chiroptical and Circular Dichroism Measurements

The chemical structures of all the compounds were determined via multidimensional NMR analysis. ^1^H-NMR, ^13^C-NMR, and 2D spectra were recorded at 700 and 500 MHz (^1^H)/175 and 126 MHz (^13^C), conducted in the Ascend 700 spectrometer using a cryogenically cooled triple resonance probe (Bruker BioSpin, Rheinstetten, Germany) or in the Bruker Avance Neo 500 MHz, equipped with a Prodigy Cryo-probe. Samples were dissolved in methanol-d_4_, acetone-d_6_, or dimethyl sulfoxide-d_6_. Chemical shifts are reported in ppm relative to tetramethylsilane; the solvent was used as the internal standard. Coupling constants are reported in Hertz (Hz). Multiplicity is reported with the usual abbreviations (s: singlet, br s: broad singlet, d: doublet, dd: doublet of doublets, ddd: doublet of doublet of doublets, t: triplet, dt: doublet of triplets, q: quartet, p: pentet, dp: doublet of pentets, m: multiplet). Chiroptical measurements of all the compounds in MeOH ([α]_D_) were obtained on a model 341 polarimeter (PerkinElmer Inc. Waltham, MA, USA) in a 100 × 2 mm cell at 25 °C. Circular dichroism measurements were performed for **1** and its synthetic analogue **9a** at 1.0 mg/mL in MeOH (190–400 nm) with the *J*-1500 CD spectrophotometer (JASCO, Easton, MD, USA).

*Myxochelin N1* (**1**), yellow oil (0.3 mg), ${\left[\alpha \right]}_{D}^{20}$ –18.6 (c 0.025, MeOH); ^1^H NMR (700 MHz, methanol-*d*_4_): δ 8.96 (d, *J* = 2.2 Hz, 1H), 8.66 (brd, *J* = 8.2 Hz, 1H), 8.23 (brd, *J* = 7.7 Hz, 1H), 7.49 (dd, *J* = 8.0, 5.0 Hz, 1H), 7.16 (d, *J* = 8.1 Hz, 1H), 6.90 (d, *J* = 8.0 Hz, 1H), 6.67 (t, *J* = 8.0 Hz, 1H), 4.15 (m, 1H), 3.63 (brd, *J* = 3.4 Hz, 1H), 3.61 (brd, *J* = 3.6 Hz, 1H), 3.39 (t, *J* = 7.0 Hz, 2H), 1.72 (m, 2H), 1.63 (m, 2H), 1.49 (m, 2H); ^13^C NMR (126 MHz, methanol-*d*_4_): δ 170.1, 166.7, 151.0, 148.6, 147.7, 145.9, 135.7, 130.9, 123.6, 118.1, 118.1, 117.1, 115.3, 64.4, 53.2, 39.4, 30.2, 28.9, 22.6; HRMS (ESI) (*m/z*): calcd for C_19_H_24_N_3_O_5_ 374.1710, found 374.1710 [M + H]^+^.

*Myxochelin N3* (**3**), yellow oil (0.1 mg), ${\left[\alpha \right]}_{D}^{20}$ −26.4 (c 0.025, MeOH); ^1^H NMR (700 MHz, methanol-*d*_4_): δ 8.95 (dd, *J* = 2.2 Hz, 1H), 8.65 (dt, *J* = 8.4, 1.6 Hz, 1H), 8.20 (ddt, *J* = 8.4, 2.2, 2.0 Hz, 1H), 7.51 (dd, *J* = 7.9, 2.3 Hz, 1H), 4.15 (m, 1H), 4.15 (m, 1H), 3.62 (dd, *J* = 10.7, 5.4 Hz, 1H), 3.61 (dd, *J* = 10.6, 5.2 Hz, 1H), 3.42 (m, 2H), 1.74 (m, 2H), 1.64 (m, 2H), 1.49 (m, 2H); ^13^C NMR (126 MHz, methanol-*d*_4_): δ 167.2, 151.7, 148.2, 136.4, 131.2, 123.6, 64.8, 53.7, 40.4, 30.2, 29.2, 23.4.; HRMS (ESI) (*m/z*): calcd for C_18_H_23_N_4_O_3_ 343.1765, found 343.1766 [M + H]^+^.

### 5.5. Chemistry

#### 5.5.1. General Procedure for Synthetic Step **a**

Fmoc-l-lysine hydrochloride (1 eq) was suspended in ethanol and was cooled to 0 °C. Then, chlorotrimethylsilane (2 eq) was added and the resulting suspension was allowed to warm up to r. t. and it was stirred for 16 h. The remaining ethanol and chlorotrimethylsilane were evaporated under reduced pressure. The crude product of **4** was obtained as a white powder and was used without further purification.

#### 5.5.2. General Procedure for Synthetic Step **b**

Fmoc-l-lysine ethyl ester hydrochloride (**4**) (1 eq) was dissolved in dry DMF (5.0 mL). Subsequently, 2,3-dimethoxybenzoic acid (2 eq) or nicotinic acid (2 eq) and HATU (2 eq) were dissolved in dry DMF (10.0 mL) and were added to the reaction flask. The resulting solution was cooled to 0 °C and TEA (2.5 eq) was added dropwise over 5 min. Then, the solution was allowed to warm to r. t. and it was stirred for 16 h. After completion of the reaction (monitored via LC-MS), the reaction solution was poured into ice water (100 mL). The mixture was exhaustively extracted with ethyl acetate (3 × 50 mL). The organic layers were combined, dried over sodium sulfate, and filtered. After evaporation of the solvent, the crude product was purified by automated flash chromatography using petroleum ether/ethyl acetate (100%:0% → 0%:100%) as eluent. The product was a yellow oil.

#### 5.5.3. General Procedure for Synthetic Step **c**

To the ethyl *N*^2^-Fmoc-*N*^6^-aryl-l-lysinate compound **5a** or **5b** (1 eq) a mixture of DMA and DCM (1:1 *v/v*) was added and the resulting solution was stirred at r. t. for 2 h. Then, the solvent and residual dimethylamine was removed under reduced pressure. Subsequently, the residue was dissolved in a mixture of saturated ammonium chloride solution and ethyl acetate (1:1 *v/v*, 50 mL), the aqueous phase was extracted with ethyl acetate (3 × 50 mL). Afterwards, saturated sodium bicarbonate solution was added to the aqueous phase until the pH was set to ~8. Then, extraction of the aqueous phase was repeated with ethyl acetate (3 × 50 mL). The organic phases were combined, dried over sodium sulfate, and filtered. After the evaporation of the solvent, the crude product was obtained as a viscous yellow oil and was used without further purification.

#### 5.5.4. General Procedure for Synthetic Step **d**

The ethyl *N*^6^-aryl-l-lysinate compound **6a** or **6b** (1 eq) was dissolved in dry DMF (5.0 mL). Subsequently, HATU (2 eq) and nicotinic acid (2 eq) or a benzoic acid derivative (2 eq) were dissolved in dry DMF (10.0 mL) and were added to the reaction flask. Lastly, TEA (2.5 eq) was added dropwise and the solution was stirred at r. t. for 16 h. After completion, the reaction mixture was poured into of ice water (100 mL). The mixture was exhaustively extracted with ethyl acetate (3 × 50 mL). The organic layers were combined, dried over sodium sulfate and filtered. After evaporation of the solvent, the crude product was taken forward either without further purification or it was purified by automated reverse-phase flash chromatography, using water/acetonitrile (95%:5% → 5%:95%) as eluent. The product was a viscous yellow oil.

#### 5.5.5. General Procedure for Synthetic Step **e**

Ethyl *N^6^*-(2,3-dimethoxybenzoyl)-l-lysinate **6a** (1 eq) was dissolved in dry DMF (5.0 mL). Subsequently, a Fmoc-protected aminobenzoic acid derivative (2 eq) and HATU (2 eq) were dissolved in dry DMF (10.0 mL) and were added to the reaction flask. The resulting solution was cooled to 0 °C and TEA (2.5 eq) was added dropwise over 5 min and the solution was stirred at r. t. for 16 h. After completion, DMA (5.0 mL) was added and the solution was stirred at r. t. for 2 h. Then, the reaction mixture was poured into ice water (100 mL). The mixture was exhaustively extracted with ethyl acetate (3 × 50 mL). The organic layers were combined, dried over sodium sulfate, and filtered. After evaporation of the solvent, the crude product was taken forward without further purification or it was purified by automated reverse-phase flash chromatography, using water/acetonitrile (95%:5% → 5%:95%) as eluent. The product was a viscous yellow oil.

#### 5.5.6. General Procedure for Synthetic Step **f**

Ethyl *N*^2^-aryl-*N*^6^-aryl-l-lysinate **7a**–**7g** (1 eq) was dissolved in dry THF (1.0 mL) under a N_2_ atmosphere. Then, lithium borohydride (2 M in THF, 1 eq) was added at r. t. and the resulting mixture was stirred for 2 h. After completion, the reaction was quenched by addition of saturated aqueous ammonium chloride solution (3 mL). The resulting mixture was stirred at r. t. for 30 min. Then, the solution was exhaustively extracted with DCM (3 × 20 mL). The organic layers were combined, dried over sodium sulfate, and filtered. After evaporation of the solvent, the crude product was purified by automated flash chromatography, using DCM/methanol (100%:0% → 90%:10%) as eluent. The product was a viscous yellow oil.

#### 5.5.7. General Procedure for Synthetic Step **g**

The dimethoxybenzamide containing compound **8a**–**8g** (1 eq) was dissolved in dry DCM (1.0 mL) under a N_2_ atmosphere. Boron tribromide (1 m in DCM, 4 eq) was added and the reaction mixture was stirred at r. t. for 16 h. After completion, the reaction was quenched by addition of water (2 mL) and the solvent mixture was evaporated to dryness. Then, the crude product was purified by preparative HPLC, using water/acetonitrile (95%:5% → 80%:20%) as eluent. The fractions with the product were collected and freeze dried. The product was a light-yellow powder.

### 5.6. Characterization of Synthetic Intermediates and Final Products

Ethyl Fmoc-l-lysinate (**4**), General procedure **a** was followed with Fmoc-l-lysine hydrochloride (2.43 g, 6.0 mmol) and chlorotrimethylsilane (1.50 mL, 12.0 mmol) to afford intermediate **4** as a white solid (yield: 2.60 g, quant.). ^1^H NMR (500 MHz, DMSO-*d*_6_): δ 7.90 (d, *J* = 7.5 Hz, 2H), 7.82 (br s, 2H), 7.71 (t, *J* = 7.5 Hz, 2H), 7.42 (t, *J* = 7.4 Hz, 2H), 7.33 (t, *J* = 7.4 Hz, 2H), 4.39–4.17 (m, 3H), 4.09 (qd, *J* = 7.2, 2.3 Hz, 2H), 3.97 (ddd, *J* = 9.6, 7.8, 4.9 Hz, 1H), 2.75 (h, *J* = 6.5 Hz, 2H), 1.74–1.27 (m, 6H), 1.17 (t, *J* = 7.1 Hz, 3H); ESI-MS (*m/z*): calcd for C_23_H_29_N_2_O_4_ 397.21, found 397.14 [M + H]^+^.

Ethyl N^2^-Fmoc-N^6^-(2,3-dimethoxybenzoyl)-l-lysinate (**5a**). General procedure **b** was followed with **4** (1.95 g, 4.5 mmol), 2,3-dimethoxybenzoic acid (1.64 g, 9.0 mmol), HATU (3.42 g, 9.0 mmol), and TEA (2.50 mL, 18 mmol) to afford intermediate **5a** as a yellow oil (2.28 g, 90%, purity >83%). ^1^H NMR (500 MHz, DMSO- *d*_6_): δ 8.19 (t, *J* = 5.8 Hz, 1H), 7.89 (d, *J* = 7.5 Hz, 2H), 7.76 (d, *J* = 7.8 Hz, 1H), 7.71 (dd, *J* = 7.5, 2.9 Hz, 2H), 7.41 (t, *J* = 7.5 Hz, 2H), 7.32 (t, *J* = 7.4 Hz, 2H), 7.23–7.16 (m, 1H), 7.16–7.05 (m, 1H), 4.35–4.18 (m, 3H), 4.18–3.94 (m, 3H), 3.81 (s, 3H), 3.74 (s, 3H), 3.26–3.21 (m, 2H), 1.88–1.61 (m, 2H), 1.52 (dq, *J* = 14.3, 6.9 Hz, 2H), 1.40 (td, *J* = 14.2, 12.5, 8.1 Hz, 2H), 1.16 (t, *J* = 7.0 Hz, 3H); ^13^C NMR (126 MHz, DMSO-*d6*): δ 172.5, 165.4, 156.1, 152.5, 146.2, 143.8, 140.8, 130.3, 127.7, 127.1, 125.2, 124.0, 120.6, 120.1, 114.5, 65.6, 60.9, 60.4, 55.9, 54.0, 46.6, 38.6, 30.4, 28.6, 23.0, 14.1; HRMS (ESI) (*m/z*): calcd for C_32_H_37_N_2_O_7_ 561.2601, found 561.2584 [M + H]^+^.

Ethyl N^2^-Fmoc-N^6^-nicotinoyl**-**l-lysinate (**5b**). General procedure **b** was followed with **4** (650 mg, 1.5 mmol), nicotinic acid (369 mg, 3.0 mmol), HATU (1.14 g, 3.0 mmol), and TEA (0.850 mL, 6.0 mmol) to afford intermediate **5b** as a yellow oil (701 mg, 93%, purity >87%). ^1^H NMR (500 MHz, methanol-*d*_4_): δ 8.83 (d, *J* = 2.3 Hz, 1H), 8.51 (dd, *J* = 4.9, 1.6 Hz, 1H), 8.12 (dt, *J* = 8.0, 2.0 Hz, 1H), 7.63 (d, *J* = 7.5 Hz, 2H), 7.50 (t, *J* = 7.7 Hz, 2H), 7.40 (dd, *J* = 8.0, 5.0 Hz, 1H), 7.22 (t, *J* = 7.5 Hz, 2H), 7.14 (t, *J* = 7.5 Hz, 2H), 4.21–4.10 (m, 2H), 4.07–3.98 (m, 2H), 3.94 (q, *J* = 7.1 Hz, 2H), 3.29 (t, *J* = 7.0 Hz, 2H), 1.68–1.29 (m, 6H), 1.15 (t, *J* = 7.3 Hz, 3H); ^13^C NMR (126 MHz, CDCl_3_): δ 172.5, 166.0, 165.9, 156.4, 152.2, 148.1, 144.0, 143.7, 141.4, 135.2, 130.4, 130.3, 127.9, 127.2, 125.2, 123.5, 120.1, 67.2, 61.8, 53.5, 47.2, 39.7, 32.7, 28.6, 22.6, 14.3; HRMS (ESI) (*m/z*): calcd for C_29_H_32_N_3_O_5_ 502.2342, found 502.2323 [M + H]^+^.

Ethyl N6-(2,3-dimethoxybenzoyl)-l-lysinate (**6a**). General procedure **c** was followed with **5a** (2.28 g, 4.1 mmol) and DMA/DCM (1:1 *v/v*, 9 mL) to afford intermediate **6a** as a yellow oil (2.34 g, 70%, purity >56%). HRMS (ESI) (*m/z*): calcd for C_17_H_27_N_2_O_5_ 339.1920, found 339.1904 [M + H]^+^.

Ethyl N6-nicotinoyl-l-lysinate (**6b**). General procedure **c** was followed with **5b** (701 mg, 1.4 mmol) and DMA/DCM (1:1 *v/v*, 3 mL) to afford crude intermediate **6b** as a yellow oil (not isolated, purity >53%). HRMS (ESI) (*m/z*): calcd for C_14_H_22_N_3_O_3_ 280.1661, found 280.1646 [M + H]^+^.

Ethyl N6-(2,3-dimethoxybenzoyl)-N2-nicotinoyl-l-lysinate (**7a**). General procedure **d** was followed with **6a** (1.53 g, 4.5 mmol), nicotinic acid (1.11 g, 9.0 mmol), HATU (3.42 g, 9.0 mmol), and TEA (2.50 mL, 18.0 mmol) and purified by automated reverse-phase flash chromatography to afford intermediate **7a** as a yellow oil (850 mg, 43%). ^1^H NMR (500 MHz, methanol-*d*_4_): δ 8.99 (d, *J* = 2.3 Hz, 1H), 8.69 (dt, *J* = 4.9, 1.2 Hz, 1H), 8.26 (dt, *J* = 8.0, 2.2 Hz, 1H), 7.53 (dd, *J* = 8.0, 4.9 Hz, 1H), 7.26 (dd, *J* = 7.5, 1.9 Hz, 1H), 7.18–7.08 (m, 2H), 4.67–4.56 (m, 1H), 4.21 (q, *J* = 7.1 Hz, 2H), 3.87 (s, 3H), 3.83 (s, 3H), 3.45–3.39 (m, 2H), 2.08–1.86 (m, 2H), 1.70 (m, 2H), 1.67–1.51 (m, 2H), 1.28 (t, *J* = 7.1, 3H); ^13^C NMR (126 MHz, methanol-*d_4_*): δ 173.7, 168.7, 168.2, 154.2, 152.8, 149.3, 148.4, 137.2, 131.6, 129.5, 125.4, 125.1, 122.2, 116.4, 62.5, 61.8, 56.6, 54.6, 40.2, 31.8, 30.0, 24.5, 14.5; HRMS (ESI) (*m/z*): calcd for C_23_H_30_N_3_O_6_ 444.2135, found 444.2116 [M + H]^+^.

Ethyl N^2^-(2,3-dimethoxybenzoyl)-N^6^-nicotinoyl-l-lysinate (**7b**). General procedure **d** was followed with **6b** (508 mg, 1.5 mmol), 2,3-dimethoxybenzoic acid (546 mg, 3.0 mmol), HATU (1.14 g, 3.0 mmol), and TEA (0.850 mL, 6.0 mmol) and purified by automated reverse-phase flash chromatography to afford intermediate **7b** as a yellow oil (701 mg, 93%, purity >65%). ^1^H NMR (500 MHz, methanol-*d*_4_): δ 8.93 (d, *J* = 2.3 Hz, 1H), 8.66 (dd, *J* = 4.9, 1.7 Hz, 1H), 8.20 (dt, *J* = 8.1, 2.0 Hz, 1H), 7.51 (dd, *J* = 8.0, 5.0 Hz, 1H), 7.37 (dd, *J* = 7.7, 1.7 Hz, 1H), 7.20 (dd, *J* = 8.2, 1.8 Hz, 1H), 7.14 (t, *J* = 8.0 Hz, 1H), 4.70–4.63 (m, 1H), 4.22 (qd, *J* = 7.1, 1.9 Hz, 2H), 3.89 (br s, 6H), 3.46–3.37 (m, 2H), 2.07–1.96 (m, 1H), 1.94–1.83 (m, 1H), 1.77–1.63 (m, 2H), 1.57–1.43 (m, 2H), 1.28 (t, *J* = 7.1 Hz, 3H); ^13^C NMR (126 MHz, DMSO-*d_6_*): δ 172.0, 165.4, 164.7, 152.6, 151.7, 148.3, 146.5, 134.9, 130.1, 128.7, 124.1, 123.4, 120.8, 115.2, 61.0, 60.6, 56.0, 52.3, 38.9, 30.7, 28.6, 22.8, 14.1; HRMS (ESI) (*m/z*): calcd for C_23_H_30_N_3_O_6_ 444.2135, found 444.2119 [M + H]^+^.

Ethyl N^2^-(2-chlorobenzoyl)-N^6^-(2,3-dimethoxybenzoyl)-l-lysinate (**7c**). General procedure **d** was followed with **6a** (676 mg, 2.0 mmol), 2-chlorobenzoic acid (312 mg, 2.0 mmol), HATU (760 mg, 2.0 mmol), and TEA (0.600 mL, 4.0 mmol) to afford intermediate **7c** as a yellow oil (161 mg, 17%). ^1^H NMR (500 MHz, DMSO-*d*_6_): δ 8.82 (d, *J* = 7.5 Hz, 1H), 8.20 (t, *J* = 5.6 Hz, 1H), 7.51–7.48 (m, 1H), 7.48–7.43 (m, 1H), 7.41–7.36 (m, 2H), 7.14 (dd, *J* = 6.7, 3.2 Hz, 1H), 7.11–7.06 (m, 2H), 4.35 (ddd, *J* = 9.6, 7.4, 4.8 Hz, 1H), 4.21–4.02 (m, 2H), 3.78 (s, *3*H), 3.71 (s, 3H) 3.24 (m, 2H), 1.84–1.41 (m, 6H), 1.21 (t, *J* = 7.1 Hz, 3H); ^13^C NMR (126 MHz, DMSO-*d_6_*): δ 172.3, 167.0, 165.9, 153.0, 146.6, 136.9, 131.4, 130.8, 130.5, 130.1, 129.4, 127.5, 124.5, 121.0, 115.0, 61.4, 61.0, 56.4, 53.0, 39.1, 30.7, 29.1, 23.4, 14.6; HRMS (ESI) (*m/z*): calcd for C_24_H_30_ClN_2_O_6_ 477.1792, found 477.1776 [M + H]^+^.

Ethyl N^2^-(2,3-dichlorobenzoyl)-N^6^-(2,3-dimethoxybenzoyl)-l-lysinate (**7d**). General procedure **d** was followed with **6a** (676 mg, 2.0 mmol), 2,3-dichlorobenzoic acid (380 mg, 2.0 mmol), HATU (760 mg, 2.0 mmol), and TEA (0.600 mL, 4.0 mmol) to afford intermediate **7d** as a yellow oil (51.0 mg, 5%). ^1^H NMR (500 MHz, DMSO-*d*_6_): 8.91 (d, *J* = 7.3 Hz, 1H), 8.72 (dd, *J* = 4.8, 1.7 Hz, 1H), 8.30–8.02 (m, 2H), 7.51 (ddd, *J* = 8.0, 4.9, 0.9 Hz, 1H), 7.13 (dd, *J* = 6.9, 3.0 Hz, 1H), 7.10–6.94 (m, 2H), 4.40 (ddd, *J* = 9.1, 7.2, 5.6 Hz, 1H), 4.11 (q, *J* = 7.1, 2H), 3.81 (s, 3H), 3.72 (s, 3H), 3.25 (q, *J* = 6.6 Hz, 2H), 1.91–1.37 (m, 6H), 1.19 (t, *J* = 7.1 Hz, 3H); HRMS (ESI) (*m/z*): calcd for C_24_H_29_Cl_2_N_2_O_6_ 511.1403, found 511.1388 [M + H]^+^.

Ethyl N^2^-(2-aminobenzoyl)-N^6^-(2,3-dimethoxybenzoyl)-l-lysinate (**7e**). General procedure **e** was followed with **6a** (676 mg, 2.0 mmol), *N*-Fmoc-2-aminobenzoic acid (**SI-1**) (718 mg, 2.0 mmol), HATU (760 mg, 2.0 mmol), and TEA (0.600 mL, 4.0 mmol) to afford intermediate **7e** as a yellow oil (128 mg, 14%, purity >74%). ^1^H NMR (500 MHz, DMSO-*d*_6_): δ 8.38 (d, *J* = 7.2 Hz, 1H), 8.20 (t, *J* = 5.8 Hz, 1H), 7.57 (dd, *J* = 8.0, 1.6 Hz, 1H), 7.18–7.01 (m, 4H), 6.68 (dd, *J* = 8.4, 1.2 Hz, 1H), 6.49 (ddd, *J* = 8.1, 7.1, 1.2 Hz, 1H), 6.35 (s, 2H), 4.31 (q, *J* = 7.2 Hz, 1H), 4.17–4.01 (m, 2H), 3.82 (s, 3H), 3.73 (s, 3H), 3.25 (q, *J* = 6.5 Hz, 2H) 1.80 (t, *J* = 7.6 Hz, 2H), 1.59–1.24 (m, 4H), 1.18 (t, *J* = 7.1 Hz, 3H); HRMS (ESI) (*m/z*): calcd for C_24_H_32_N_3_O_6_ 458.2291, found 458.2273 [M + H]^+^.

Ethyl N^2^-(2-amino-3-chlorobenzoyl)-N^6^-(2,3-dimethoxybenzoyl)-l-lysinate (**7f**). General procedure **e** was followed with **6a** (676 mg, 2.0 mmol), *N*-Fmoc-2-amino-3-chlorobenzoic acid (**SI-2**) (786 mg, 2.0 mmol), HATU (760 mg, 2.0 mmol), and TEA (0.600 mL, 4.0 mmol) to afford intermediate **7f** as a yellow oil (125 mg, 13%). ^1^H NMR (500 MHz, DMSO-*d*_6_): δ 8.92 (d, *J* = 7.5 Hz, 1H), 8.20 (t, *J* = 5.8 Hz, 1H), 7.71 (dd, *J* = 8.0, 1.6 Hz, 1H), 7.42 (t, *J* = 7.8 Hz, 1H), 7.35 (dd, *J* = 7.6, 1.6 Hz, 1H), 7.18–7.05 (m, 3H), 4.36 (ddd, *J* = 9.6, 7.5, 4.8 Hz, 1H), 4.20–4.06 (m, 2H), 3.82 (s, 3H), 3.74 (s, 3H), 3.24 (qd, *J* = 6.7, 1.8 Hz, 2H), 1.89–1.65 (m, 2H), 1.63–1.38 (m, 4H), 1.21 (t, *J* = 7.1 Hz, 3H); ^13^C NMR (126 MHz, DMSO-*d_6_*): δ 171.7, 165.9, 165.5, 152.5, 146.2, 138.8, 132.1, 131.2, 130.3, 128.5, 128.2, 127.4, 124.0, 120.6, 114.5, 60.9, 60.6, 56.0, 52.5, 38.6, 30.2, 28.6, 22.9, 14.1; HRMS (ESI) (*m/z*): calcd for C_24_H_31_ClN_3_O_6_ 492.1901, found 492.1881 [M + H]^+^.

Ethyl N^2^-(2-amino-6-fluorobenzoyl)-N^6^-(2,3-dimethoxybenzoyl)-l-lysinate (**7g**). General procedure **e** was followed with **6a** (676 mg, 2.0 mmol), *N*-Fmoc-2-amino-6-fluorobenzoic acid (**SI-3**) (754 mg, 2.0 mmol), HATU (760 mg, 2.0 mmol), and TEA (0.600 mL, 4.0 mmol) to afford intermediate **7g** as a yellow oil (130 mg, 14%, purity >76%). ^1^H NMR (500 MHz, DMSO-*d*_6_): δ 8.57 (dd, *J* = 7.1, 2.0 Hz, 1H), 8.19 (dd, *J* = 7.0, 4.5 Hz, 1H), 7.91–7.78 (m, 1H), 7.76–7.57 (m, 1H), 7.44–7.23 (m, 1H), 7.14–7.02 (m, 1H), 6.50 (dd, *J* = 8.3, 0.9 Hz, 1H), 6.35–6.25 (m, 1H), 5.78 (s, 2H), 4.34 (ddd, *J* = 9.2, 7.0, 5.1 Hz, 1H), 4.17–4.07 (m, 2H), 3.82 (s, 3H), 3.74 (s, 3H), 3.23 (qd, *J* = 6.7, 2.6 Hz, 2H), 1.83–1.71 (m, 2H), 1.57–1.37 (m, 4H), 1.20 (t, *J* = 7.1 Hz, 3H); HRMS (ESI) (*m/z*): calcd for C_24_H_31_FN_3_O_6_ 476.2197, found 476.2180 [M + H]^+^.

*(S)*-*N*-(6-(2,3-Dimethoxybenzamido)-1-hydroxyhexan-2-yl)nicotinamide (**8a**). General procedure **f** was followed with **7a** (850 mg, 1.92 mmol) and lithium borohydride (2 M in THF, 0.950 mL) to afford intermediate **8a** as a yellow oil (330 mg, 43%). ^1^H NMR (500 MHz, methanol-*d*_4_): δ 8.97 (d, *J* = 2.3 Hz, 1H), 8.66 (dd, *J* = 4.9, 1.6 Hz, 1H), 8.23 (dt, *J* = 8.1, 2.0 Hz, 1H), 7.50 (dd, *J* = 8.0, 4.9 Hz, 1H), 7.26 (dd, *J* = 7.6, 1.9 Hz, 1H), 7.17–7.07 (m, 2H), 4.61 (d, *J* = 19.5 Hz, 1H), 4.16 (dq, *J* = 10.3, 5.3 Hz, 1H), 3.87 (s, 3H), 3.81 (s, 3H), 3.80 (d, *J* = 11.2 Hz, 1H), 3.54–3.35 (m, 4H), 1.86–1.44 (m, 6H); ESI-MS (*m/z*): calcd for C_21_H_28_N_3_O_5_ 402.20, found 402.06 [M + H]^+^.

*(S)*-*N*-(5-(2,3-Dimethoxybenzamido)-6-hydroxyhexyl)nicotinamide (**8b**). General procedure **f** was followed with **7b** (280 mg, 0.63 mmol) and lithium borohydride (2 M in THF, 0.31 mL) to afford intermediate **8b** as a yellow oil (91 mg, 36%). No ^1^H NMR spectrum was recorded; ESI-MS (*m/z*): calcd for C_21_H_28_N_3_O_5_ 402.20, found 402.08 [M + H]^+^.

*(S)*-*N*-(5-(2-Chlorobenzamido)-6-hydroxyhexyl)-2,3-dimethoxybenzamide (**8c**). General procedure **f** was followed with **7c** (161 mg, 0.34 mmol) and lithium borohydride (2 M in THF, 0.200 mL) to afford intermediate **8c** as a yellow oil (90.0 mg, 61%). ^1^H NMR (500 MHz, DMSO-*d*_6_): δ 8.18 (t, *J* = 5.7 Hz, 1H), 8.10 (d, *J* = 8.5 Hz, 1H), 7.47 (dd, *J* = 8.1, 1.3 Hz, 1H), 7.47–7.35 (m, 3H), 7.18–7.05 (m, 3H), 5.75 (s, 1H), 4.69 (t, *J* = 5.7 Hz, 1H), 3.82 (s, 3H), 3.75 (s, 3H), 3.46 (dt, *J* = 10.7, 5.4 Hz, 2H), 3.27–3.20 (m, 2H), 1.59–1.32 (m, 6H); HRMS (ESI) (*m/z*): calcd for C_22_H_28_ClN_2_O_5_ 435.1687, found 435.1668 [M + H]^+^.

*(S)*-2,3-Dichloro-N-(6-(2,3-dimethoxybenzamido)-1-hydroxyhexan-2-yl)benzamide (**8d**). General procedure **f** was followed with **7d** (51.0 mg, 0.10 mmol) and lithium borohydride (2 M in THF, 0.050 mL) to afford intermediate **8d** as a yellow oil (23.0 mg, 49%). Intermediate **8d** was taken to the next step without purification. No ^1^H NMR spectrum was recorded; ESI-MS (*m/z*): calcd for C_22_H_27_Cl_2_N_2_O_5_ 469.13, found 469.15 [M + H]^+^.

*(S)-N*-(5-(2-Aminobenzamido)-6-hydroxyhexyl)-2,3-dimethoxybenzamide (**8e**). General procedure **f** was followed with **7e** (128 mg, 0.28 mmol) and lithium borohydride (2 M in THF, 0.280 mL) to afford crude intermediate **7e** as a yellow oil (90.0 mg, 61%). Intermediate **8e** was taken to the next step without purification. No ^1^H NMR spectrum was recorded; ESI-MS (*m/z*): calcd for C_22_H_30_N_3_O_5_ 416.21, found 416.16 [M + H]^+^.

*(S)*-2-Amino-3-chloro-N-(6-(2,3-dimethoxybenzamido)-1-hydroxyhexan-2-yl)benzamide (**8f**). General procedure **f** was followed with **7f** (125 mg, 0.25 mmol) and lithium borohydride (2 M in THF, 0.160 mL) to afford intermediate **8f** as a yellow oil (57.0 mg, 51%, purity >70%). ^1^H NMR (500 MHz, DMSO-*d*_6_): δ 8.17 (t, *J* = 5.6 Hz, 1H), 7.97 (d, *J* = 8.5 Hz, 1H), 7.50 (dd, *J* = 7.9, 1.5 Hz, 1H), 7.37 (td, *J* = 7.6, 1.4 Hz, 1H), 7.16–7.04 (m, 3H), 6.56 (t, *J* = 7.8 Hz, 1H), 6.41 (d, *J* = 9.8 Hz, 2H), 4.69 (t, *J* = 5.8 Hz, 1H), 3.92 (td, *J* = 8.5, 8.0, 4.2 Hz, 1H), 3.81 (s, 3H), 3.72 (s, 3H), 3.47–3.40 (m, 2H), 3.23 (q, *J* = 6.7 Hz, 2H), 1.68–1.26 (m, 6H); ESI-MS (*m/z*): calcd for C_22_H_29_ClN_3_O_5_ 450.18, found 450.13 [M + H]^+^.

*(S)*-N-(5-(2-Amino-6-fluorobenzamido)-6-hydroxyhexyl)-2,3-dimethoxybenzamide (**8g**). General procedure **f** was followed with **7g** (130 mg, 0.27 mmol) and lithium borohydride (2 M in THF, 0.140 mL) to afford **8g** as a yellow oil (84.0 mg, 72%). ^1^H NMR (500 MHz, DMSO-*d*_6_): δ 8.17 (t, *J* = 3.85 Hz, 1H), 7.83 (dd, *J* = 8.7, 2.5 Hz, 1H), 7.13 (dd, *J* = 8.1, 2.0 Hz, 1H), 7.10 (d, *J* = 8.0 Hz, 1H), 7.09 (d, *J* = 8.0, Hz, 1H), 7.04 (dd, *J* = 8.3, 1.9 Hz, 1H), 6.49 (dd, *J* = 8.2, 1.0 Hz, 1H), 6.29 (ddt, *J* = 11.1, 8.1, 1.0 Hz, 1H), 5.80 (s, 1H), 5.76 (s, 1H), 4.73 (t, *J* = 5.5 Hz, 1H), 3.95–3.90 (m, 1H), 3.82 (s, 3H), 3.74 (s, 3H), 3.46–3.33 (m, 2H), 3.23–3.20 (m, 2H), 1.65–1.23 (m, 6H); HRMS (ESI) (*m/z*): calcd for C_22_H_29_FN_3_O_5_ 434.2086, found 434.2086 [M + H]^+^.

*(S)-N*-(6-(2,3-Dihydroxybenzamido)-1-hydroxyhexan-2-yl)nicotinamide (**9a**). General procedure **g** was followed with **8a** (330 mg, 0.83 mmol) and boron tribromide (1 m in DCM, 3.32 mL) to afford intermediate **9a** as a light-yellow powder (85.0 mg, 27%). ${\left[\alpha \right]}_{D}^{25}$ = −15.9 (c = 0.1, MeOH); ^1^H NMR (500 MHz, DMSO-*d*_6_): δ 12.40 (s, 1H), 9.17 (s, 1H), 8.99 (d, *J* = 2.2 Hz, 1H), 8.80 (t, *J* = 5.6 Hz, 1H), 8.68 (dd, *J* = 4.8, 1.7 Hz, 1H), 8.25 (d, *J* = 8.4 Hz, 1H), 8.16 (dt, *J* = 7.9, 2.0 Hz, 1H), 7.47 (dd, *J* = 8.0, 4.8 Hz, 1H), 7.24 (dd, *J* = 8.1, 1.5 Hz, 1H), 6.88 (dd, *J* = 7.8, 1.4 Hz, 1H), 6.63 (t, *J* = 7.9 Hz, 1H), 4.72 (s, 1H), 3.96 (dq, *J* = 9.3, 4.6 Hz, 1H), 3.47–3.44 (m, 1H), 3.39 (dd, *J* = 10.8, 6.0 Hz, 2H), 3.26 (s, 1H), 1.68–1.48 (m, 4H), 1.42–1.29 (m, 2H); ^13^C NMR (126 MHz, DMSO-*d*_6_): δ 169.7, 164.8, 151.6, 149.9, 148.5, 146.3, 135.0, 130.3, 123.3, 118.6, 117.7, 117.1, 114.9, 63.3, 51.5, 39.2, 30.3, 28.9, 23.2; HRMS (ESI) (*m/z*): calcd for C_19_H_24_N_3_O_5_ 374.1710, found 374.1711 [M + H]^+^.

(S)-N-(5-(2,3-Dihydroxybenzamido)-6-hydroxyhexyl)nicotinamide (**9b**). General procedure **g** was followed with **8b** (91.0 mg, 0.23 mmol) and boron tribromide (1 m in DCM, 0.920 mL) to afford intermediate **9b** as a light-yellow powder (12.0 mg, 14%). ${\left[\alpha \right]}_{D}^{25}$ = −19.6 (c = 0.1, MeOH); ^1^H NMR (500 MHz, DMSO-*d*_6_): δ 12.69 (s, 1H), 9.30 (s, 1H), 8.96 (d, *J* = 2.2 Hz, 1H), 8.67 (dd, *J* = 4.9, 1.7 Hz, 1H), 8.64 (t, *J* = 5.7 Hz, 1H), 8.37 (d, *J* = 8.4 Hz, 1H), 8.13 (dt, *J* = 8.0, 2.0 Hz, 1H), 7.46 (dd, *J* = 7.9, 4.8 Hz, 1H), 7.34 (dd, *J* = 8.1, 1.5 Hz, 1H), 6.89 (dd, *J* = 7.8, 1.4 Hz, 1H), 6.65 (t, *J* = 7.9 Hz, 1H), 4.77 (s, 1H), 3.99 (td, *J* = 9.1, 4.6 Hz, 1H), 3.51–3.46 (m, 1H), 3.44 (d, *J* = 7.0 Hz, 2H), 3.25 (s, 1H), 1.66–1.48 (m, 4H), 1.41–1.29 (m, 2H); ^13^C NMR (126 MHz, DMSO-*d*_6_): δ 169.5, 164.7, 151.7, 149.7, 148.3, 146.2, 134.9, 130.1, 123.4, 118.6, 117.6, 117.5, 115.2, 63.2, 51.0, 39.2, 30.2, 29.0, 23.2; HRMS (ESI) (*m/z*): calcd for C_19_H_24_N_3_O_5_ 374.1710, found 374.1710 [M + H]^+^.

*(S)-N*-(5-(2-Chlorobenzamido)-6-hydroxyhexyl)-2,3-dihydroxybenzamide (**9c**). General procedure **g** was followed with **8c** (90.0 mg, 0.21 mmol) and boron tribromide (1 m in DCM, 0.840 mL) to afford **9c** as a light-yellow powder (4.0 mg, 5%) and partially demethylated **9h** as a light-yellow powder (16.0 mg, 18%). ${\left[\alpha \right]}_{D}^{25}$ = −14.8 (c = 0.1, MeOH); ^1^H NMR (500 MHz, DMSO-*d*_6_): δ 12.91 (s, 1H), 9.10 (s, 1H), 8.82 (s, 1H), 8.10 (d, *J* = 8.6 Hz, 1H), 7.46 (dd, *J* = 8.0, 1.2 Hz, 1H), 7.41 (dd, *J* = 7.0, 2.0 Hz, 1H), 7.39–7.35 (m, 1H), 7.32 (td, *J* = 7.3, 1.3 Hz, 1H), 7.27 (dd, *J* = 8.2, 1.5 Hz, 1H), 6.89 (dd, *J* = 7.8, 1.5 Hz, 1H), 6.65 (t, *J* = 8.0 Hz, 1H), 4.69 (s, 1H), 3.87 (s, 1H), 3.45 (dd, *J* = 10.6, 5.3 Hz, 1H), 3.28 (d, *J* = 6.1 Hz, 3H), 1.68–1.46 (m, 4H), 1.37 (tt, *J* = 11.3, 6.1 Hz, 2H); ^13^C NMR (126 MHz, DMSO-*d*_6_): δ 169.7, 166.2, 150.0, 146.3, 137.5, 130.5, 129.8, 129.5, 128.8, 127.0, 118.6, 117.7, 117.1, 114.9, 63.5, 51.2, 39.0, 30.3, 28.9, 23.1; HRMS (ESI) (*m/z*): calcd for C_20_H_24_ClN_2_O_5_, 407.1368, found 407.1367 [M + H]^+^.

*(S)*-2,3-Dichloro-N-(6-(2,3-dihydroxybenzamido)-1-hydroxyhexan-2-yl)benzamide (**9d**). General procedure **g** was followed with **8d** (23.0 mg, 0.05 mmol) and boron tribromide (1 M in DCM, 0.200 mL) to afford **9d** as a light-yellow powder (4.0 mg, 18%). ${\left[\alpha \right]}_{D}^{25}$ = −13.6 (c = 0.1, MeOH); ^1^H NMR (500 MHz, DMSO-*d*_6_): δ 12.92 (s, 1H), 9.15 (s, 1H), 8.81 (t, *J* = 5.7 Hz, 1H), 8.25 (d, *J* = 8.6 Hz, 1H), 7.66 (dd, *J* = 7.6, 1.9 Hz, 1H), 7.38–7.30 (m, 2H), 7.28 (d, *J* = 8.0 Hz, 1H), 6.90 (d, *J* = 7.7 Hz, 1H), 6.67 (t, *J* = 7.9 Hz, 1H), 4.76 (s, 1H), 3.90–3.83 (m, 1H), 3.45 (d, *J* = 5.3 Hz, 1H), 3.29 (dq, *J* = 12.7, 6.7 Hz, 3H), 1.69–1.43 (m, 4H), 1.36 (dq, *J* = 15.9, 8.5 Hz, 2H); ^13^C NMR (126 MHz, DMSO-*d*_6_): δ 169.8, 165.6, 149.9, 146.3, 139.9, 132.0, 130.8, 128.4, 128.0, 127.3, 118.8, 117.9, 117.1, 114.9, 63.4, 51.3, 39.4, 30.3, 28.9, 23.2; HRMS (ESI) (*m/z*): calcd for C_20_H_23_Cl_2_N_2_O_5_ 441.0979, found 441.0981 [M + H]^+^.

*(S)*-*N*-(5-(2-Aminobenzamido)-6-hydroxyhexyl)-2,3-dihydroxybenzamide (**9e**). General procedure **g** was followed with **8e** (57.0 mg, 0.14 mmol) and boron tribromide (1 m in DCM, 0.560 mL) to afford **9e** as a light-yellow powder (14.0 mg, 26%). ${\left[\alpha \right]}_{D}^{25}$ = −11.2 (c = 0.1, MeOH); ^1^H NMR (500 MHz, DMSO-*d*_6_): δ 12.84 (s, 1H), 9.09 (s, 1H), 8.81 (t, *J* = 5.8 Hz, 1H), 7.77 (d, *J* = 8.4 Hz, 1H), 7.45 (dd, *J* = 8.0, 1.5 Hz, 1H), 7.25 (dd, *J* = 8.1, 1.5 Hz, 1H), 7.11 (ddd, *J* = 8.4, 7.0, 1.5 Hz, 1H), 6.88 (dd, *J* = 7.8, 1.4 Hz, 1H), 6.65 (t, *J* = 8.2 Hz, 2H), 6.51–6.45 (m, 1H), 6.27 (s, 2H), 4.75 (s, 1H), 3.93–3.88 (m, 1H), 3.41 (d, *J* = 5.5 Hz, 1H), 3.35–3.32 (m, 1H), 3.25 (q, *J* = 6.7 Hz, 2H), 1.65–1.43 (m, 4H), 1.40–1.25 (m, 2H); ^13^C NMR (126 MHz, DMSO-*d*_6_): δ 169.9, 169.0, 150.0, 149.5, 146.4, 128.6, 128.4, 118.9, 118.0, 117.2, 116.4, 115.6, 115.1, 114.8, 63.7, 50.9, 39.3, 30.5, 29.1, 23.4; HRMS (ESI) (*m/z*): calcd for C_20_H_26_N_3_O_5_ 388.1867, found 388.1864 [M + H]^+^.

*(S)*-2-Amino-3-chloro-N-(6-(2,3-dihydroxybenzamido)-1-hydroxyhexan-2-yl)benzamide (**9f**). General procedure **g** was followed with **8f** (57.0 mg, 0.13 mmol) and boron tribromide (1 m in DCM, 0.520 mL) to afford **9f** as a light-yellow powder (11.0 mg, 21%). ${\left[\alpha \right]}_{D}^{25}$ = −15.4 (c = 0.1, MeOH); ^1^H NMR (500 MHz, DMSO-*d*_6_): δ 12.91 (s, 1H), 9.13 (s, 1H), 8.79 (t, *J* = 5.7 Hz, 1H), 8.00 (d, *J* = 8.5 Hz, 1H), 7.49 (d, *J* = 8.1 Hz, 1H), 7.36 (d, *J* = 7.9 Hz, 1H), 7.26 (d, *J* = 7.1 Hz, 1H), 6.89 (d, *J* = 7.9 Hz, 1H), 6.66 (t, *J* = 7.9 Hz, 1H), 6.56 (t, *J* = 7.8 Hz, 1H), 6.39 (s, 2H), 4.73 (s, 1H), 3.92 (tq, *J* = 10.3, 5.5 Hz, 1H), 3.43 (t, *J* = 7.9 Hz, 2H), 3.26 (q, *J* = 6.7 Hz, 2H), 1.66–1.44 (m, 4H), 1.41–1.27 (m, 2H); ^13^C NMR (126 MHz, DMSO-*d*_6_): δ 169.8, 168.1, 149.8, 146.3, 144.9, 131.5, 127.4, 118.8, 118.8, 117.9, 117.7, 117.1, 115.2, 114.9, 63.4, 51.1, 39.0, 30.8, 28.9, 23.3; HRMS (ESI) (*m/z*): calcd for C_20_H_25_N_3_O_5_Cl 422.1477, found 422.1477 [M + H]^+^.

*(S)*-*N*-(5-(2-Amino-6-fluorobenzamido)-6-hydroxyhexyl)-2,3-dihydroxybenzamide (**9g**). General procedure **g** was followed with **8g** (84.0 mg, 0.19 mmol) and boron tribromide (1 M in DCM, 0.760 mL) to afford **9g** as a light-yellow powder (15.0 mg, 19%) and partially demethylated **9i** as a light-yellow powder (37.0 mg, 46%, purity >92%). ${\left[\alpha \right]}_{D}^{25}$ = −12.8 (c = 0.1, MeOH); ^1^H NMR (500 MHz, DMSO-*d*_6_): δ 12.85 (s, 1H), 9.05 (s, 1H), 8.80 (s, 1H), 7.85 (dd, *J* = 8.6, 2.4 Hz, 1H), 7.26 (dd, *J* = 8.2, 1.5 Hz, 1H), 7.07–7.03 (m, 1H), 6.89 (dd, *J* = 7.8, 1.5 Hz, 1H), 6.65 (t, *J* = 8.0 Hz, 1H), 6.49 (dd, *J* = 8.3, 1.0 Hz, 1H), 6.29 (ddd, *J* = 10.4, 8.1, 1.0 Hz, 1H), 5.78 (s, 2H), 4.73 (s, 1H), 3.92 (s, 1H), 3.42 (dd, *J* = 10.5, 5.7 Hz, 2H), 3.26 (d, *J* = 7.3 Hz, 2H), 1.66–1.48 (m, 4H), 1.41 (ddt, *J* = 12.9, 7.8, 4.0 Hz, 2H); ^13^C NMR (126 MHz, DMSO-*d*_6_): δ 169.7, 164.1, 160.2 (d, ^1^*J_CF_* = 247.5 Hz), 150.0, 149.2, 146.3, 130.7, 118.6, 117.7, 117.1, 114.9, 111.2, 108.3 (d, ^2^*J_CF_* = 19.3 Hz), 101.8 (d, ^2^*J_CF_* = 23.2 Hz), 63.3, 50.9, 40.4, 30.3, 28.9, 23.0; HRMS (ESI) (*m/z*): calcd for C_20_H_25_FN_3_O_5_ 406.1773, found 406.1773 [M + H]^+^.

*(S)*-*N*-(5-(2-Chlorobenzamido)-6-hydroxyhexyl)-2-hydroxy-3-methoxybenzamide (**9h**). Synthesis see **9c**.$\;{\left[\alpha \right]}_{D}^{25}$ = −19.6 (c = 0.1, MeOH); ^1^H NMR (500 MHz, DMSO-*d*_6_): δ 12.94 (s, 1H), 8.86 (t, *J* = 5.7 Hz, 1H), 8.15 (d, *J* = 8.6 Hz, 1H), 7.46–7.42 (m, 2H), 7.42–7.36 (m, 2H), 7.32 (td, *J* = 7.4, 1.3 Hz, 1H), 7.08 (dd, *J* = 8.0, 1.4 Hz, 1H), 6.80 (t, *J* = 8.1 Hz, 1H), 4.75 (s, 1H), 3.93–3.87 (m, 1H), 3.78 (s, 3H), 3.48 (dd, *J* = 10.6, 5.3 Hz, 1H), 3.32 (dq, *J* = 13.4, 6.7 Hz, 3H), 1.70–1.46 (m, 4H), 1.43–1.33 (m, 2H); ^13^C NMR (126 MHz, DMSO-*d*_6_): δ 169.6, 166.4, 151.2, 148.6, 137.6, 130.6, 129.9, 129.6, 128.9, 127.0, 118.6, 117.8, 115.3, 114.8, 63.6, 55.8, 48.7, 39.1, 30.4, 28.9, 23.2; HRMS (ESI) (*m/z*): calcd for C_21_H_26_ClN_2_O_5_ 421.1524, found 421.1525 [M + H]^+^.

*(S)*-*N*-(5-(2-Amino-6-fluorobenzamido)-6-hydroxyhexyl)-2-hydroxy-3-methoxybenzamide (**9i**). Synthesis see **9g**.$\;{\left[\alpha \right]}_{D}^{25}$ = −17.3 (c = 0.1, MeOH); ^1^H NMR (500 MHz, DMSO-*d*_6_): δ 12.92 (s, 1H), 8.83 (t, *J* = 5.7 Hz, 1H), 7.88 (d, *J* = 8.4 Hz, 1H), 7.41 (d, *J* = 8.2 Hz, 1H), 7.07 (ddd, *J* = 14.3, 8.1, 1.5 Hz, 2H), 6.79 (t, *J* = 8.1 Hz, 1H), 6.49 (d, *J* = 8.3 Hz, 1H), 6.32–6.25 (m, 1H), 5.79 (s, 2H), 4.77 (s, 1H), 3.93 (td, *J* = 9.0, 4.7 Hz, 1H), 3.77 (s, 3H), 3.42 (t, *J* = 8.4 Hz, 2H), 3.27 (q, *J* = 7.1 Hz, 2H), 1.65–1.50 (m, 3H), 1.39 (qt, *J* = 16.3, 10.3 Hz, 3H); ^13^C NMR (126 MHz, DMSO-*d*_6_): δ 169.6, 164.1, 160.3 (d, ^1^*J_CF_* = 240.2 Hz), 151.1, 149.5, 148.4, 130.9 (d, ^2^*J_CF_* = 11.2 Hz), 118.5, 117.7, 115.3, 114.8, 111.2 (d, ^3^*J_CF_* = 2.3 Hz), 108.5 (d, ^2^*J_CF_* = 19.3 Hz), 101.9, 63.4, 55.8, 51.0, 39.1, 30.3, 28.9, 23.1; HRMS (ESI) (*m/z*): calcd for C_21_H_27_FN_3_O_5_ 420.1929, found 420.1928 [M + H]^+^.

### 5.7. Reaction Monitoring and Purification of Synthetic Compounds

Reactions were monitored by a liquid chromatography-mass spectrometry (LC-MS) system equipped with Dionex UltiMate 3000 pump, autosampler, column compartment, detector, and ESI quadrupole MS (MSQ Plus or ISQ EC) from Thermo Fisher Scientific, Dreieich, Germany. Purification of the final products, when necessary, was performed using preparative HPLC (Dionex UltiMate 3000 UHPLC+ focused, Thermo Scientific) on a reversed-phase column (C18 column, 5 μm, MACHEREY-NAGEL, Düren, Germany). The solvents used for the chromatography were water with 0.05% formic acid and acetonitrile with 0.05% formic acid. Unless indicated otherwise, reagents and substrates were purchased from commercial sources and used as received. Solvents not required to be dry were purchased as technical grade and used as received. Dry solvents were purchased from commercial sources in Sure/Seal^TM^ bottles and used as received and stored under a dry inert gas (N_2_ or Ar). Inert atmosphere experiments were performed with standard Schlenk techniques with dried (P_2_O_5_) nitrogen gas. All reported compounds were characterized by ^1^H- and ^13^C-NMR and compared with literature data. All final products were fully characterized by ^1^H- and ^13^C-NMR and HRMS techniques. The purity of the final products and intermediates was determined by LC-MS and found to be >95%, unless otherwise stated.

### 5.8. Bioactivity

#### 5.8.1. Assessment of Antimicrobial Activities (MIC and Synergy)

All microorganisms used in this study were obtained from the German Collection of Microorganisms and Cell Cultures (DSMZ), the Coli Genetic Stock Center (CGSC), or were part of our internal collection, and were handled according to standard procedures. Single colonies of *S. aureus* and *E. coli* were inoculated from CASO agar plates into cation-adjusted Mueller Hinton Broth to obtain a final inoculum of 10^5^ colony-forming units (CFU)/mL. Single colonies of *C. albicans* on Sabouraud agar plates and spores of *M. hiemalis* were used to prepare the inoculums in Myc2 medium (10 g/L Bacto-peptone, 10 g/L yeast extract, 0.2% glycerol, pH 6.3). The tested derivatives were prepared as DMSO stocks (10 mg/mL). Serial dilutions of derivatives in the respective growth medium (0.06 to 128 μg/mL) were prepared in sterile 96-well plates and the bacterial or fungal suspensions were added. Growth inhibition was assessed after incubation at 37 °C (*S. aureus*, *E. coli*) and 30 °C (*C. albicans*, *M. hiemalis*), respectively. Minimum inhibitory concentrations (MIC) are defined as the lowest compound concentration where no visible growth is observed. The checkerboard assay was used to evaluate synergism, antagonism, or indifference between the tested derivative **9a** and a number of antibiotics (daptomycin, gentamicin, linezolid, and ciprofloxacin). Briefly, 96-well microtiter plates were prepared: panel A was prepared for a serial dilution of the antibiotics and panel B was used for the serial dilution of the tested derivative. Then, 50 µL was transferred from wells of panel A and dispensed in the corresponding wells of panel B. Bacterial suspension of *S. aureus* str. Newman were added to the plate to achieve approximately 10^5^ CFU/mL. The plates were incubated at 37 °C for 24 h. The MICs of panel A and panel B alone as well as the combination wells were determined.

#### 5.8.2. Cytotoxic Activity (IC_50_)

HepG2 cells (human hepatoblastoma cell line; ACC 180, DSMZ) and HCT-116 cells (human colon carcinoma cell line; ACC 581, DSMZ) were cultured under conditions recommended by the depositor and cells were propagated in Dulbecco’s modified Eagle medium (DMEM) and McCoy’s 5A medium, respectively, supplemented with 10% fetal bovine serum (FBS). For determining the antiproliferative activity of test compounds, cells were seeded at 6 × 10^3^ cells per well of 96-well plates in 120 μL complete medium. After 2 h of equilibration, compounds were added in serial dilution in 60 µL complete medium. Compounds, as well as the solvent control and doxorubicin as reference, were tested as duplicates in two independent experiments. After 5 d incubation, 20 μL of 5 mg/mL MTT (thiazolyl blue tetrazolium bromide) in PBS was added per well and cells were further incubated for 2 h at 37 °C. The medium was then discarded and cells were washed with 100 μL PBS before adding 100 μL 2-propanol/10 n HCl (250:1) in order to dissolve formazan granules. The absorbance at 570 nm was measured using a microplate reader (Tecan Infinite M200Pro), and cell viability was expressed as percentage relative to the respective solvent control. IC_50_ were determined by sigmoidal curve fitting using GraphPad PRISM 8 (GraphPad Software, San Diego, CA, USA).

## Figures and Tables

**Table 1:** NMR data of **1** measured in MeOH-*d*_4_ at 500 (^1^H) and 125 (^13^C) MHz.

	1 (in CD_3_OD)		
No.	δ_H_, Mult. (*J* in Hz)	δ_C_, Type	HMBC
1	3.63, brd (3.4)	64.4, CH_2_	2, 3
	3.61, brd (3.8)		
2	4.15, m	53.2, CH	4, 7′
3	1.63, m	30.2, CH_2_	1, 2, 4, 5
4	1.49, m	22.6, CH_2_	2, 5, 6
5	1.72, m	28.9, CH_2_	3, 6
6	3.39, t (7.0)	39.4, CH_2_	4, 5, 7″
2′	8.65, brd (8.2)	151.0, CH	3′, 4′, 6′
3′	7.49, dd (8.1, 5.0)	123.6, CH	2′, 5′
4′	8.20, brd (7.7)	135.7, CH	2′, 3′, 6′
5′		130.9, C	
6′	8.96, brd (2.2)	147.7, CH	2′, 4′, 7′
7′		166.7, C	
1″		115.3, C	
2″		148.6, C	
3″		145.9, C	
4″	6.90, d (8.0)	118.1, CH	2″, 3″, 6″
5″	6.67, t (8.0)	118.1, CH	1″, 3″
6″	7.16, d (8.1)	117.1, CH	1″, 2″, 4″, 7″
7″		170.1, C	

**Table 2:** NMR data of **3** measured in MeOH-*d*_4_ at 500 (^1^H) and 125 (^13^C) MHz.

	3 (in CD_3_OD)		
No.	δ_H_, Mult. (*J* in Hz)	δ_C_, Type	HMBC
1	3.62, dd (10.7, 5.4)	64.8, CH_2_	2, 3
	3.64, dd (10.6, 5.2)		
2	4.15, m	53.7, CH	4, 7′
3	1.64, m	30.2, CH_2_	1, 2, 4, 5
4	1.49, m	23.4, CH_2_	2, 5, 6
5	1.74, m	29.2, CH_2_	3, 6
6	3.42, m	40.4, CH_2_	4, 5, 7″
2′/2″	8.66, o	151.7, CH *	4′, 6′
3′/3″	7.51, dd (7.9, 2.3)	123.6, CH	5′
4′/4″	8.20, o	136.4, CH	2′, 6′, 7′
5′/5″		131.2, C *	
6′/6″	8.95, o	148.2, CH	4′, 7′
7′/7″		167.2, C	

* Data extracted from HSQC and HMBC spectra. o Overlapping signals.

**Table 3:** Synthetic analogues provided for biological activity testing.

	R^1^	R^2^
**9a** = **1**	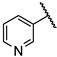	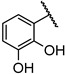
**9b** = **2**	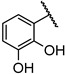	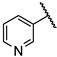
**9c** [12]	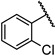	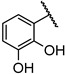
**9d**	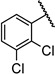	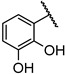
**9e**	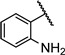	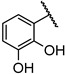
**9f**	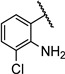	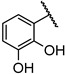
**9g**	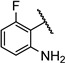	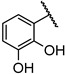
**9h**	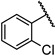	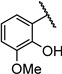
**9i**	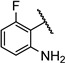	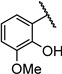

**Table 4:** Antiproliferative activity of synthetic myxochelin derivatives against HCT-116 and HepG2 cell lines. Half-inhibitory concentrations (IC_50_) were determined as duplicates and are given as average values from two independent experiments ± SD.

Compound	IC_50_ [µg/mL]	
	HCT-116	HepG2
**9a**	>37	>37
**9b**	29.1 ± 3.0	>37
**9c**	6.1 ± 0.1	6.7 ± 0.9
**9d**	29.5 ± 9.2	35.9 ± 0.9
**9e**	17.4 ± 8.0	11.3 ± 0.7
**9f**	6.7 ± 2.0	10.6 ± 2.6
**9g**	9.2 ± 2.3	11.8 ± 1.7
**9h**	>37	>37
**9i**	>37	>37
Doxorubicin	0.2	0.2

## Data Availability

Compound data for **1**, **2** and **3** was deposited to npatlas.org.

## Supplementary Materials

The following are available online, Figure S1. UV spectrum of **1** (natural product) in water/acetonitrile mixture with 0.1% formic acid, Figure S2. UV spectrum of **2** (natural product) in water/acetonitrile mixture with 0.1% formic acid, Figure S3. UV spectrum of **3** (natural product) in water/acetonitrile mixture with 0.1% formic acid, Figure S4. UV spectrum of **9a** = **1** (synthetic compound) in water/acetonitrile mixture with 0.1% formic acid, Figure S5. UV spectrum of **9b** = **2** (synthetic compound) in water/acetonitrile mixture with 0.1% formic acid, Figure S6. UV spectrum of **9c** in water/acetonitrile mixture with 0.1% formic acid, Figure S7. UV spectrum of **9d** in water/acetonitrile mixture with 0.1% formic acid, Figure S8. UV spectrum of **9e** in water/acetonitrile mixture with 0.1% formic acid, Figure S9. UV spectrum of **9f** in water/acetonitrile mixture with 0.1% formic acid, Figure S10. UV spectrum of **9g** in water/acetonitrile mixture with 0.1% formic acid, Figure S11. UV spectrum of **9h** in water/acetonitrile mixture with 0.1% formic acid, Figure S12. UV spectrum of **9i** in water/acetonitrile mixture with 0.1% formic acid, Figure S13. Tandem MS spectrum of myxochelin A, Figure S14. Tandem MS spectrum of **1**, Figure S15. Tandem MS spectrum of **2**, Figure S16. Tandem MS spectrum of **3**, Figure S17. Tandem MS spectrum of **9a = 1**, Figure S18. Tandem MS spectrum of **9b** = **2**, Figure S19. Tandem MS spectrum of **9c**, Figure S20. Tandem MS spectrum of **9d**, Figure S21. Tandem MS spectrum of **9e**, Figure S22. Tandem MS spectrum of **9f**, Figure S23. Tandem MS spectrum of **9g**, Figure S24. Tandem MS spectrum of **9h**, Figure S25. Tandem MS spectrum of **9i**, Figure S26. CD spectrum of 1 mg/mL **1** (isolated) in methanol in the area 180–450 nm, Figure S27. CD spectrum of 1 mg/mL **9a** = **1** (synthetic) in methanol in the area 180–450 nm, Figure S28. CD spectrum of 1 mg/mL **2** (isolated) in methanol in the area 180–450 nm, Figure S29. CD spectrum of 1 mg/mL **9b** = **2** (synthetic) in methanol in the area 180–450 nm, Figure S30. HPLC-MS base peak chromatogram (BPC) of *Corallococcus* sp. MCy9049 in VY/2S medium with highlighted extracted ion chromatograms (EICs) of 374.17105 *m/z* (green: **1, 2**), 343.17647 *m/z* (blue, **3**) and 405.16565 (orange: myxochelin A), with a width of 0.005 *m/z*, Figure S31. HPLC-MS base peak chromatogram (BPC) of *Corallococcus* sp. MCy9049 in VY/2S medium with supplementation of 1 mM nicotinamide with highlighted extracted ion chromatograms (EICs) of 374.17105 *m/z* (green: **1, 2**), 343.17647 *m/z* (blue, **3**) and 405.16565 (orange: myxochelin A), with a width of 0.005 *m/z*, Figure S32. HPLC-MS base peak chromatogram (BPC) of *Corallococcus* sp. MCy9049 in VY/2S medium with supplementation of 1 mM nicotinic acid with highlighted extracted ion chromatograms (EICs) of 374.17105 *m/z* (green: **1, 2**), 343.17647 *m/z* (blue, **3**) and 405.16565 (orange: myxochelin A), with a width of 0.005 *m/z*, Figure S33. HPLC-MS extracted ion chromatograms (EICs) of 374.17105 *m/z* (green: **1, 2**), 343.17647 *m/z* (blue, **3**) and 405.16565 (orange: myxochelin A), with a width of 0.005 *m/z* from crude extracts of *Corallococcus* sp. MCy9049 in VY/2S medium with supplementation of ferric sodium EDTA, Figure S34. HPLC-MS extracted ion chromatograms (EICs) of 374.17105 *m/z* (green: **1, 2**) and 343.17647 *m/z* (blue, **3**), with a width of 0.005 m/z from crude extracts of *Corallococcus* sp. MCy9049 in VY/2S medium with supplementation of different pyridinecarboxylic acids, Figure S35. HPLC-MS UV chromatogram and positive ESI-MS of **9a**, Figure S36. HPLC-MS UV chromatogram and positive ESI-MS of **9b**, Figure S37. HPLC-MS UV chromatogram and positive ESI-MS of **9c**, Figure S38. HPLC-MS UV chromatogram and positive ESI-MS of **9d**, Figure S39. HPLC-MS UV chromatogram and positive ESI-MS of **9e**, Figure S40. HPLC-MS UV chromatogram and positive ESI-MS of **9f**, Figure S41. HPLC-MS UV chromatogram and positive ESI-MS of **9g**, Figure S42. HPLC-MS UV chromatogram and positive ESI-MS of **9h**, Figure S43. HPLC-MS UV chromatogram and positive-ESI MS of **9i**, Figure S44. ^1^H NMR spectrum of **1** in methanol-d_4_, Figure S45. ^1^H-^1^H COSY spectrum of **1** in methanol-d_4_, Figure S46. ^13^C NMR spectrum of **1** in methanol-d_4_, Figure S47. HSQC spectrum of **1** in methanol-d_4_, Figure S48. HMBC spectrum of **1** in methanol-d_4_, Figure S49. ^1^H NMR spectrum of **3** in methanol-d_4_, Figure S50. ^1^H-^1^H COSY spectrum of **3** in methanol-d_4_, Figure S51. HSQC spectrum of **3** in methanol-d_4_, Figure S52. HMBC spectrum of **3** in methanol-d_4_, Figure S53. ^1^H NMR spectrum of **9a** in DMSO-d_6_, Figure S54. 13C NMR spectrum of **9a** in DMSO-d6, Figure S55. ^1^H NMR spectrum of **9b** in DMSO-d_6_, Figure S56. ^13^C NMR spectrum of **9b** in DMSO-d_6_, Figure S57. ^1^H NMR spectrum of **9c** in DMSO-d_6_, Figure S58. ^13^C NMR spectrum of **9c** in DMSO-d_6_, Figure S59. ^1^H NMR spectrum of **9d** in DMSO-d_6_, Figure S60. ^13^C NMR spectrum of **9d** in DMSO-d_6_, Figure S61. ^1^H NMR spectrum of **9e** in DMSO-d_6_, Figure S62. ^13^C NMR spectrum of **9e** in DMSO-d_6_, Figure S63. ^1^H NMR spectrum of **9f** in DMSO-d_6_, Figure S64. ^13^C NMR spectrum of **9f** in DMSO-d_6_, Figure S65. ^1^H NMR spectrum of **9g** in DMSO-d_6_, Figure S66. ^13^C NMR spectrum of **9g** in DMSO-d_6_, Figure S67. ^1^H NMR spectrum of **9h** in DMSO-d_6_, Figure S68. ^13^C NMR spectrum of **9h** in DMSO-d_6_, Figure S69. ^1^H NMR spectrum of **9i** in DMSO-d_6_, Figure S70. ^13^C NMR spectrum of **9i** in DMSO-d_6_, Figure S71. Alternative producers of **1–3** (**A**–**C**) and myxochelin A (**D**) from our in-house metabolome database sorted by suborder. Search parameters: exact mass deviation <5 ppm, retention time deviation <0.3 min, area threshold 3 × 10^4^. The decreasing number of hits from **1** to **3** is most likely due to the threshold of the peak finding algorithm, Figure S72. Venn Diagrams of alternative producers of **1**–**3** (**A**–**C**) and of Myxochelin A from our in-house metabolome database. Search parameters: exact mass deviation <5 ppm, retention time deviation <0.3 min, area threshold 3 × 10^4^, Figure S73. Consensus sequences for 8 angstrom signature code (upper) and Stachelhaus code (lower) of myxobacterial *mxcE* translations, Table S1. Tabulated blastP results for the CDS regions present in the MCy9049 myxochelin biosynthetic gene cluster, Table S2. A-domain specificity codes for *mxcE* homologs from NRPSpredictor2. Prediction for all hits: dihydroxybenzoic acid or salicylic acid, Table S3. Minimum inhibitory concentration (MICs) for the synthetic myxochelins against a panel of microorganisms and MICs of reference drugs in combination with **9a**.

## Sample Availability

Samples of the synthetic compounds are available from the authors.
