# Supplementary material for: Expanding the Myxochelin Natural Product Family by Nicotinic Acid Containing Congeners
Source: Molecules. 2021 Aug 14;26(16):4929. doi: 10.3390/molecules26164929 (PMC8400222; doi:10.3390/molecules26164929)
Supplement: Supplementary file 1 [file molecules-26-04929-s001.zip › molecules-1318445-supplementary.pdf]

Expanding the myxochelin natural product family by nicotinic acid containing congeners

Nicolas A. Frank [1,2,†], Márió Széles [1,2,†], Sergi H. Akone [1,2,3], Sari Rasheed [1,2], Stephan Hüttel [1,2], Simon Frewert [1,2], Mostafa M. Hamed [1,2], Jennifer Herrmann [1,2], Sören M. M. Schuler [4], Anna K. H. Hirsch [1,2] and Rolf Müller [1,2,*]

1 Helmholtz-Institute for Pharmaceutical Research Saarland (HIPS), Helmholtz Centre for Infection Research (HZI) and Department of Pharmacy, Saarland University, Campus E8 1, 66123 Saarbrücken, Germany

2 German Center for Infection Research (DZIF), Partner Site Hannover-Braunschweig, 38124 Braunschweig, Germany

3 Department of Chemistry, Faculty of Science, University of Douala, P.O.Box 24157 Douala, Cameroon

4 Evotec International GmbH, 37079 Göttingen, Germany

† These authors contributed equally

Supplementary Information

Table of Contents

1 CHARACTERIZATION OF MYXOCHELIN DERIVATIVES 3

1.1 UV SPECTRA OF THE MYXOCHELINS 3

1.1.1 UV spectra of naturally occurring myxochelins 3

1.1.2 UV spectra of synthetic myxochelins 4

1.2 TANDEM MS SPECTRA 7

1.2.1 Tandem MS spectra of naturally occurring myxochelins 7

1.2.1 Tandem MS spectra of synthetic myxochelins 8

1.3 CIRCULAR DICHROISM SPECTRA 10

1.4 HPLC-MS CHROMATOGRAMS OF CRUDE EXTRACTS 12

1.5 HPLC-MS CHROMATOGRAMS OF THE SYNTHETIC COMPOUNDS 15

1.6 SYNTHESIS OF ARYL SUBSTITUENTS WITH AN FMOC-PROTECTED AMINO GROUP 23

1.7 NMR-BASED STRUCTURE ELUCIDATION 25

1.7.1 ^{1}H and ^{13}C-spectra of compounds 1 and 3 25

1.7.2 ^{1}H and ^{13}C spectra of the synthetic compounds 30

2 *IN-SILICO* ANALYSIS OF THE MYXOCHELIN N1–N3 BIOSYNTHESIS 48

2.1 METABOLOME DATABASE SEARCH OF 1–3 48

2.2 ANALYSIS OF THE MYXOCHELIN BGC 49

3 ASSESSMENT OF BIOLOGICAL ACTIVITIES 51

4 REFERENCES 52

1 Characterization of Myxochelin derivatives

1.1 UV spectra of the myxochelins

1.1.1 UV spectra of naturally occurring myxochelins

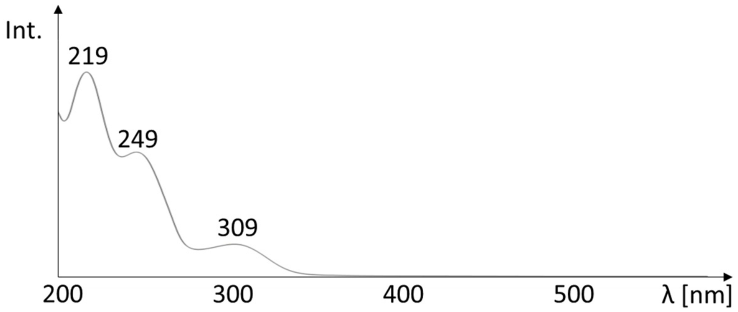

*Figure S1. UV spectrum of **1**(natural product) in water/acetonitrile mixture with 0.1% formic acid*

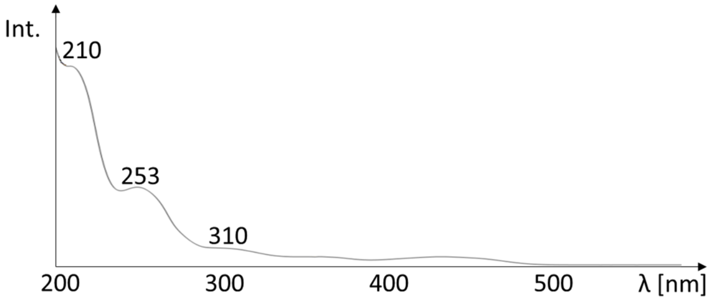

*Figure S2. UV spectrum of **2** (natural product) in water/acetonitrile mixture with 0.1% formic acid*

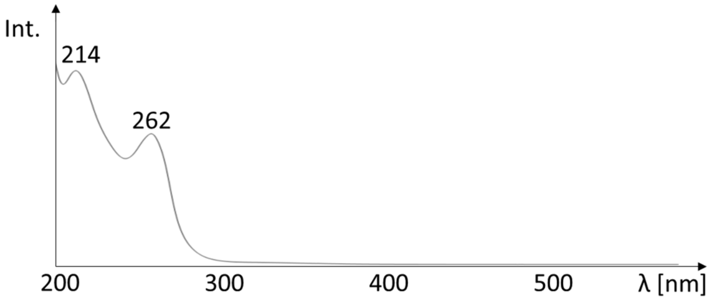

*Figure S3. UV spectrum of **3** (natural product) in water/acetonitrile mixture with 0.1% formic acid*

1.1.2 UV spectra of synthetic myxochelins

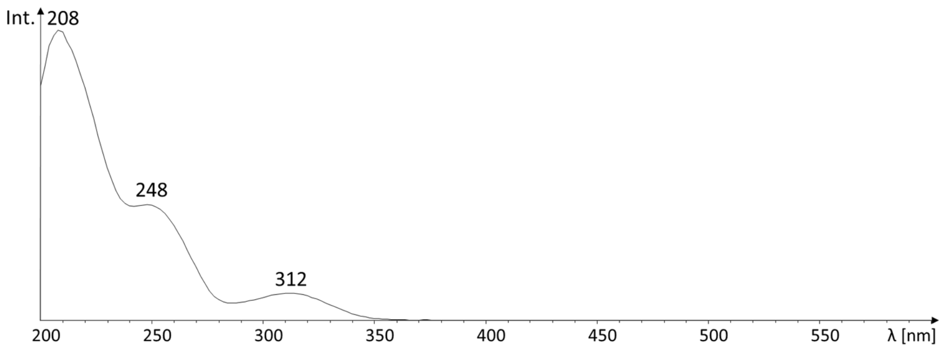

*Figure S4. UV spectrum of **9a = 1** (synthetic compound) in water/acetonitrile mixture with 0.1% formic acid*

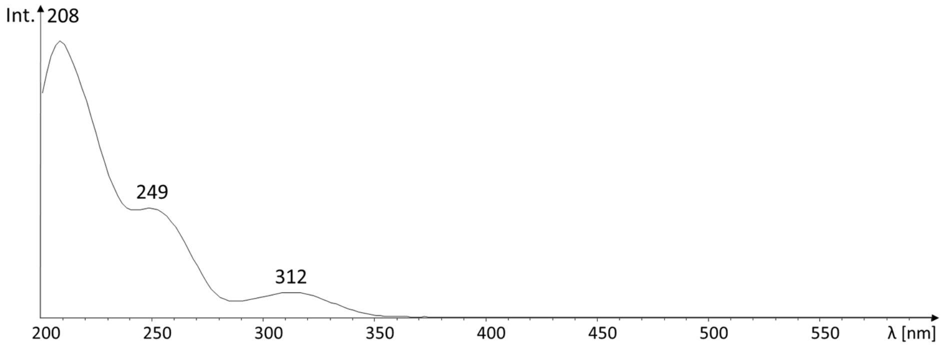

*Figure S5. UV spectrum of **9b = 2** (synthetic compound) in water/acetonitrile mixture with 0.1% formic acid*

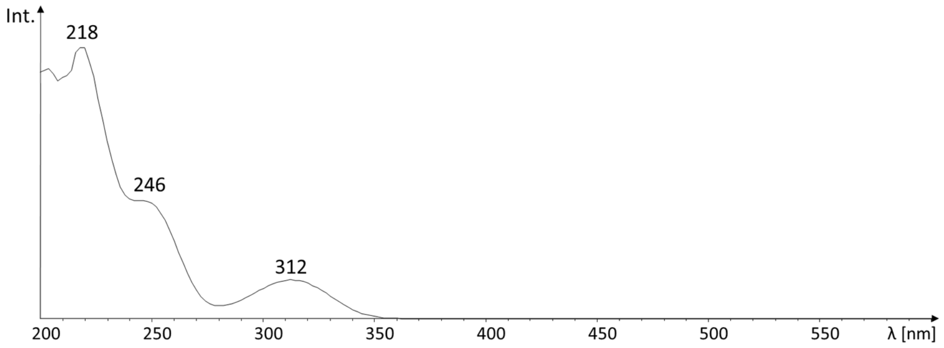

*Figure S6. UV spectrum of **9c** in water/acetonitrile mixture with 0.1% formic acid*

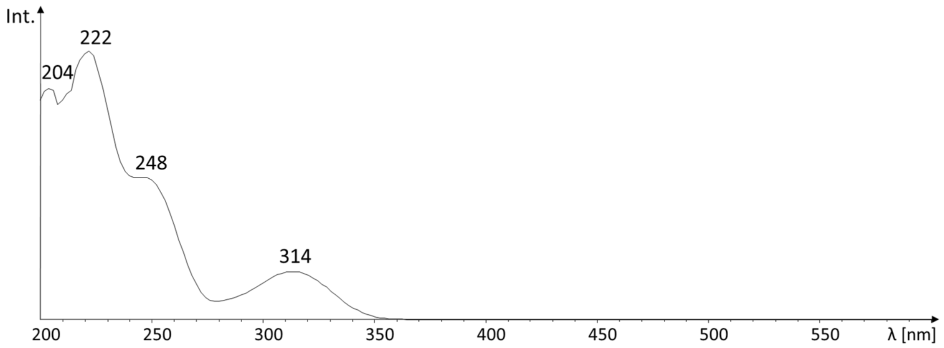

*Figure S7. UV spectrum of **9d** in water/acetonitrile mixture with 0.1% formic acid*

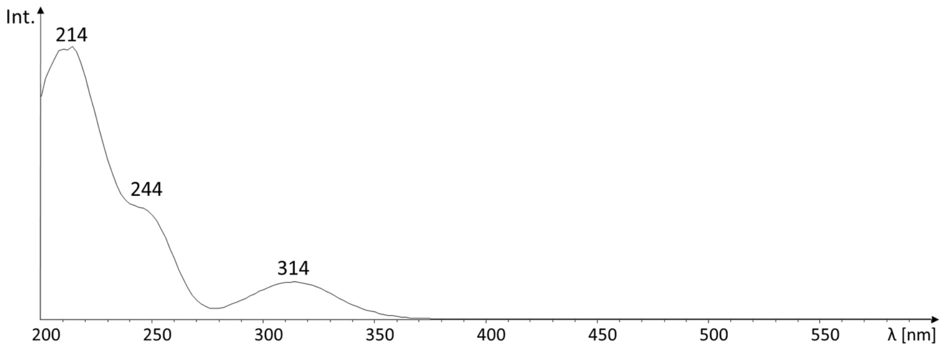

*Figure S8. UV spectrum of **9e** in water/acetonitrile mixture with 0.1% formic acid*

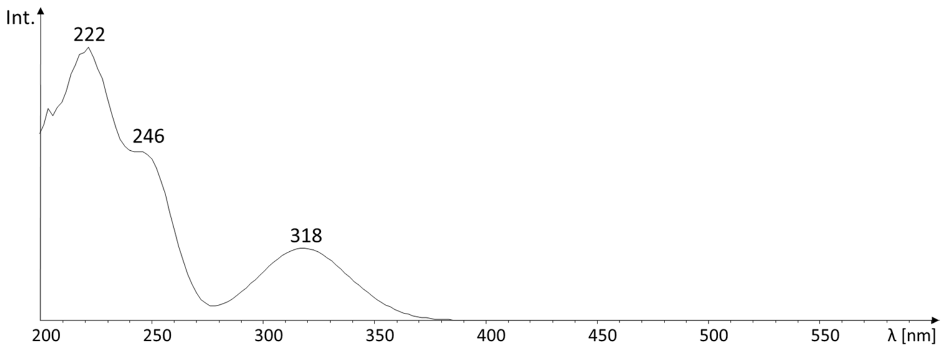

*Figure S9. UV spectrum of **9f** in water/acetonitrile mixture with 0.1% formic acid*

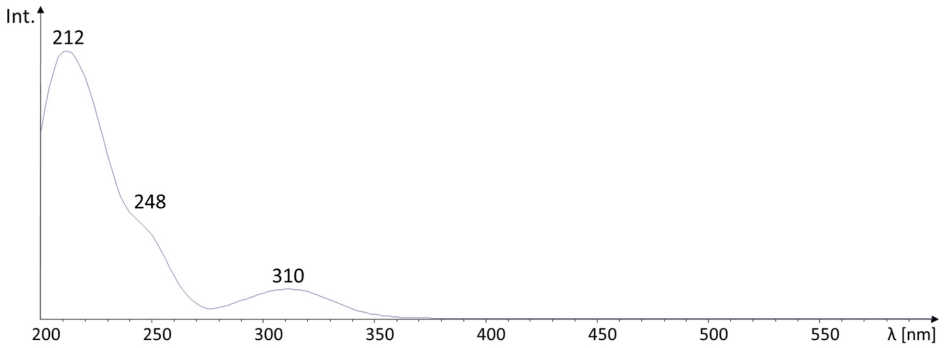

*Figure S10. UV spectrum of **9g** in water/acetonitrile mixture with 0.1% formic acid*

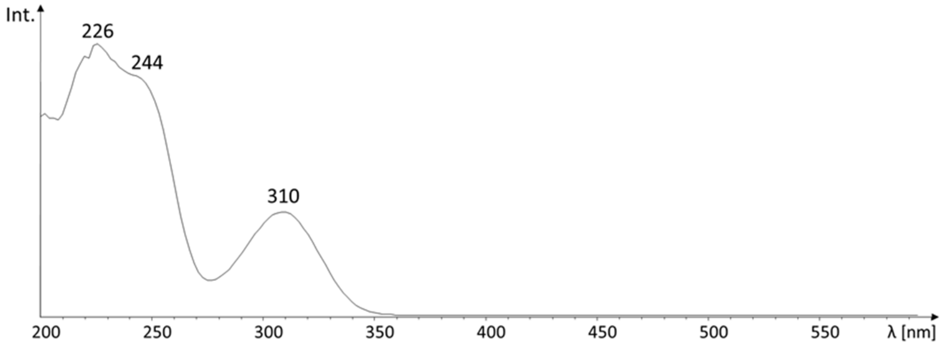

*Figure S11. UV spectrum of **9h** in water/acetonitrile mixture with 0.1% formic acid*

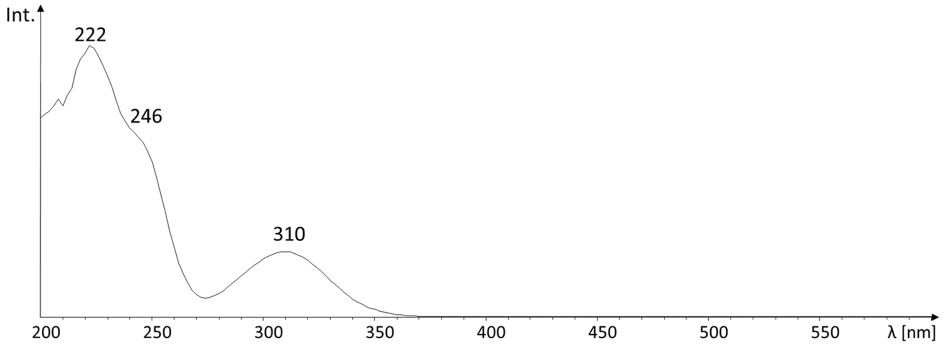

*Figure S12. UV spectrum of **9i** in water/acetonitrile mixture with 0.1% formic acid*

1.2 Tandem MS spectra

1.2.1 Tandem MS spectra of naturally occurring myxochelins

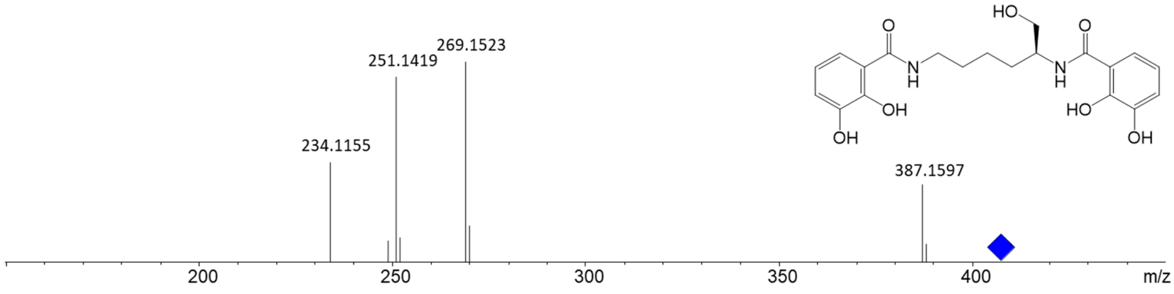

Figure S13. Tandem MS spectrum of myxochelin A

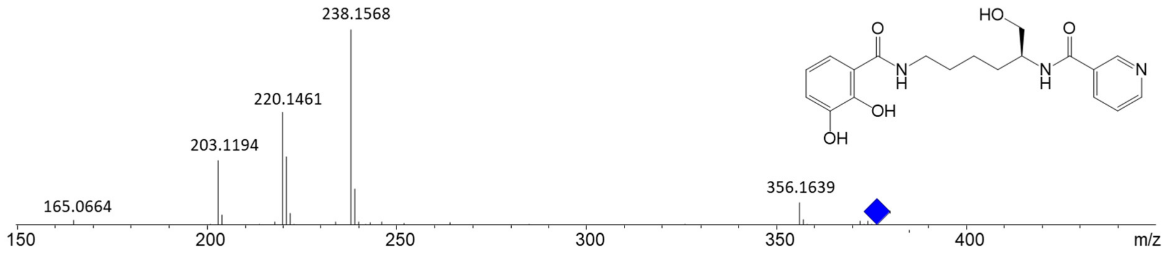

*Figure S14. Tandem MS spectrum of **1***

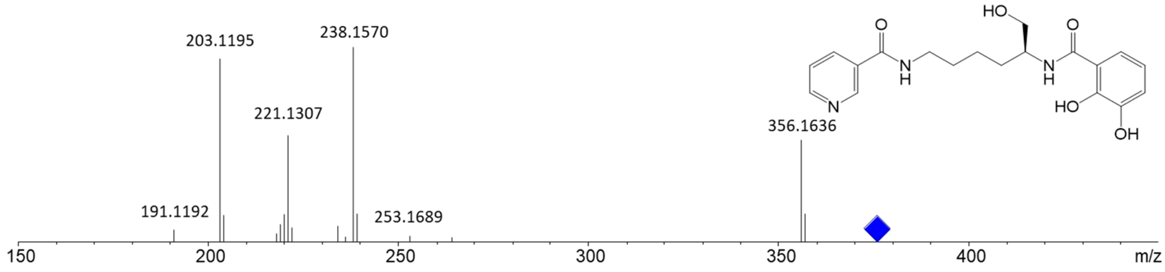

*Figure S15. Tandem MS spectrum of **2***

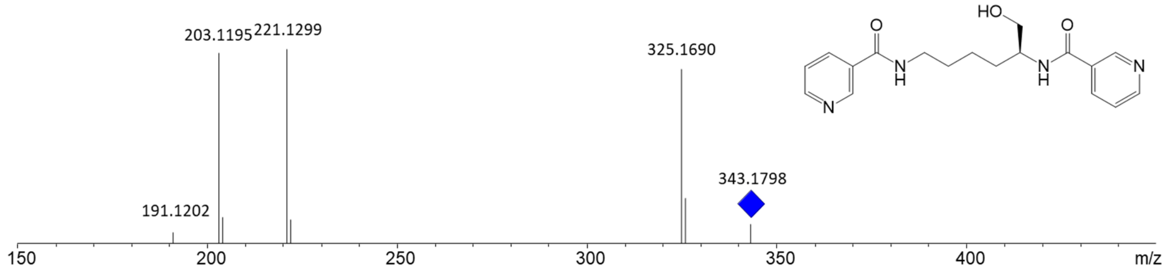

*Figure S16. Tandem MS spectrum of **3***

1.2.1 Tandem MS spectra of synthetic myxochelins

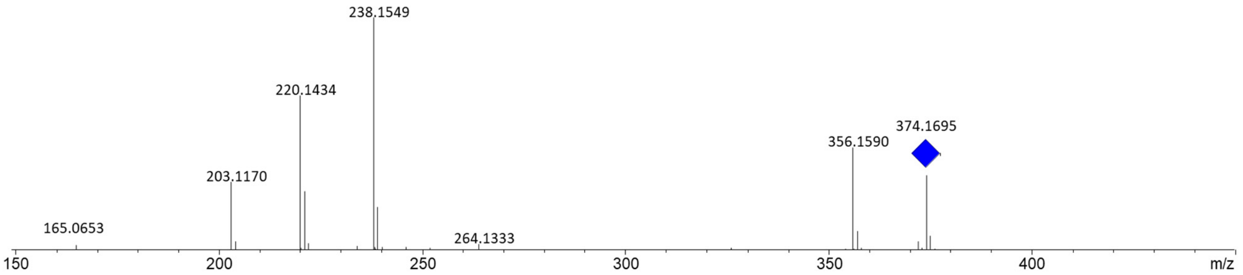

*Figure S17. Tandem MS spectrum of **9a = 1***

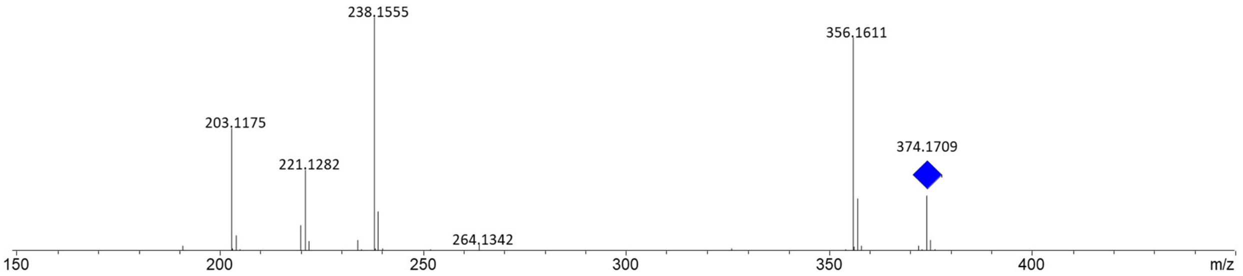

*Figure S18. Tandem MS spectrum of **9b = 2***

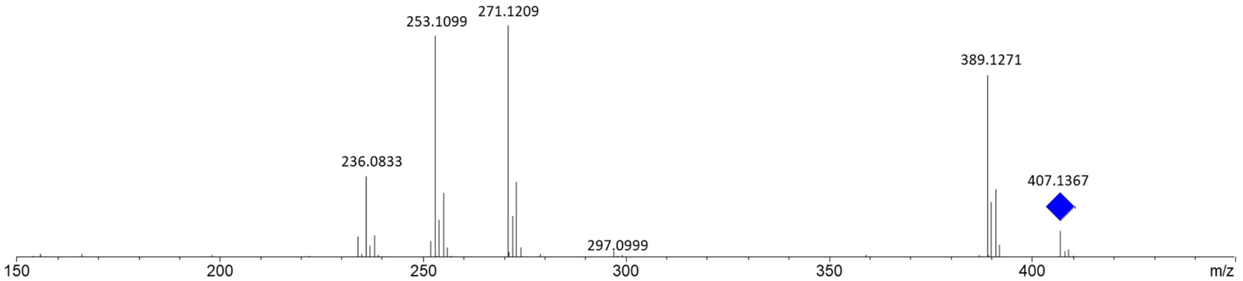

*Figure S19. Tandem MS spectrum of **9c***

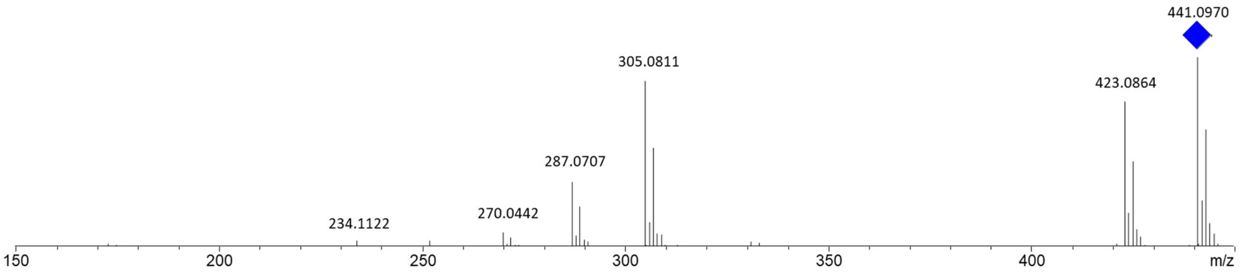

*Figure S20. Tandem MS spectrum of **9d***

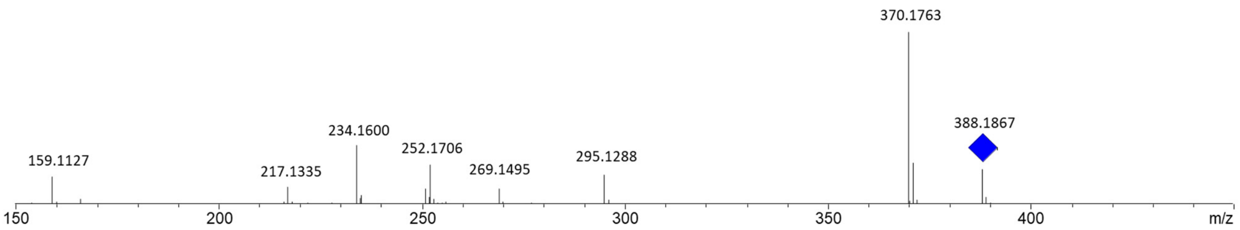

*Figure S21. Tandem MS spectrum of **9e***

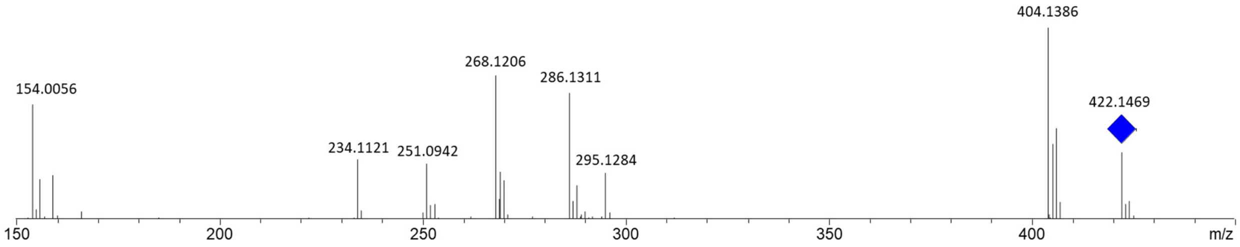

*Figure S22. Tandem MS spectrum of **9f***

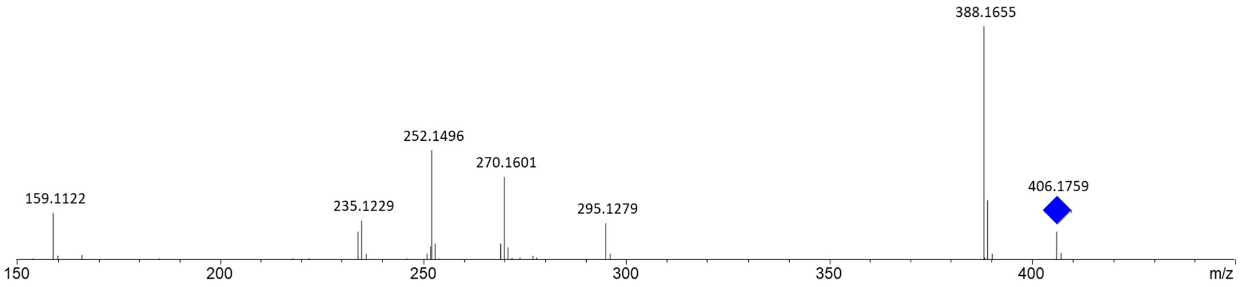

*Figure S23. Tandem MS spectrum of **9g***

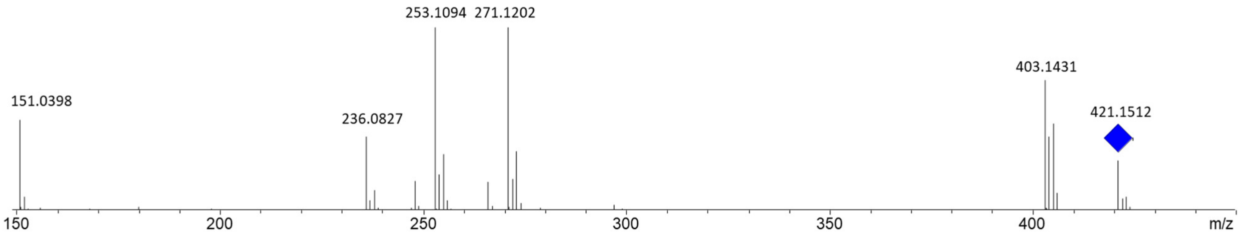

*Figure S24. Tandem MS spectrum of **9h***

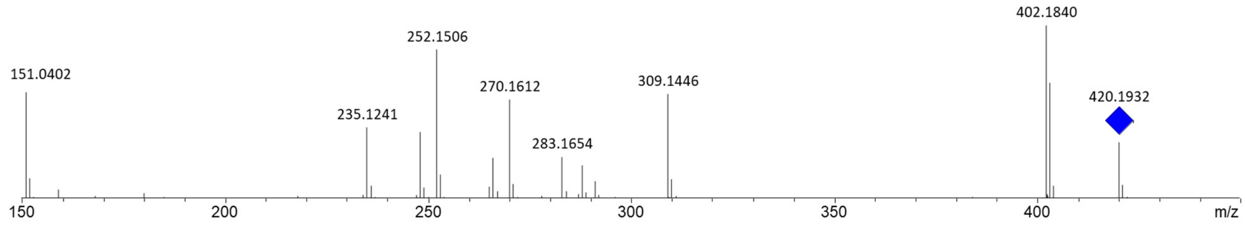

*Figure S25. Tandem MS spectrum of **9i***

1.3 Circular dichroism spectra

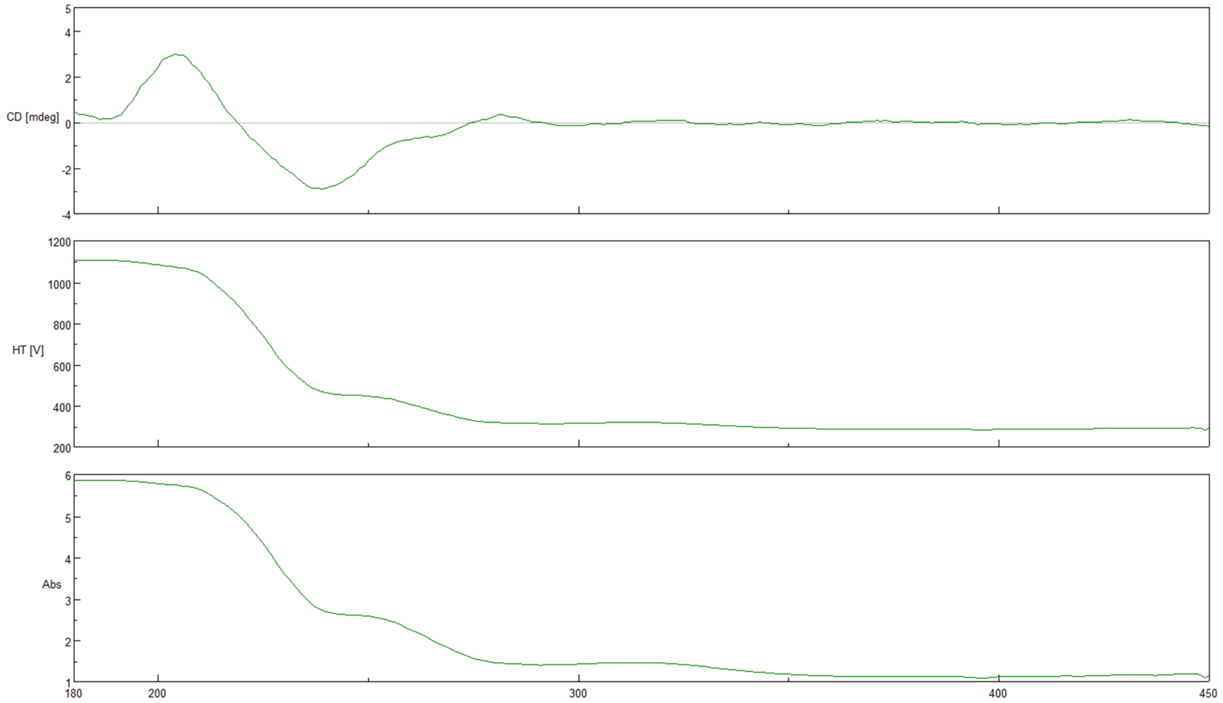

*Figure S26. CD spectrum of 1mg/mL **1** (isolated) in methanol in the area 180–450 nm*

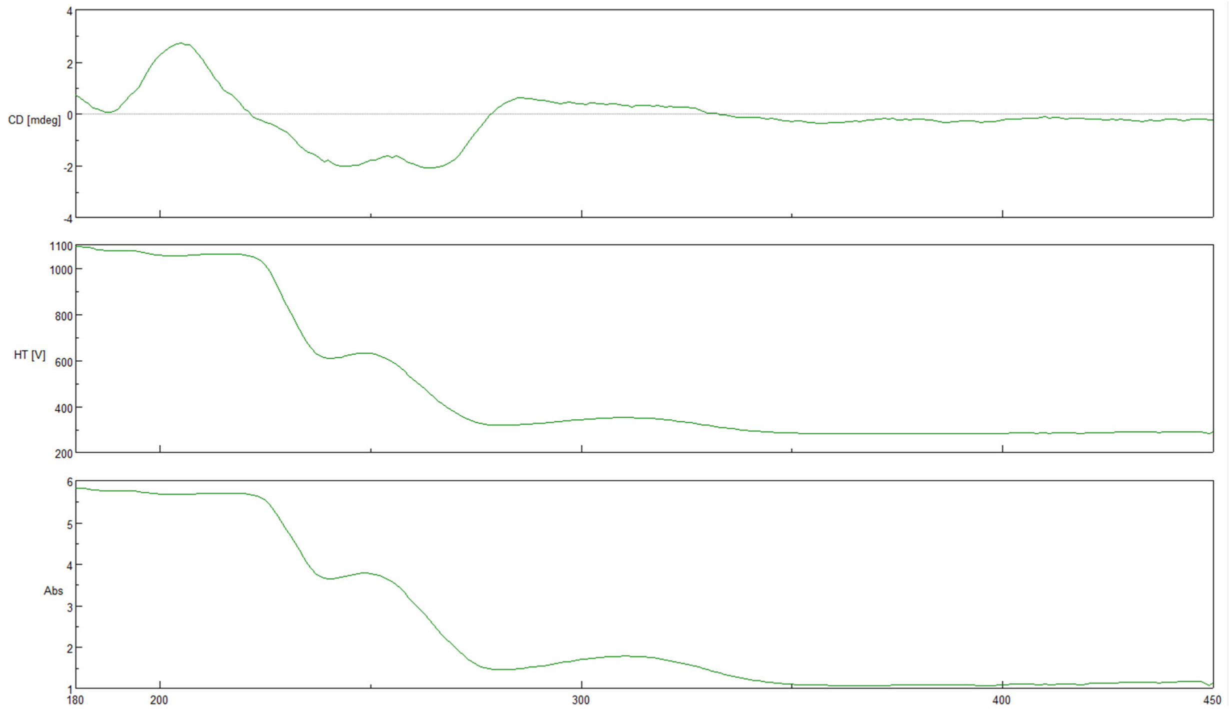

Figure S27. CD spectrum of 1mg/mL ***9a* = *1*** *(synthetic) in methanol in the area 180–450 nm*

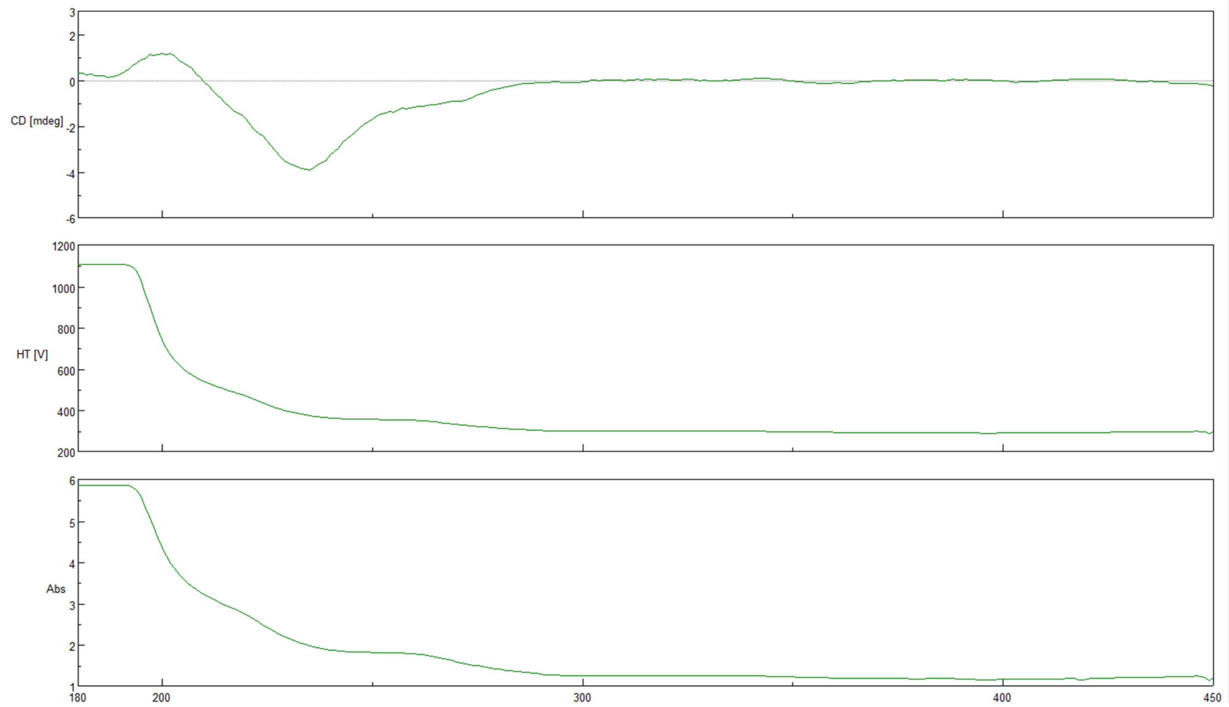

Figure S28. CD spectrum of 1mg/mL ***2*** *(isolated) in methanol in the area 180–450 nm*

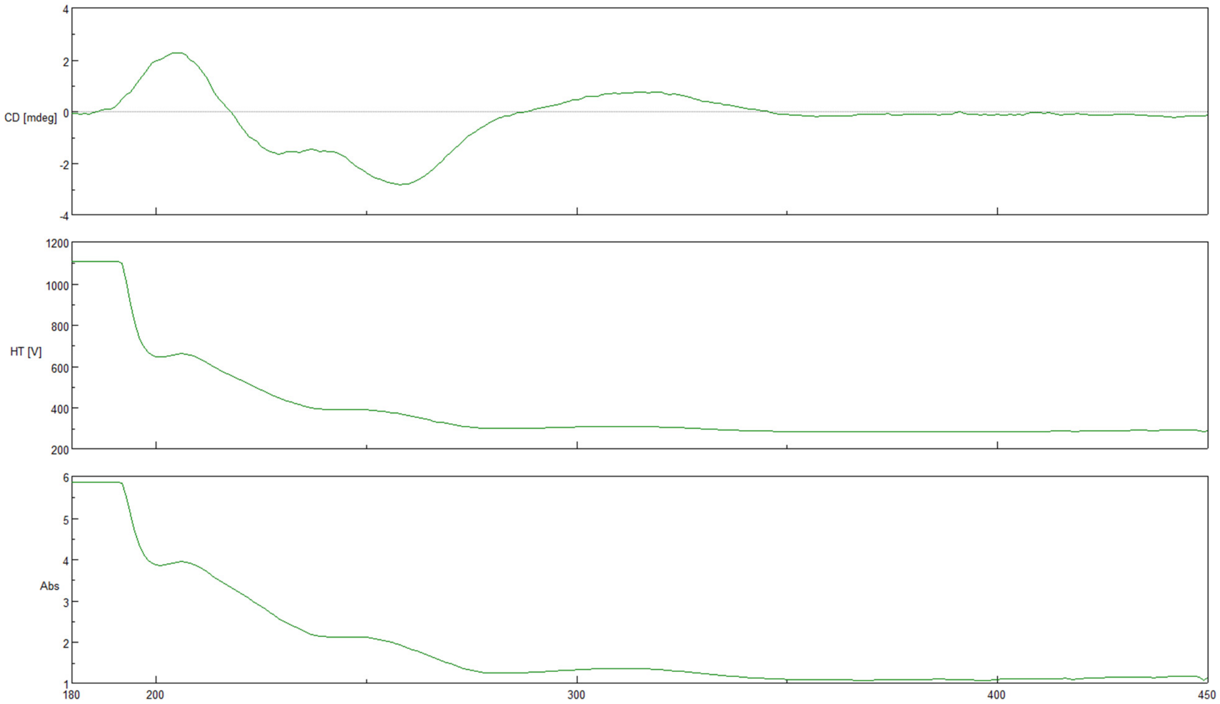

*Figure S29. CD spectrum of 1mg/mL **9b = 2** (synthetic) in methanol in the area 180–450 nm*

1.4 HPLC-MS chromatograms of crude extracts

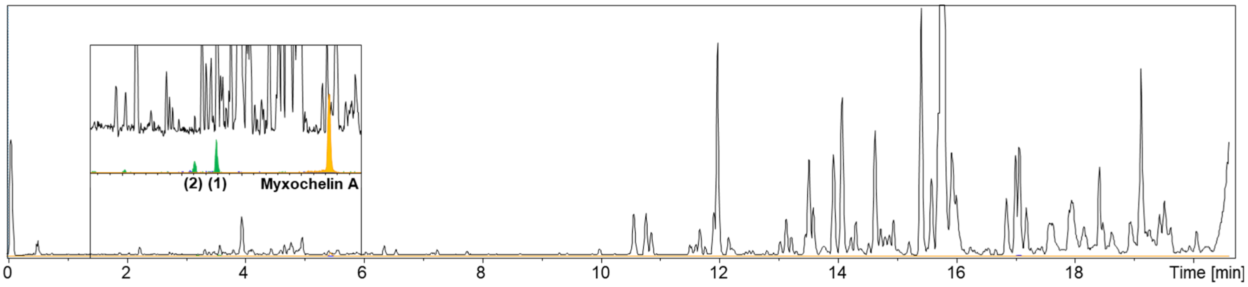

Figure S30. HPLC-MS base peak chromatogram (BPC) of Corallococcus *sp. MCy9049 in VY/2S medium with highlighted extracted ion chromatograms (EICs) of 374.17105* m/z *(green: **1, 2**), 343.17647* m/z *(blue, **3**) and 405.16565 (orange: myxochelin A), with a width of 0.005* m/z.

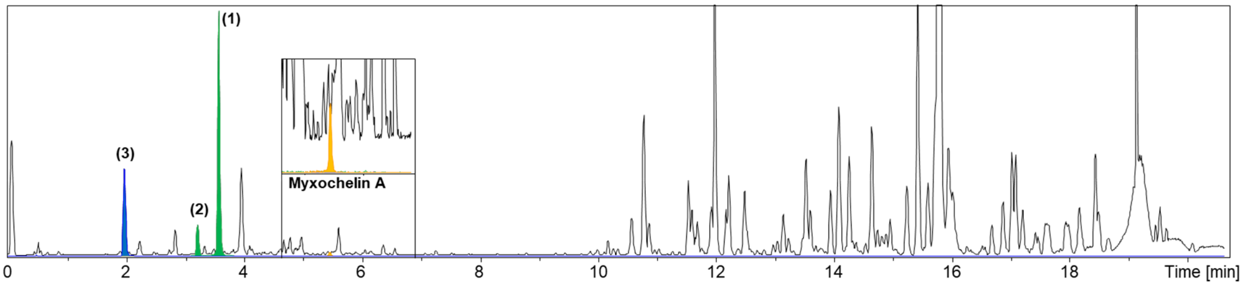

Figure S31. HPLC-MS base peak chromatogram (BPC) of Corallococcus *sp. MCy9049 in VY/2S medium with supplementation of 1 mM nicotinamide with highlighted extracted ion chromatograms (EICs) of 374.17105* m/z *(green:* ***1, 2****), 343.17647* m/z *(blue,* ***3****) and 405.16565 (orange: myxochelin A), with a width of 0.005* m/z.

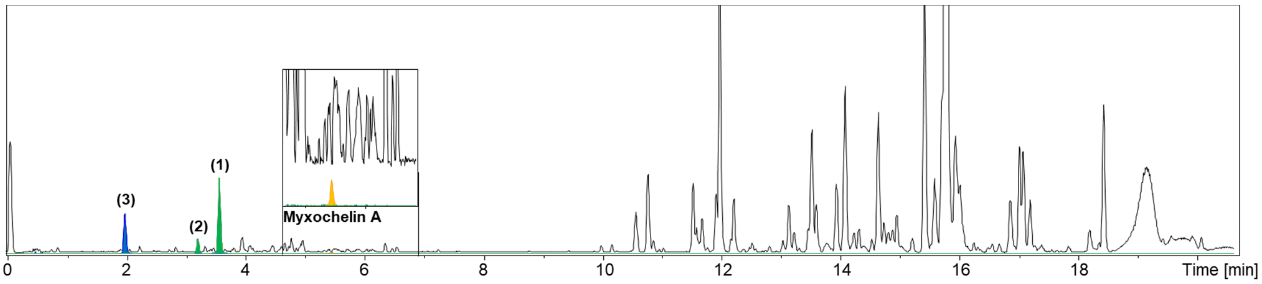

Figure S32. HPLC-MS base peak chromatogram (BPC) of Corallococcus *sp. MCy9049 in VY/2S medium with supplementation of 1 mM nicotinic acid with highlighted extracted ion chromatograms (EICs) of 374.17105* m/z *(green:* ***1, 2****), 343.17647* m/z *(blue,* ***3****) and 405.16565 (orange: myxochelin A), with a width of 0.005* m/z.

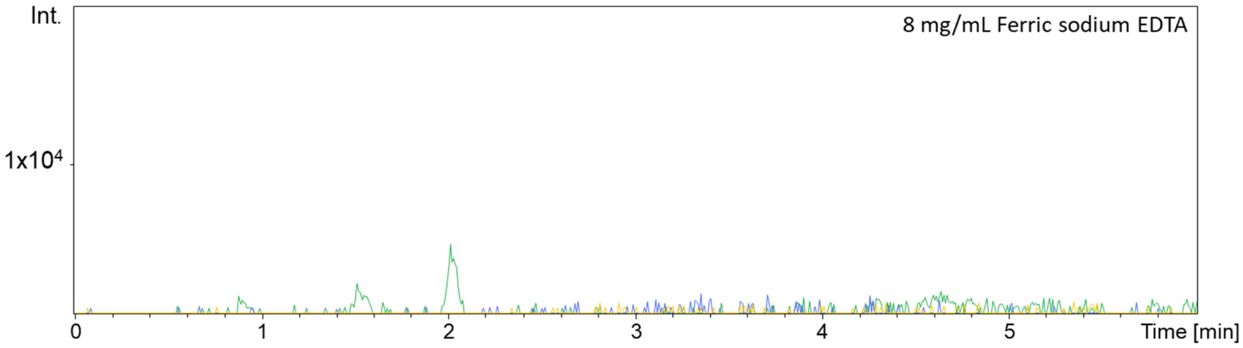

Figure S33. HPLC-MS extracted ion chromatograms (EICs) of 374.17105 m/z *(green:* ***1, 2****), 343.17647* m/z *(blue,* ***3****) and 405.16565 (orange: myxochelin A), with a width of 0.005* m/z *from crude extracts of* Corallococcus *sp. MCy9049 in VY/2S medium with supplementation of ferric sodium EDTA.*

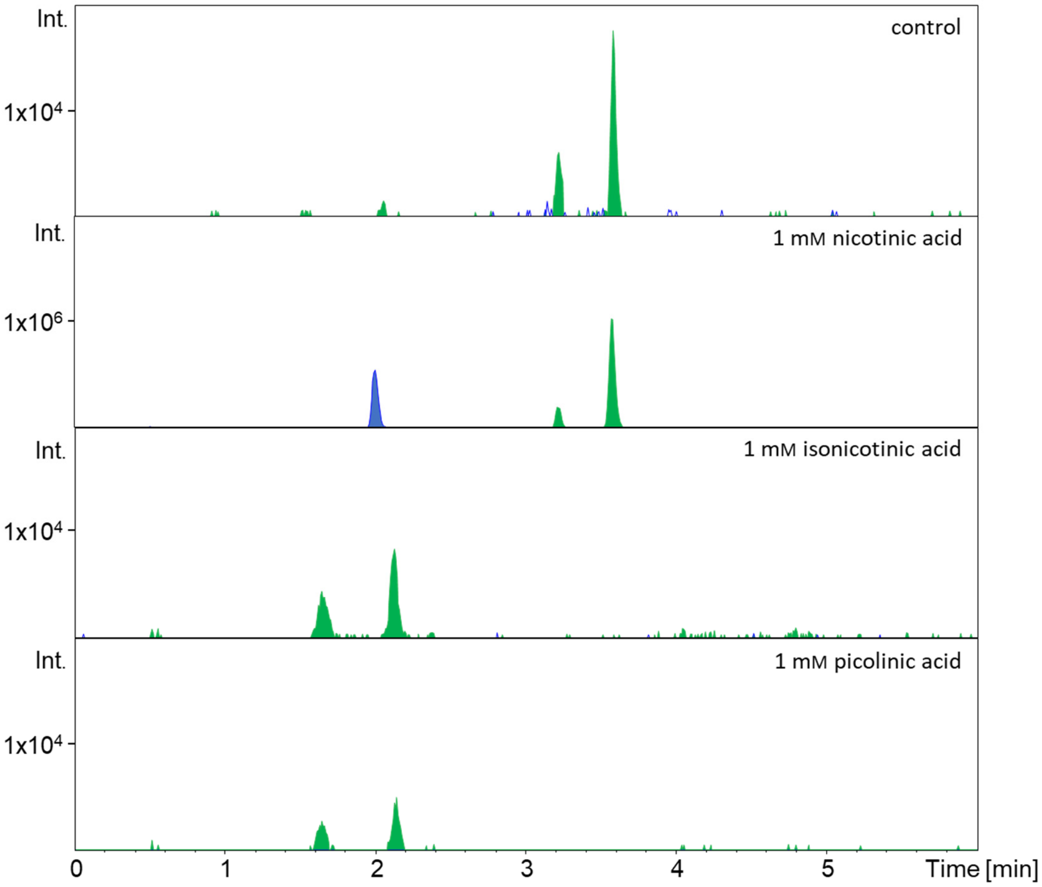

Figure S34. HPLC-MS extracted ion chromatograms (EICs) of 374.17105 m/z *(green:* ***1, 2****) and 343.17647* m/z *(blue,* ***3****), with a width of 0.005* m/z *from crude extracts of* Corallococcus sp. *MCy9049 in VY/2S medium with supplementation of different pyridinecarboxylic acids.*

1.5 HPLC-MS chromatograms of the synthetic compounds

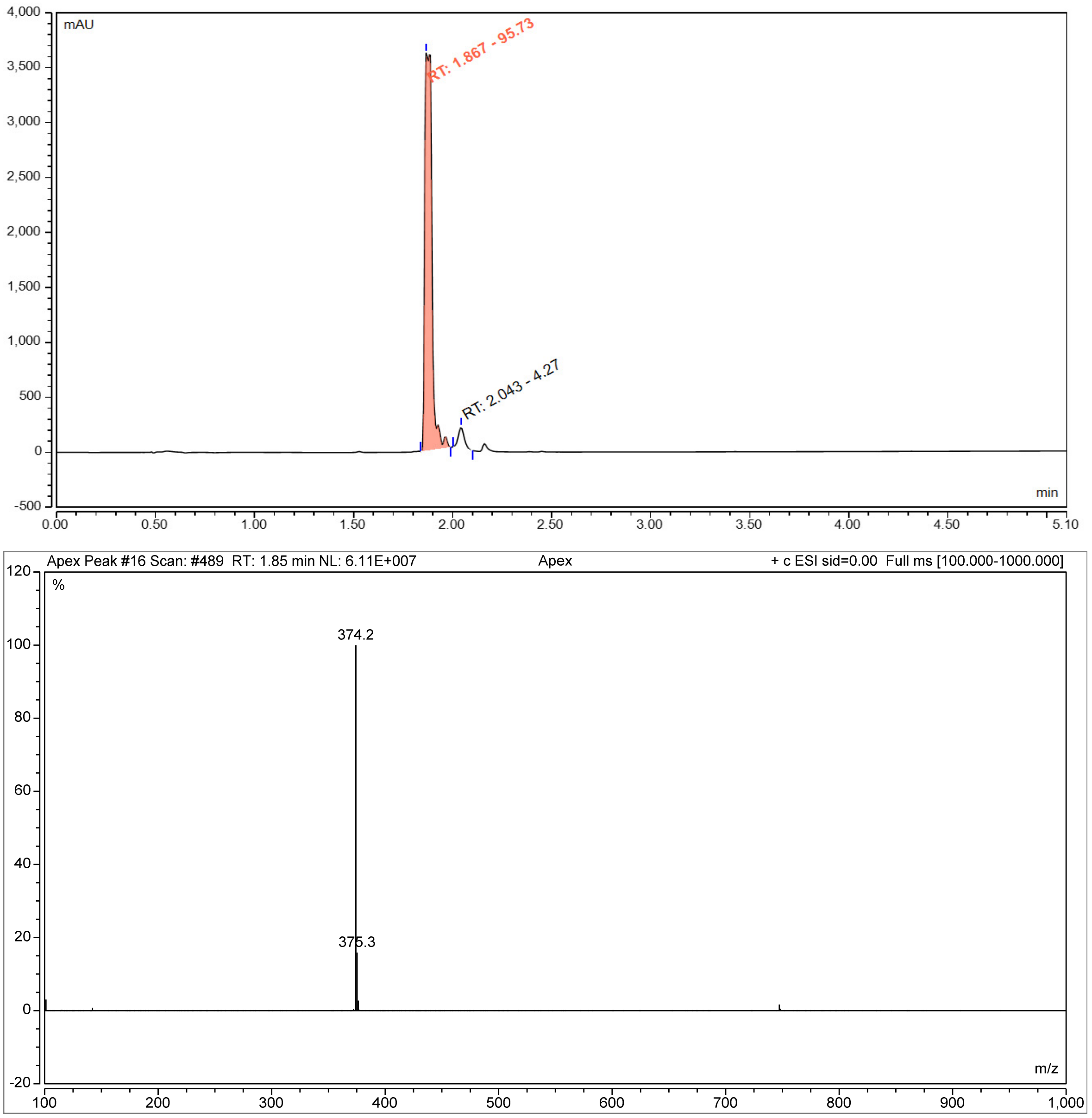

*Figure S35. HPLC-MS UV chromatogram and positive ESI-MS of **9a**.*

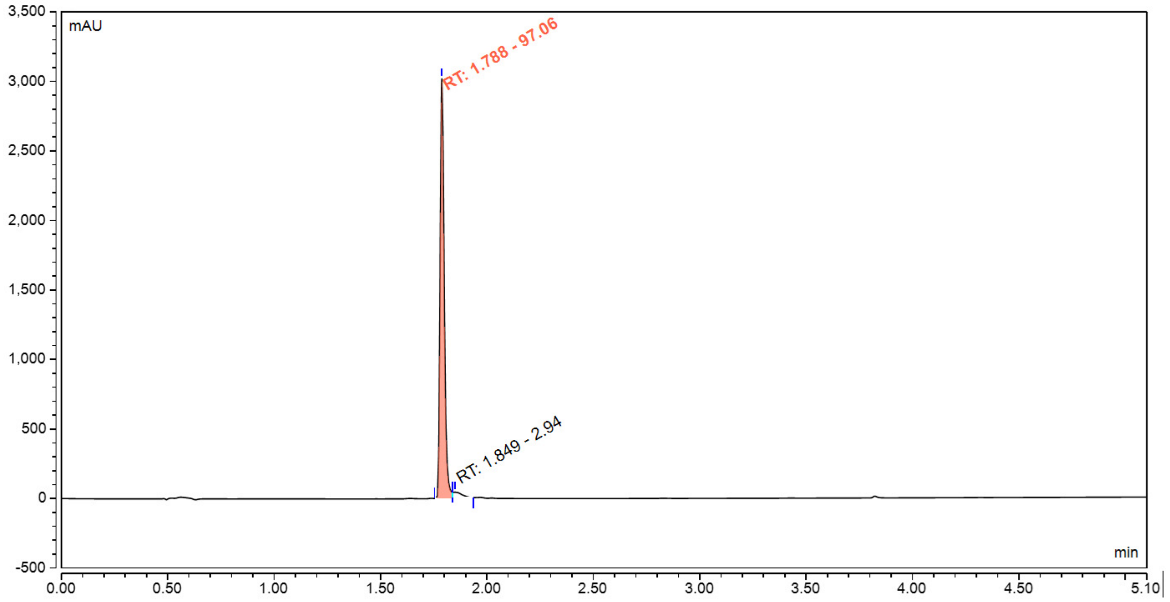

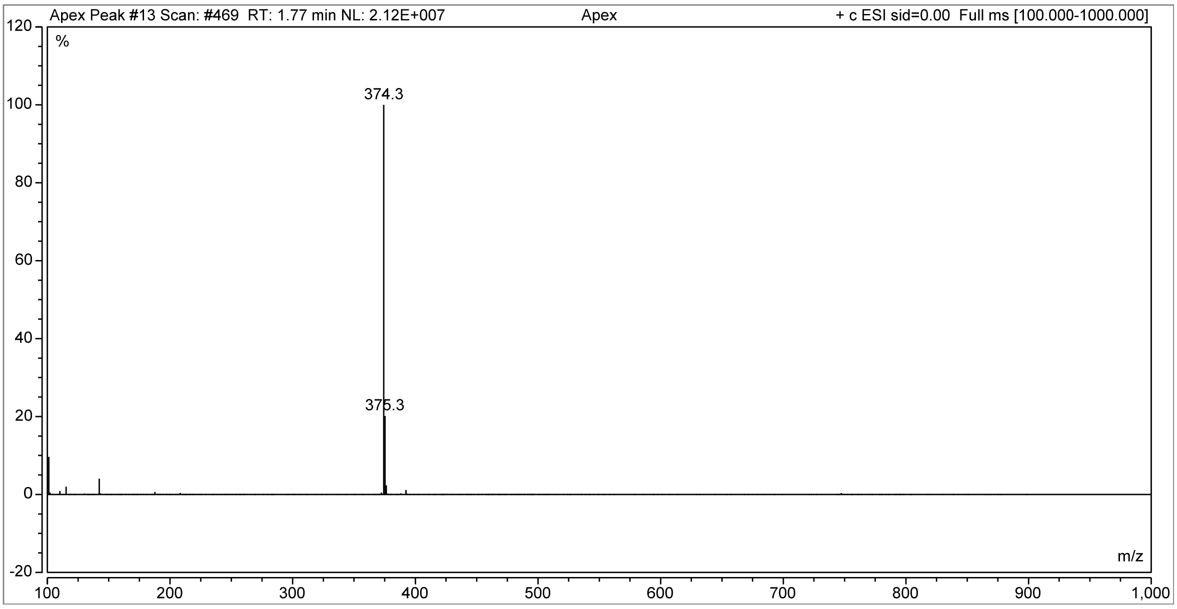

*Figure S36. HPLC-MS UV chromatogram and positive ESI-MS of **9b**.*

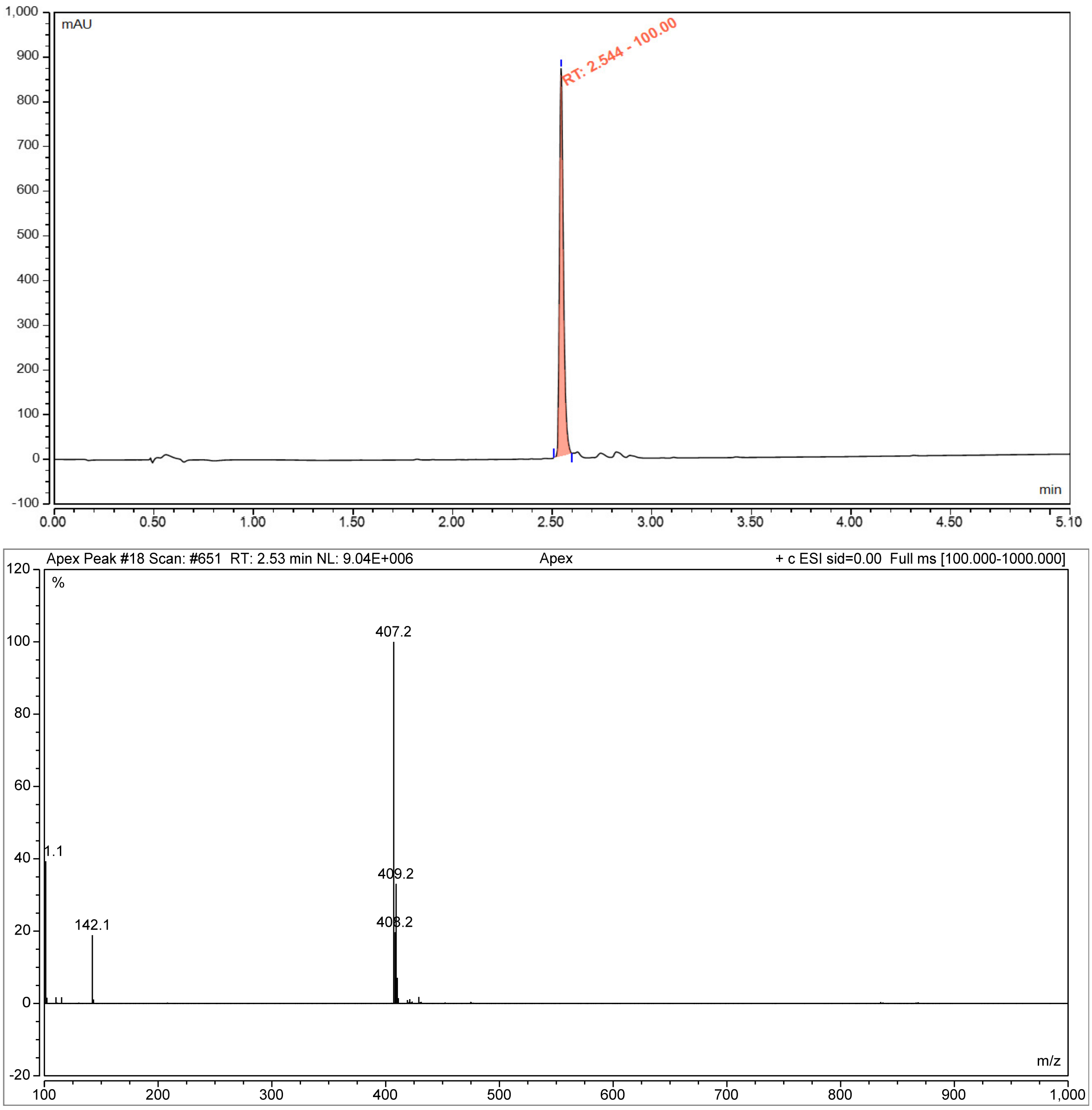

*Figure S37. HPLC-MS UV chromatogram and positive ESI-MS of **9c**.*

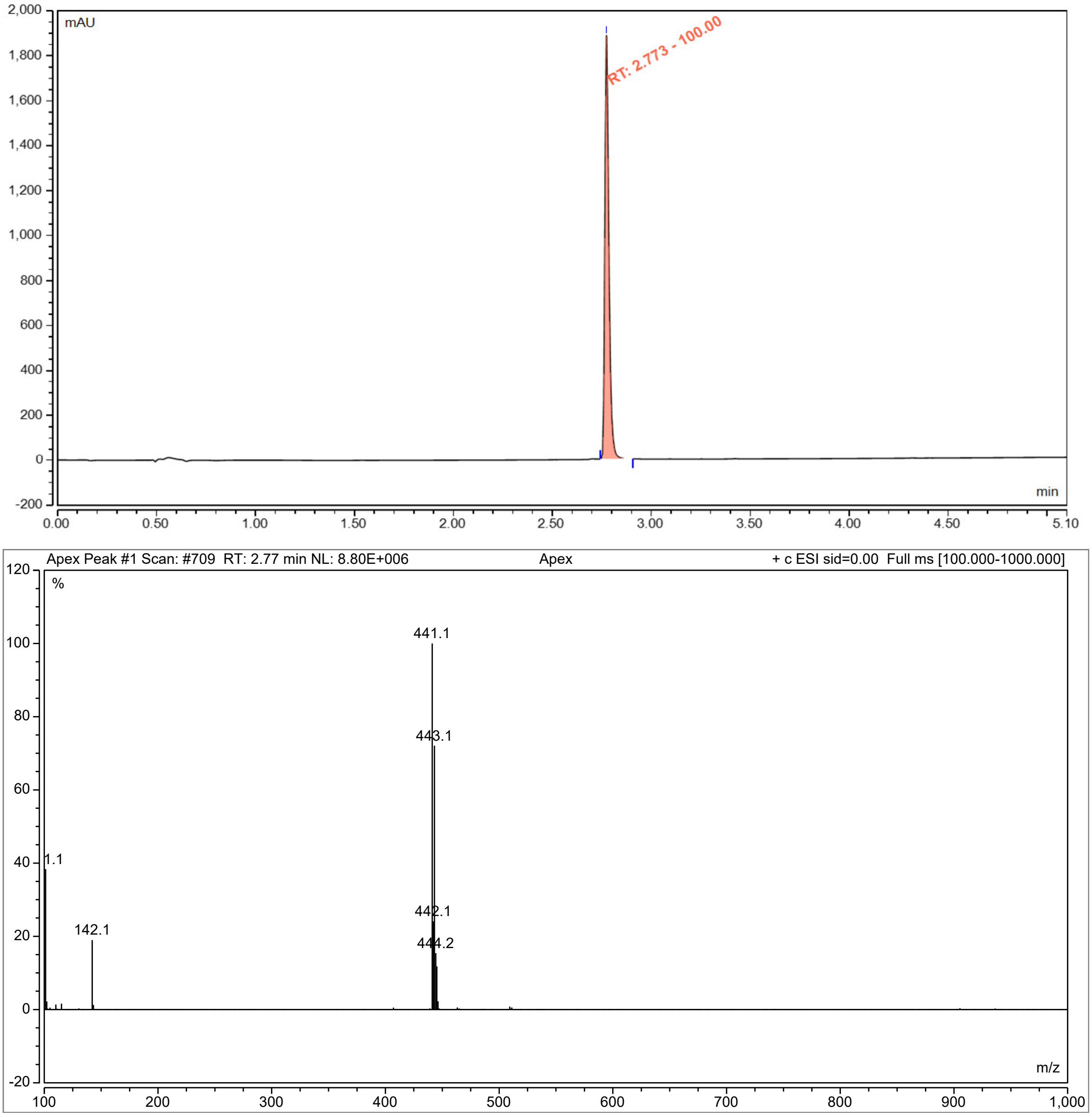

*Figure S38. HPLC-MS UV chromatogram and positive ESI-MS of **9d**.*

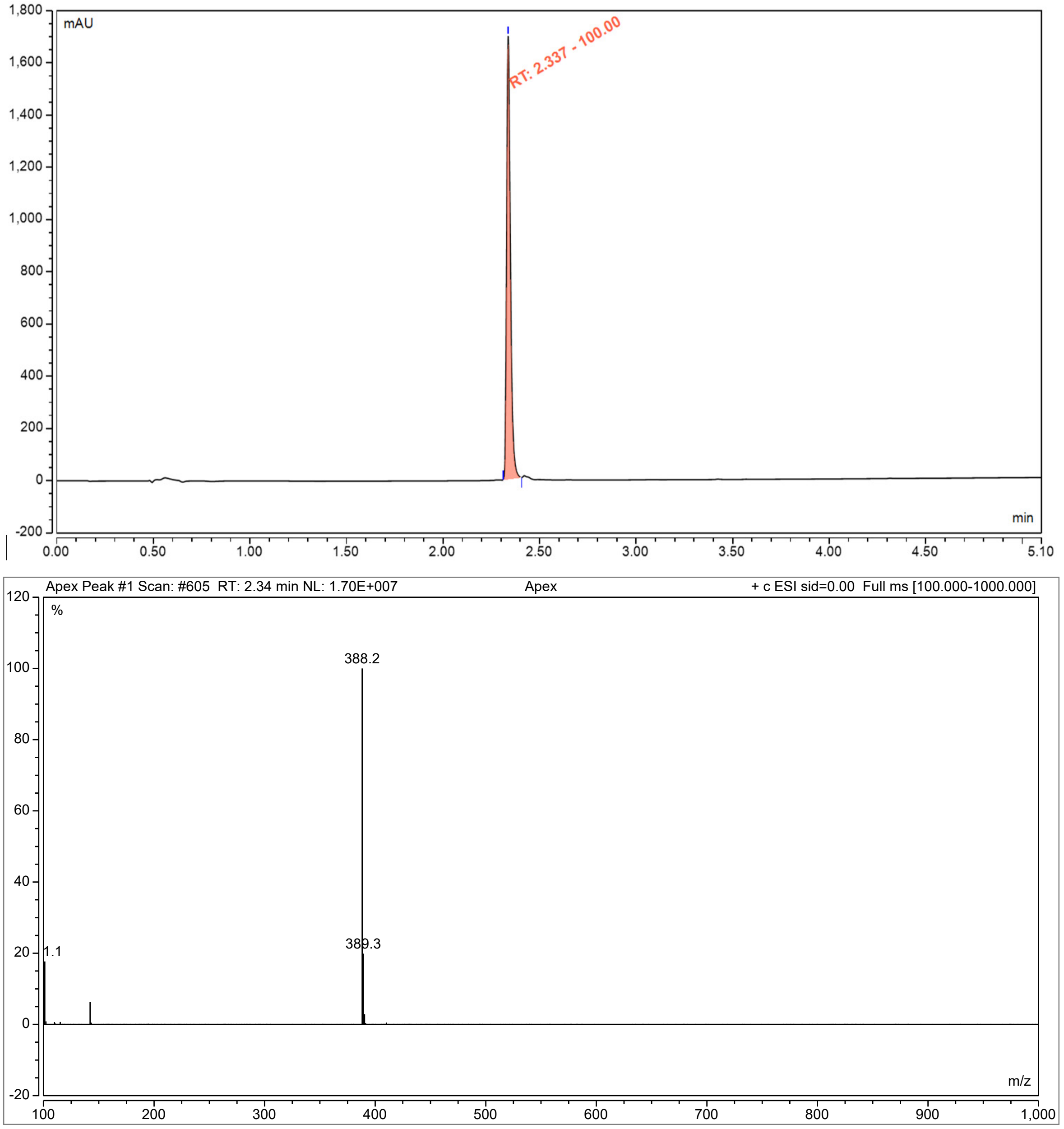

*Figure S39. HPLC-MS UV chromatogram and positive ESI-MS of **9e**.*

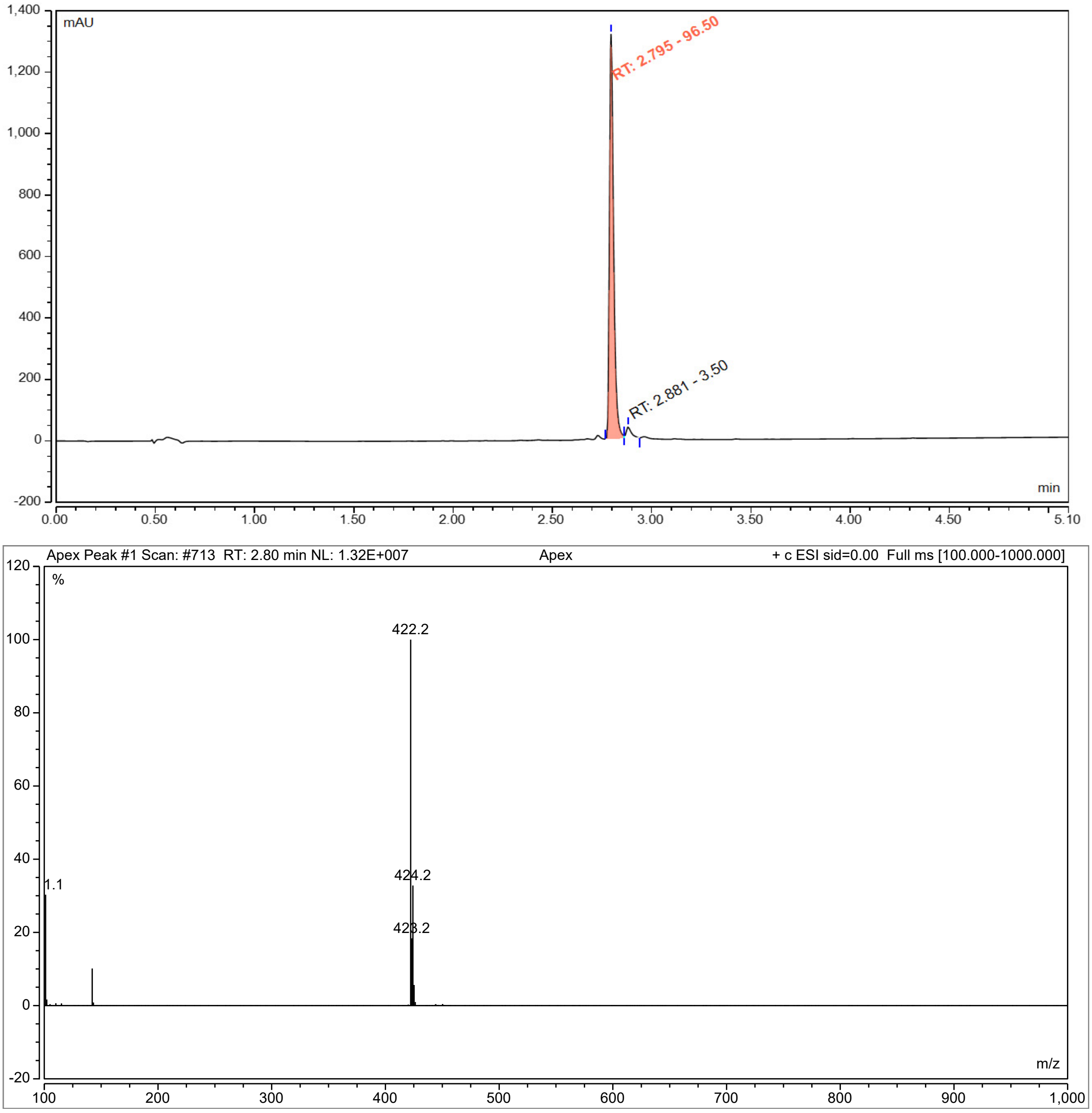

*Figure S40. HPLC-MS UV chromatogram and positive ESI-MS of **9f**.*

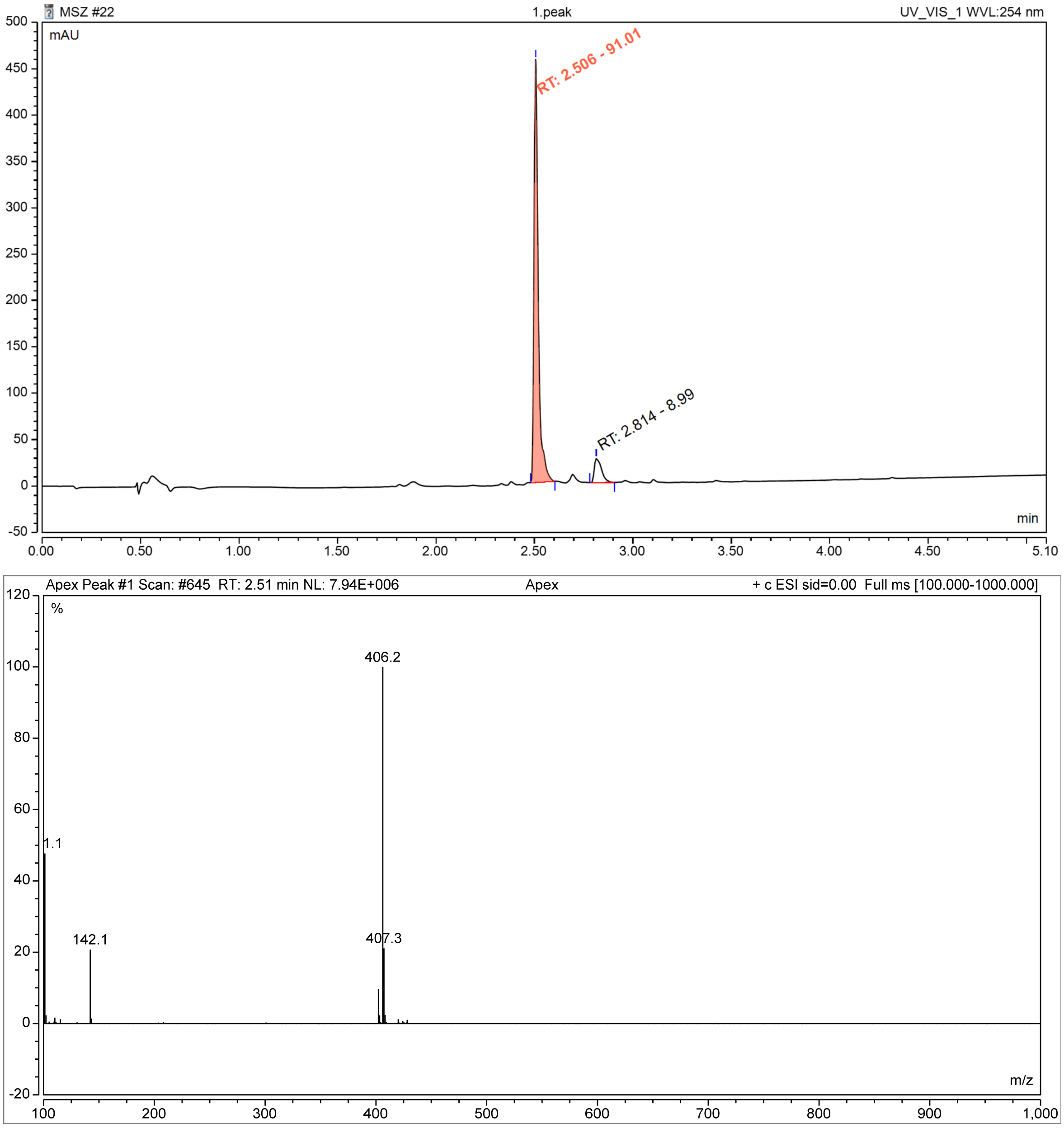

*Figure S41. HPLC-MS UV chromatogram and positive ESI-MS of **9g**.*

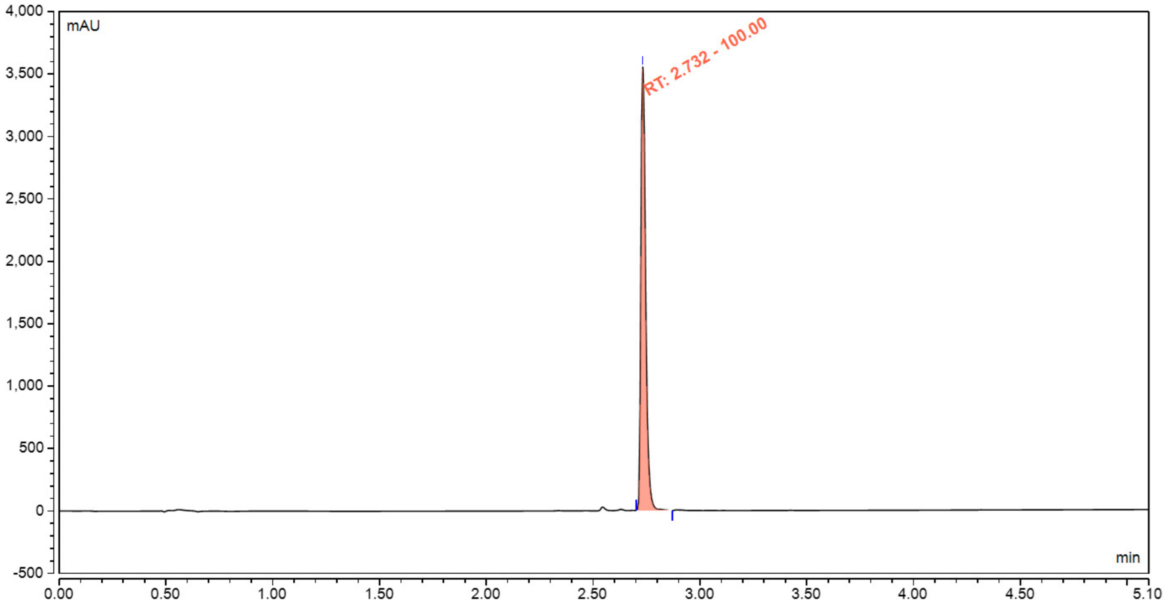

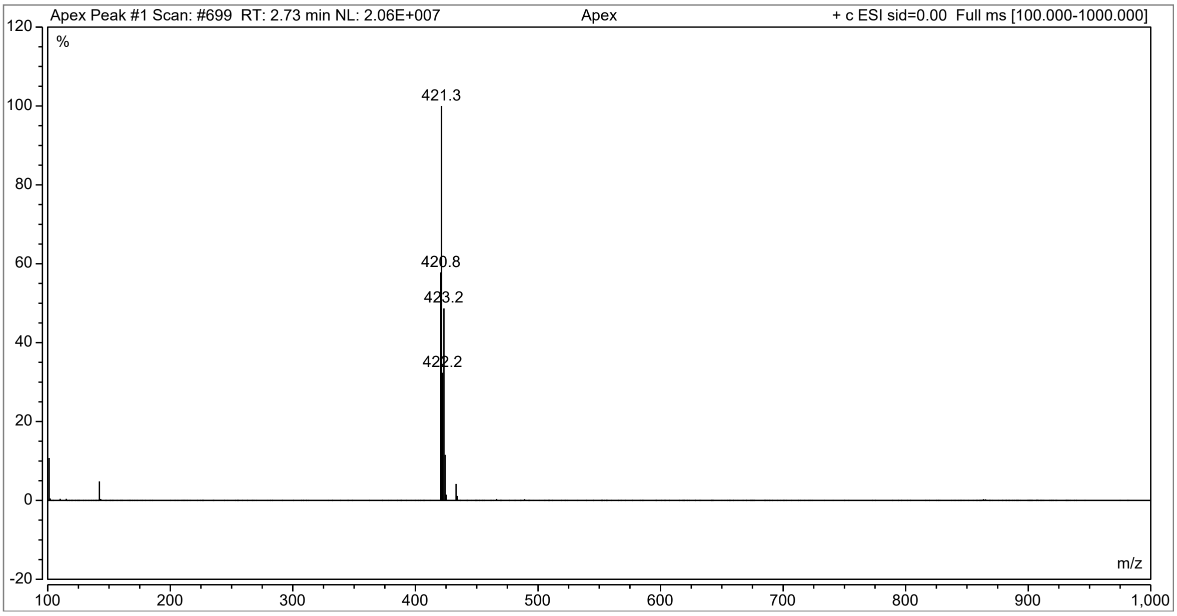

*Figure S42. HPLC-MS UV chromatogram and positive ESI-MS of **9h**.*

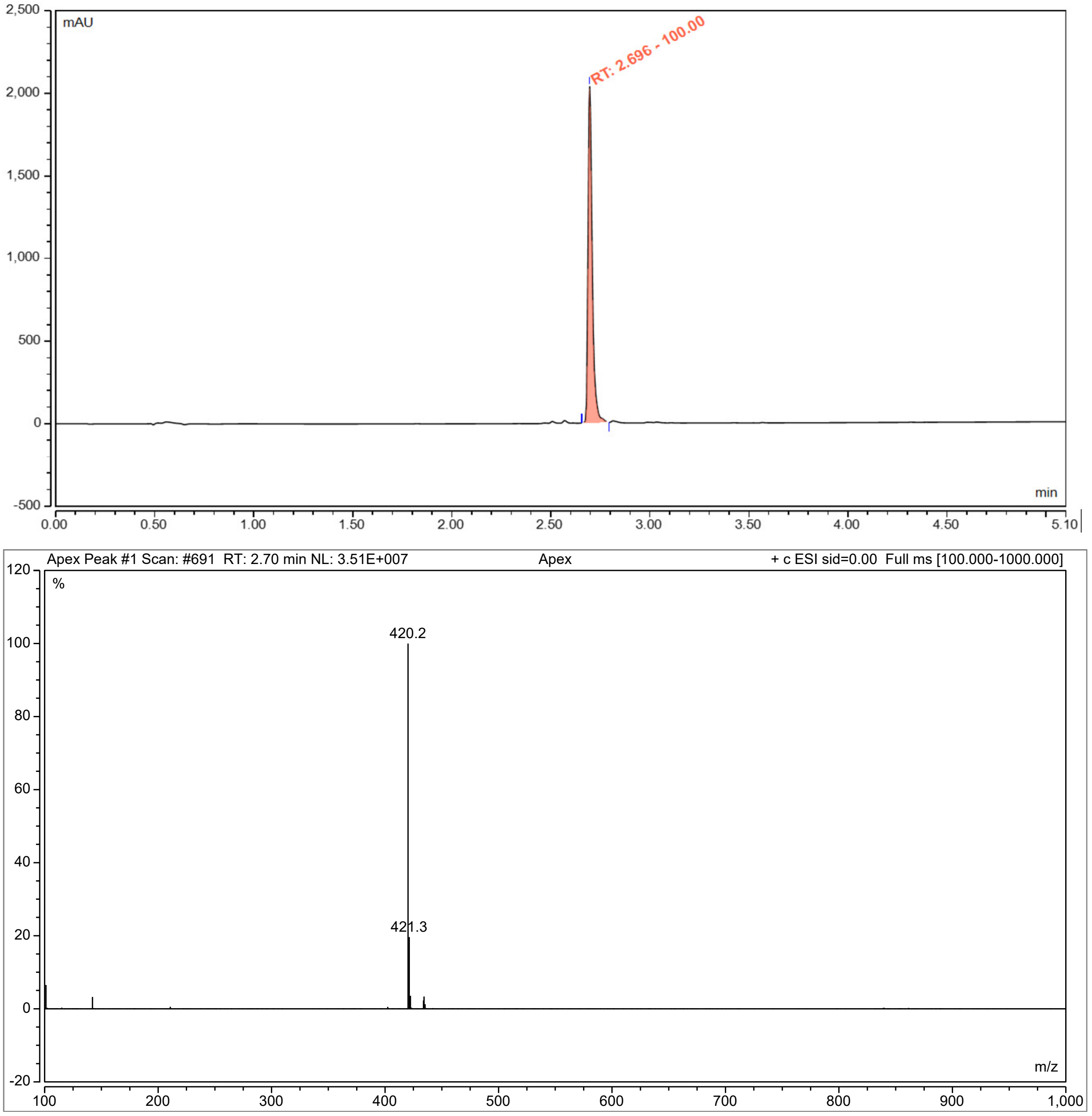

*Figure S43. HPLC-MS UV chromatogram and positive-ESI MS of **9i**.*

1.6 Synthesis of aryl substituents with an Fmoc-protected amino group

***N*-Fmoc-2-aminobenzoic acid (SI-1).** To 2-aminobenzoic acid (274 mg, 2.0 mmol), Fmoc-Cl (517 mg, 2.0 mmol) was added, and the mixture was dissolved in dioxane (5 mL). Then, saturated aqueous sodium bicarbonate solution (0.1 mL) was added to the solution, and stirred overnight. The solution was extracted with saturated aqueous ammonium chloride solution and ethyl acetate (3 x 50 mL). The organic layers

were combined dried over sulfate and filtered. Then, the solvent was removed under reduced pressure. The compound was obtained as white solid and it was used without further purification (650 mg, 90%). ^{1}H NMR (500 MHz, DMSO-d_6): δ 11.21 (s, 1H), 8.14 (d, *J* = 8.6 Hz, 1H), 7.97 (dd, *J* = 7.9, 1.7 Hz, 1H), 7.92 (d, *J* = 7.5 Hz, 2H), 7.69 (d, *J* = 7.5 Hz, 2H), 7.53 (t, *J* = 7.8 Hz, 1H), 7.46–7.39 (m, 2H), 7.35 (td, *J* = 7.5, 1.2 Hz, 2H), 7.08 (t, *J* = 7.6 Hz, 1H), 4.48 (d, *J* = 6.8 Hz, 2H), 4.35 (t, *J* = 6.8 Hz, 1H); HRMS (ESI) (*m/z*): calcd for $C_{22}H_{16}NO_4$ 358.1073, found 358.1083 $[M - H]^-$.

***N*-Fmoc-2-amino-3-chlorobenzoic acid (SI-2).** To 2-amino-3-chlorobenzoic acid (348 mg, 2.0 mmol) Fmoc-Cl (517 mg, 2.0 mmol) was added and the mixture was dissolved in dioxane (5 mL). Then, saturated aqueous sodium bicarbonate solution (0.1 mL) was added to the solution, and stirred overnight. The solution was extracted with saturated aqueous ammonium chloride solution and ethyl acetate (3 x 50 mL). The organic layers were combined and dried over sodium sulfate and filtered. Then, the solvent was removed under reduced pressure. The compound was obtained as white solid and it was used without further purification (660 mg, 84%). ^{1}H NMR (500 MHz, DMSO-d_6): δ 13.04 (s, 1H), 7.96–7.80 (m, 2H), 7.83–7.70 (m, 2H), 7.71–7.53 (m, 1H), 7.51–7.14 (m, 6H), 4.36–4.29 (m, 2H), 4.29–4.23 (m, 1H); HRMS (ESI) (*m/z*): calcd for $C_{22}H_{17}ClNO_4$ 394.0846, found 394.0839 $[M + H]^+$.

***N*-Fmoc-2-amino-6-fluorobenzoic acid (SI-3).** To 2-amino-6-fluorobenzoic acid (310 mg, 2.0 mmol) Fmoc-Cl (517 mg, 2.0 mmol) was added and the mixture was dissolved in dioxane (5 mL). Then, saturated aqueous sodium bicarbonate solution (0.1 mL) was added to the solution, and stirred overnight. The solution was extracted with saturated aqueous ammonium chloride solution and ethyl acetate (3 x 50 mL). The organic layers were combined and dried over sodium sulfate and filtered. Then, the solvent was removed under reduced pressure. The compound was obtained as white solid and it was used without further purification (671 mg, 89%). ^{1}H NMR (500 MHz, acetone-d_6): δ 10.35 (s, 1H), 8.12 (d, *J* = 8.5 Hz, 1H), 7.88 (dd, *J* = 7.5, 3.6 Hz, 2H), 7.72 (d, *J* = 7.3 Hz, 2H), 7.59–7.54 (m, 1H), 7.43 (td, *J* = 7.5, 2.0 Hz, 2H), 7.35 (t, *J* = 7.3 Hz, 2H), 6.95–6.90 (m, 1H), 4.51 (dd, *J* = 7.0, 2.5 Hz, 3H), 4.37–4.34 (m, 1H); HRMS (ESI) (*m/z*): calcd for $C_{22}H_{15}FNO_4$ 376.0990, found 376.0985 $[M - H]^-$.

1.7 NMR-based structure elucidation

1.7.1 ^{1}H and ^{13}C-spectra of compounds 1 and 3

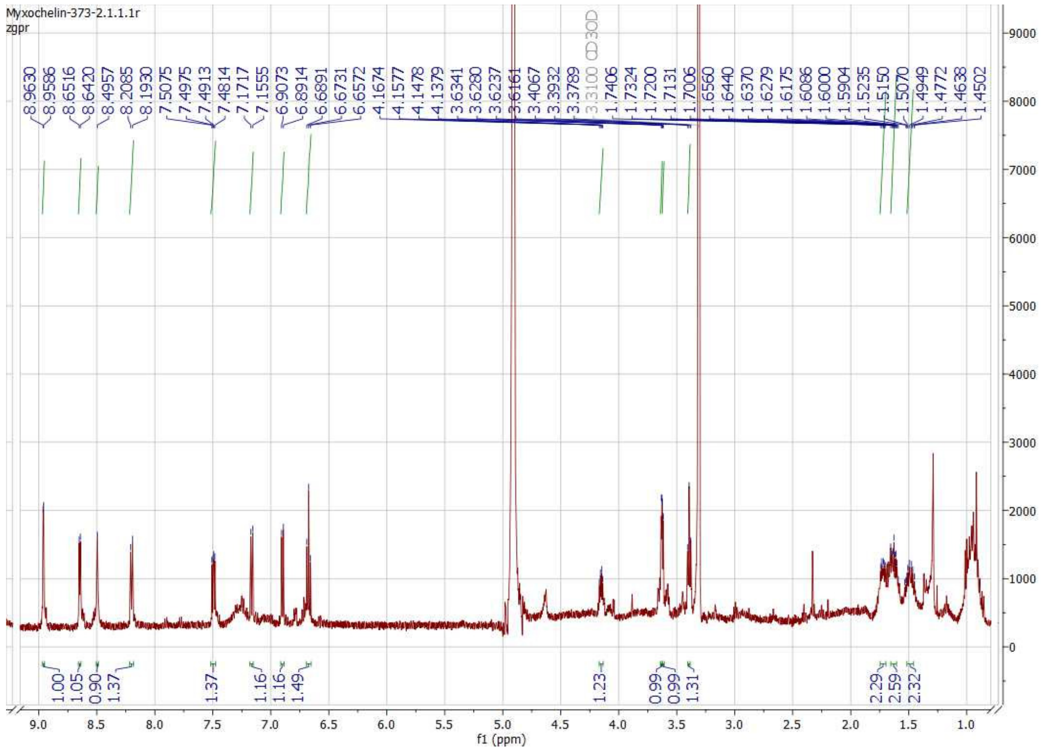

*Figure S44. ^{1}H NMR spectrum of **1** in methanol-d_4.*

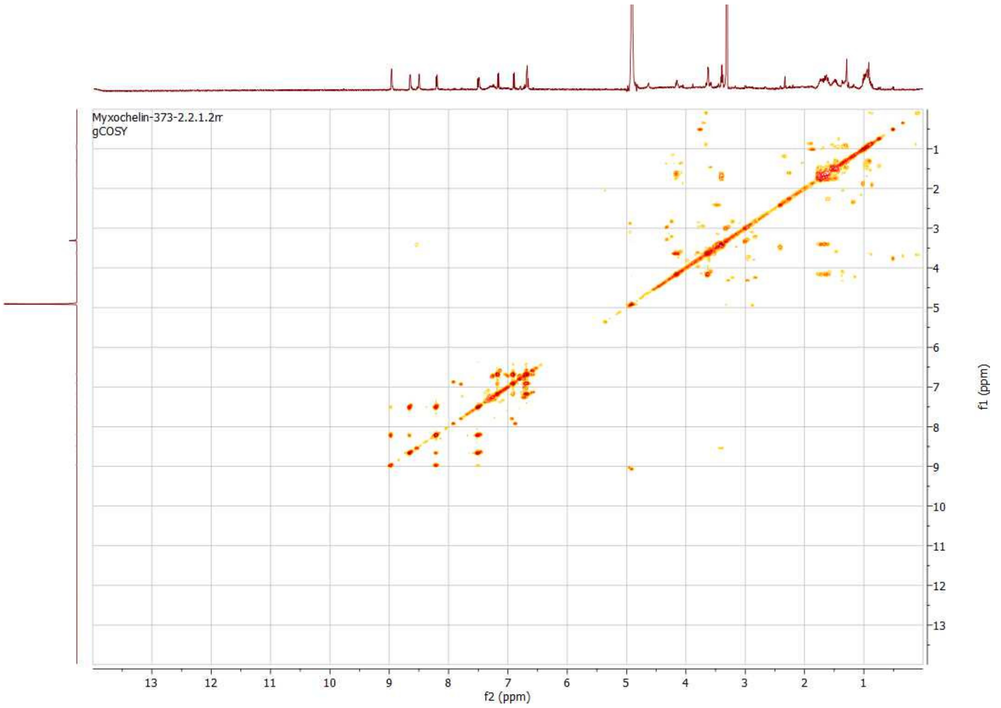

*Figure S45. ^{1}H-^{1}H COSY spectrum of **1** in methanol-d_4.*

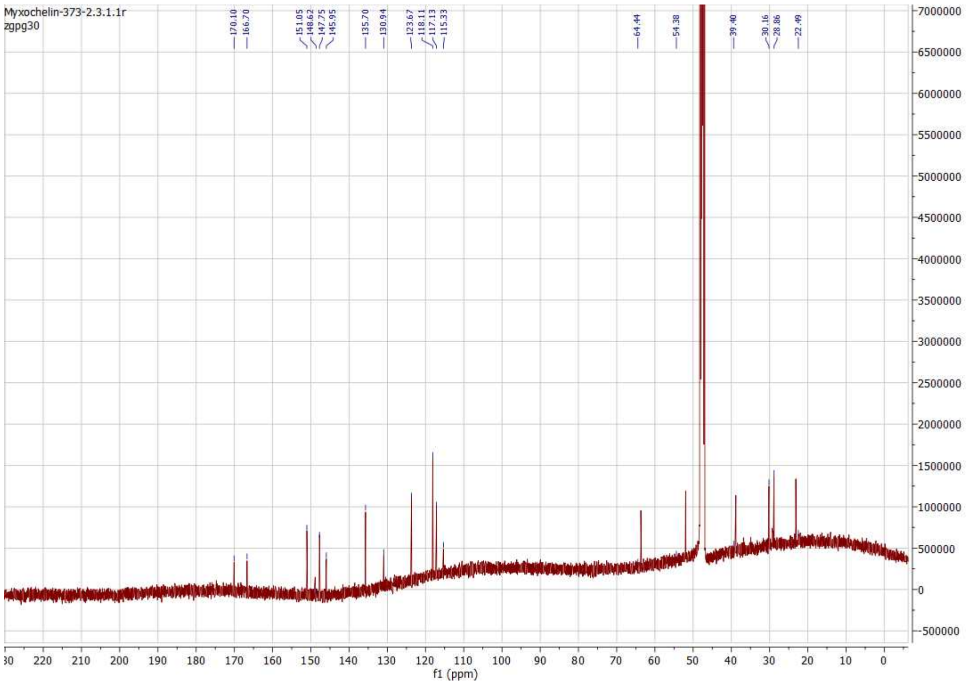

Figure S46. ^{13}C NMR spectrum of 1 in methanol-d_4.

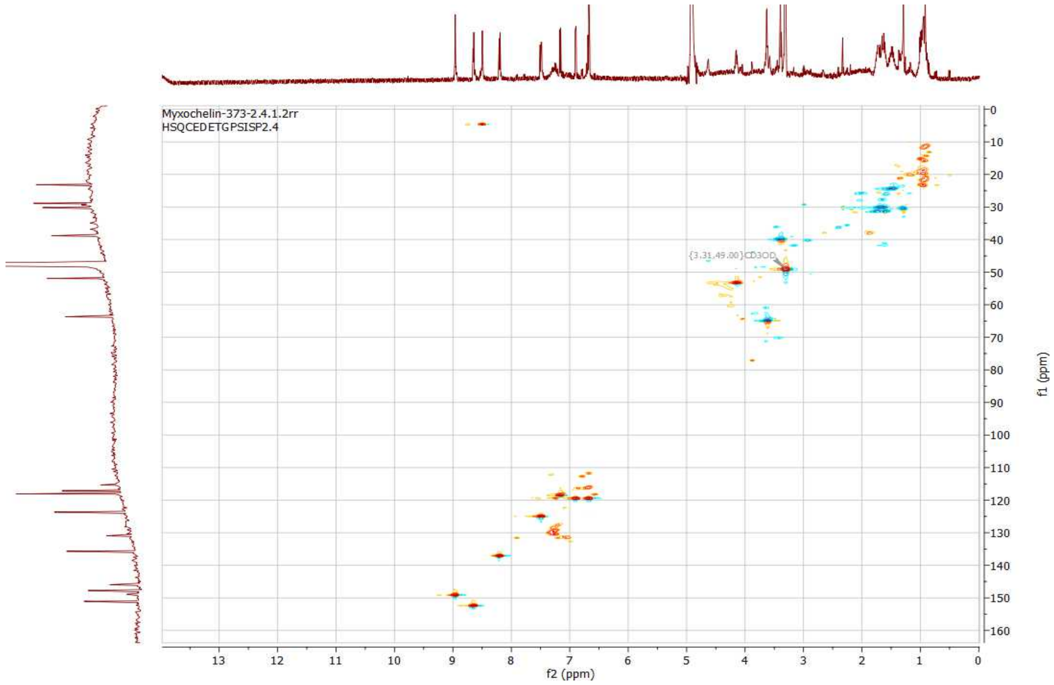

*Figure S47. HSQC spectrum of **1** in methanol-d_4.*

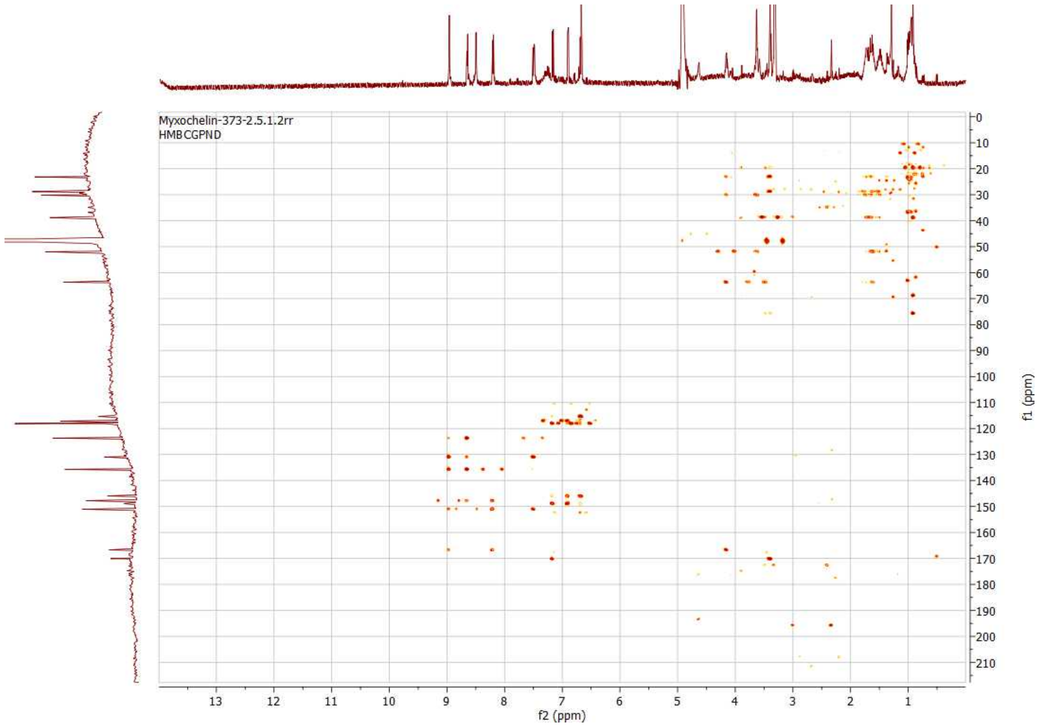

*Figure S48. HMBC spectrum of **1** in methanol-d_4.*

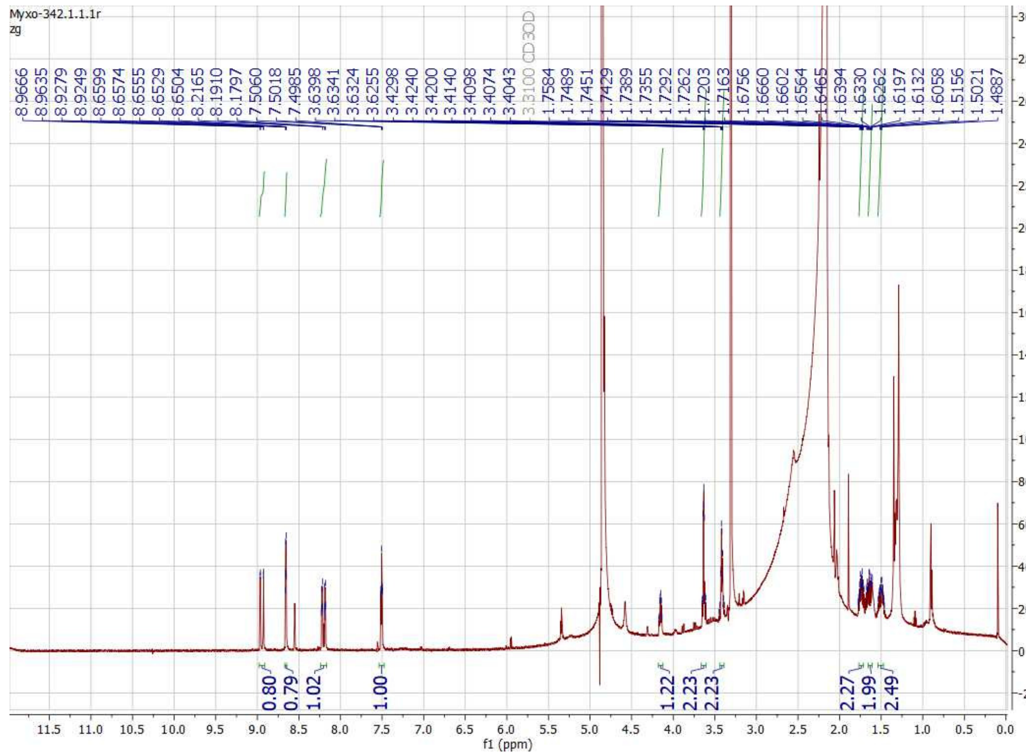

*Figure S49. ^{1}H NMR spectrum of **3** in methanol-d_4.*

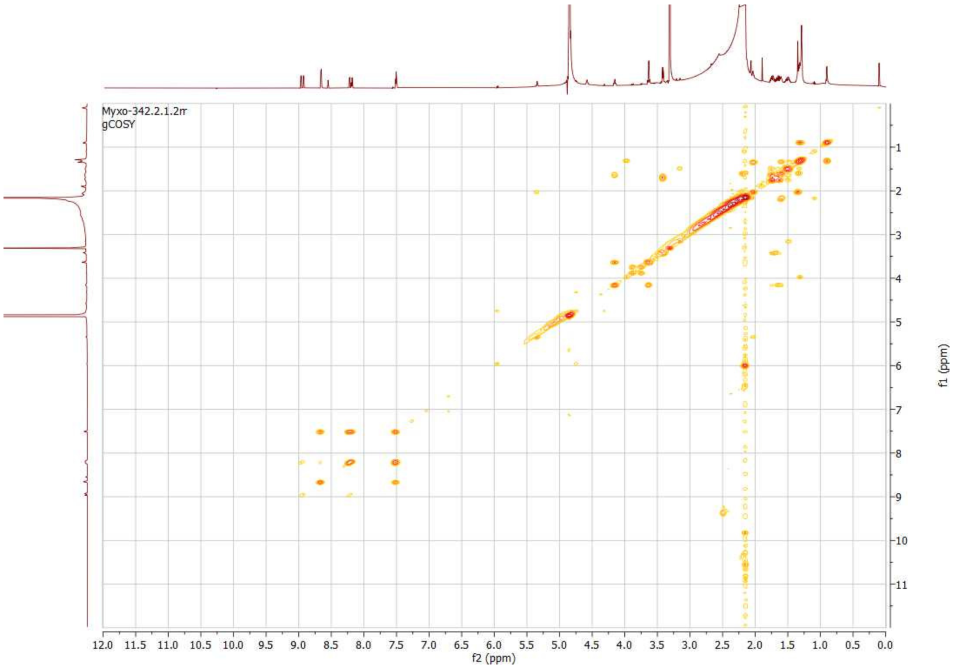

*Figure S50. ^{1}H-^{1}H COSY spectrum of **3** in methanol-d_4.*

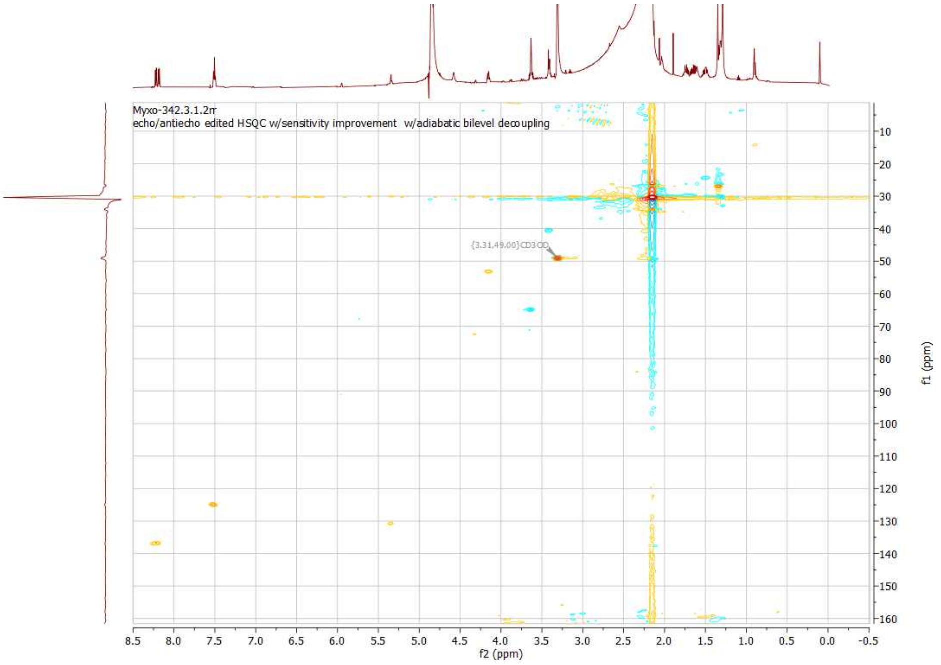

*Figure S51. HSQC spectrum of **3** in methanol-d_4.*

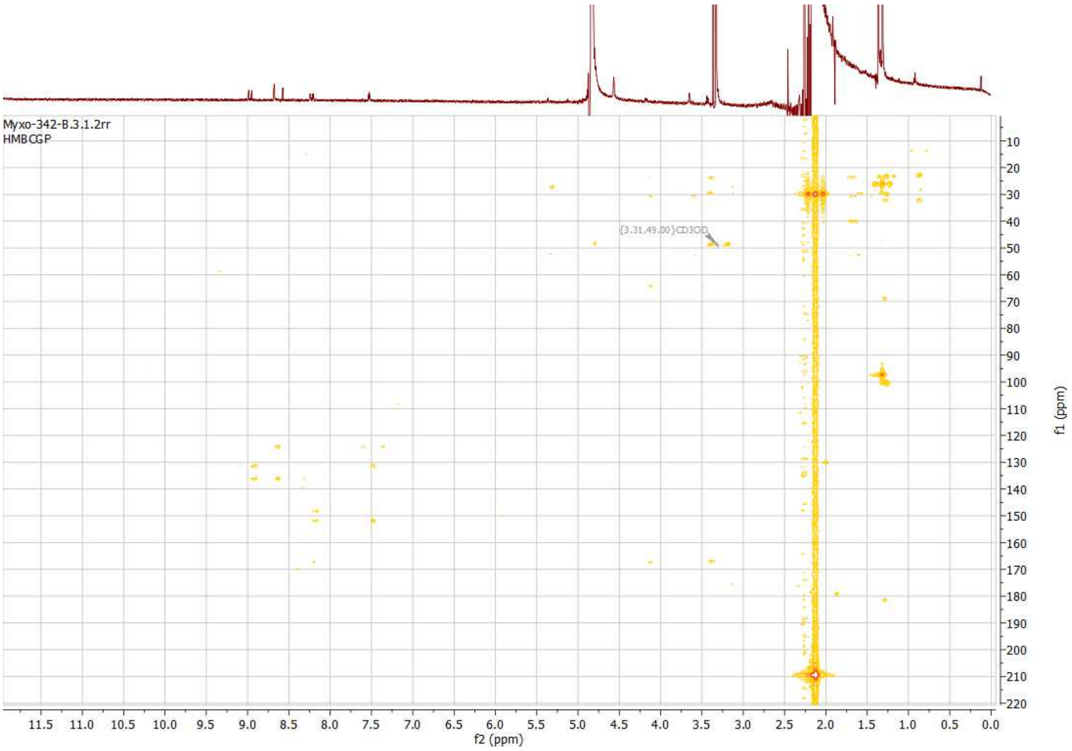

*Figure S52. HMBC spectrum of **3** in methanol-d_4.*

1.7.2 ^{1}H and ^{13}C spectra of the synthetic compounds

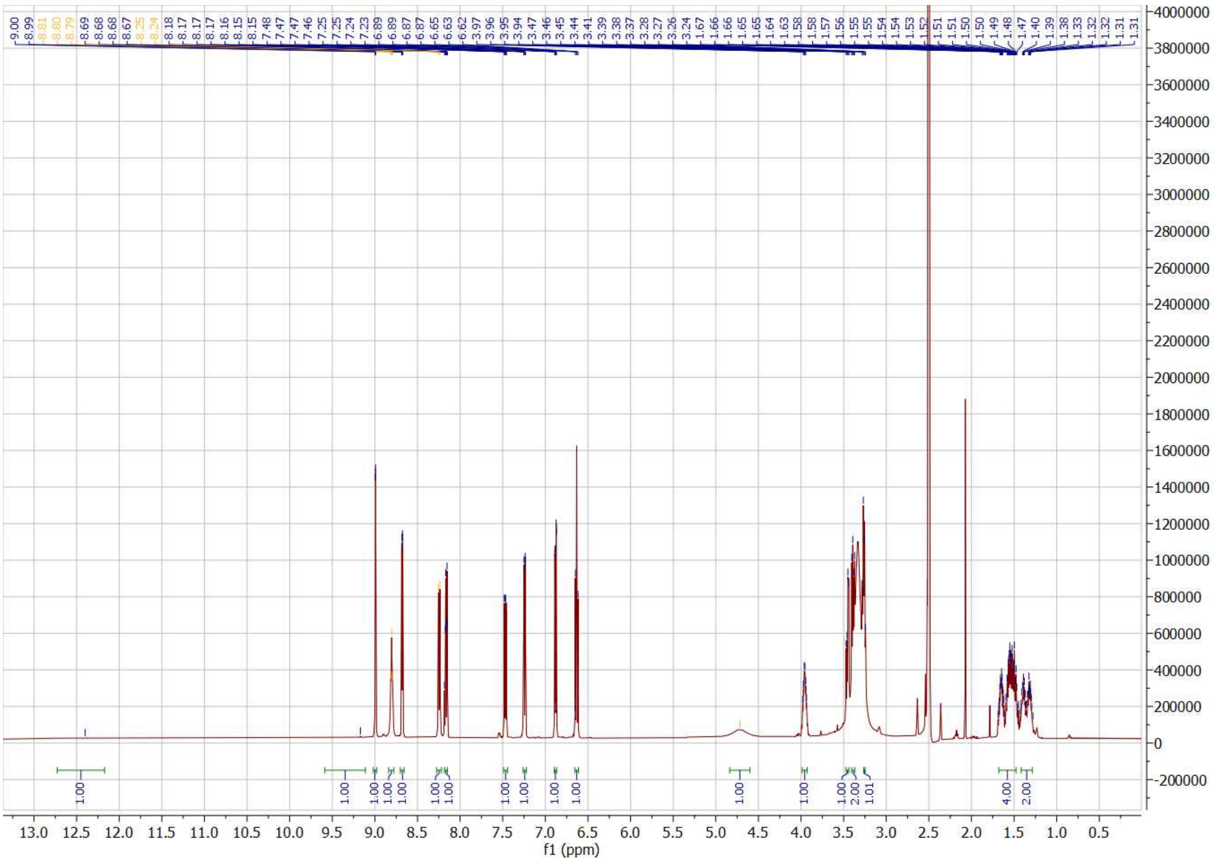

*Figure S53. ^{1}H NMR spectrum of **9a** in DMSO-d_6.*

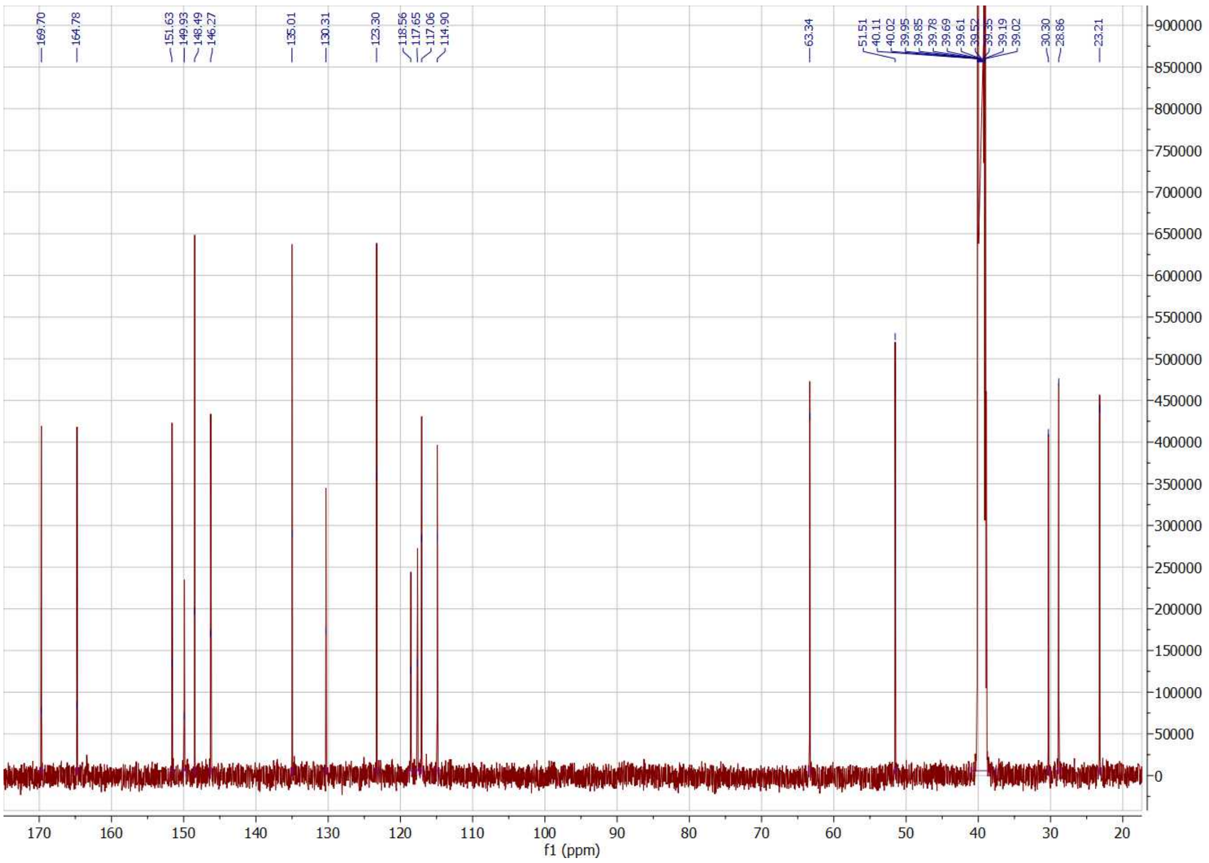

Figure S54. 13C NMR spectrum of 9a in DMSO-d6.

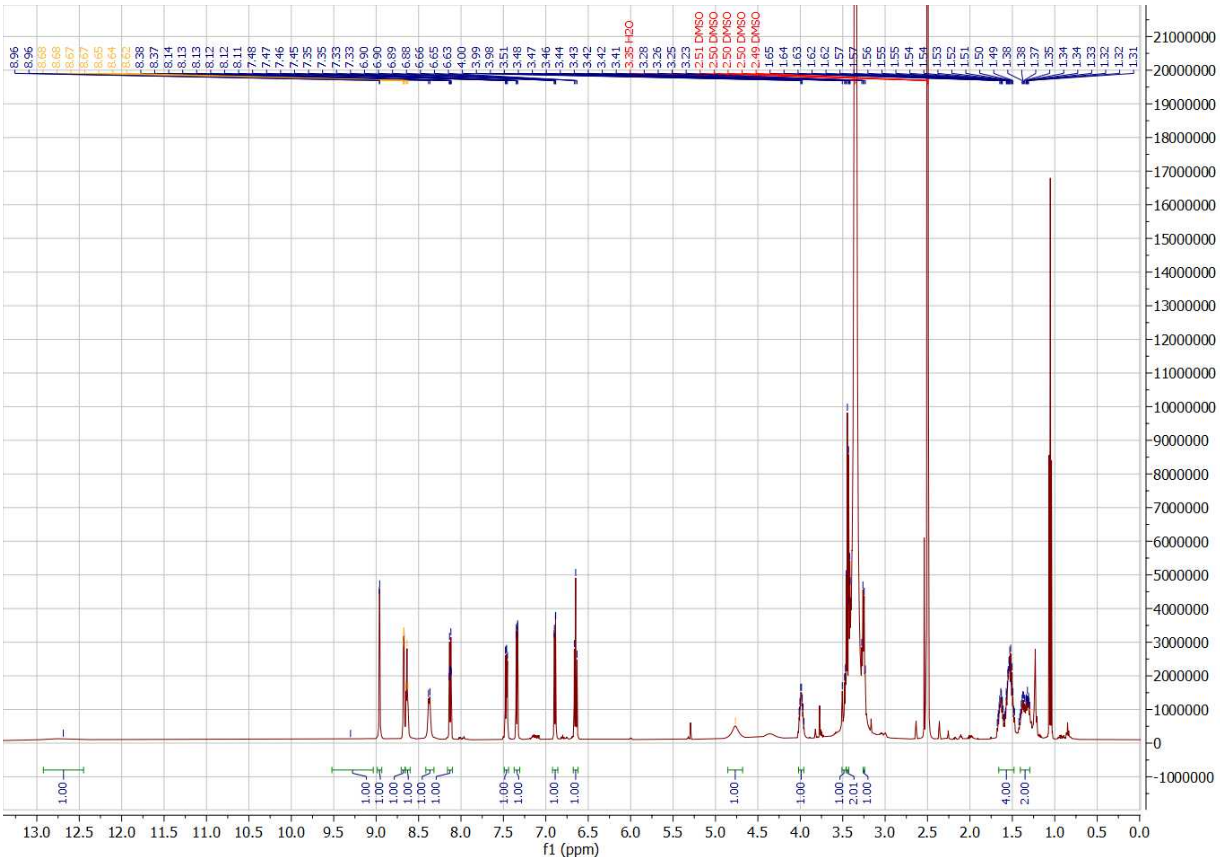

*Figure S55. ^1H NMR spectrum of **9b** in DMSO-d_6.*

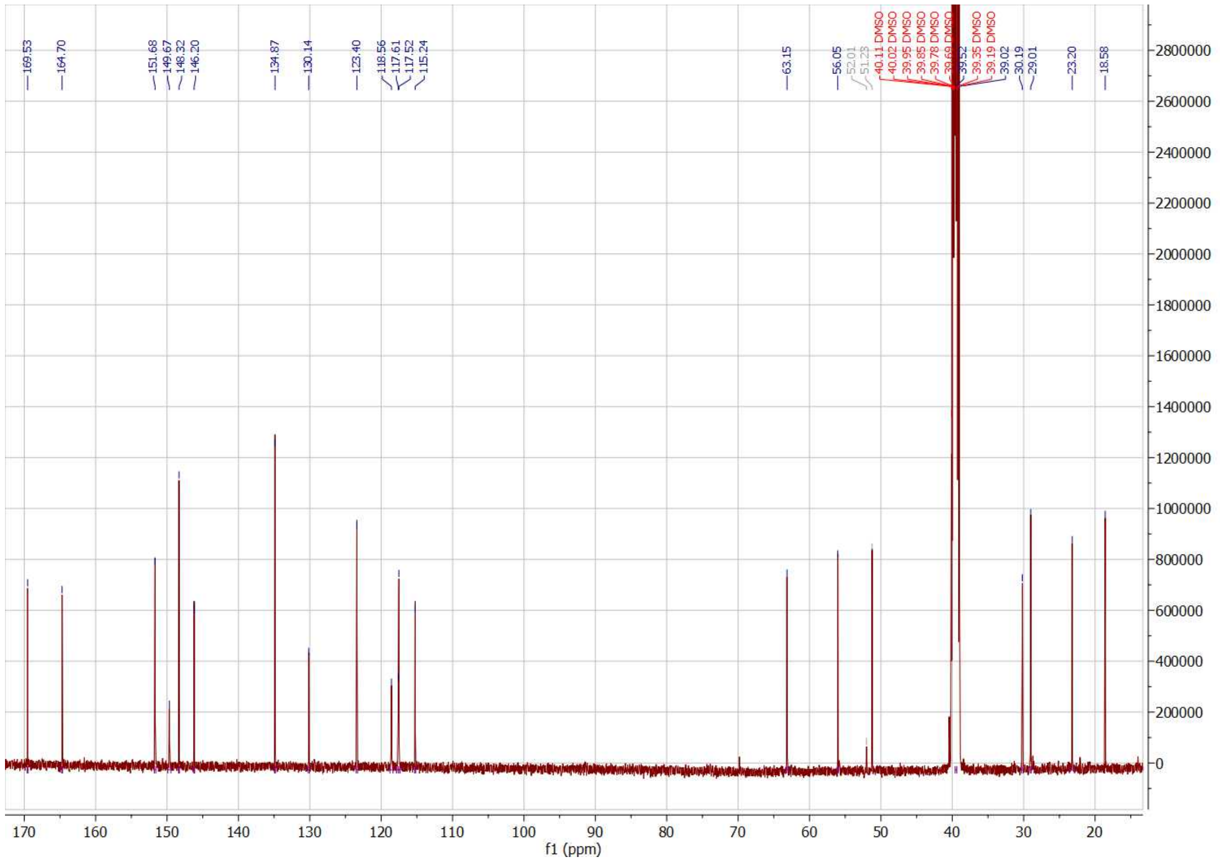

*Figure S56. ^{13}C NMR spectrum of **9b** in DMSO-d_6.*

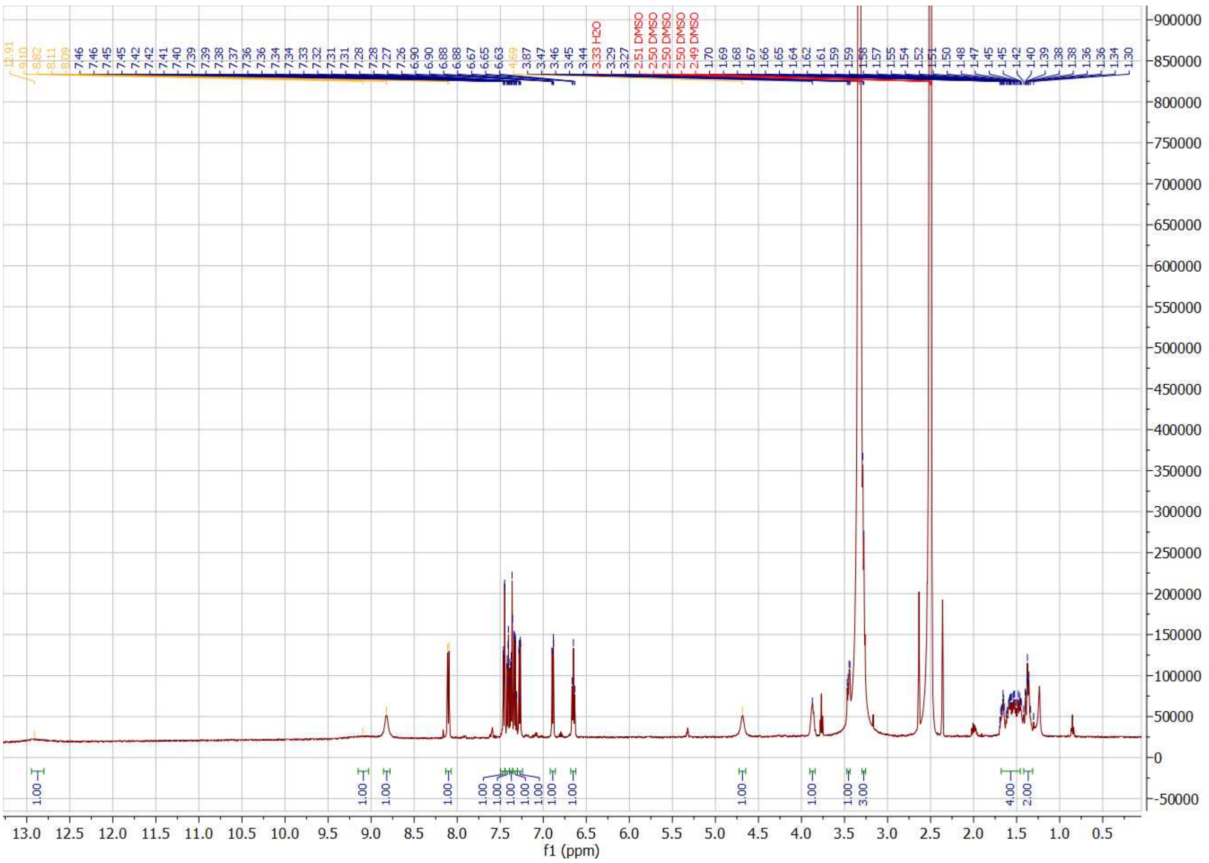

*Figure S57. ^{1}H NMR spectrum of **9c** in DMSO-d_6.*

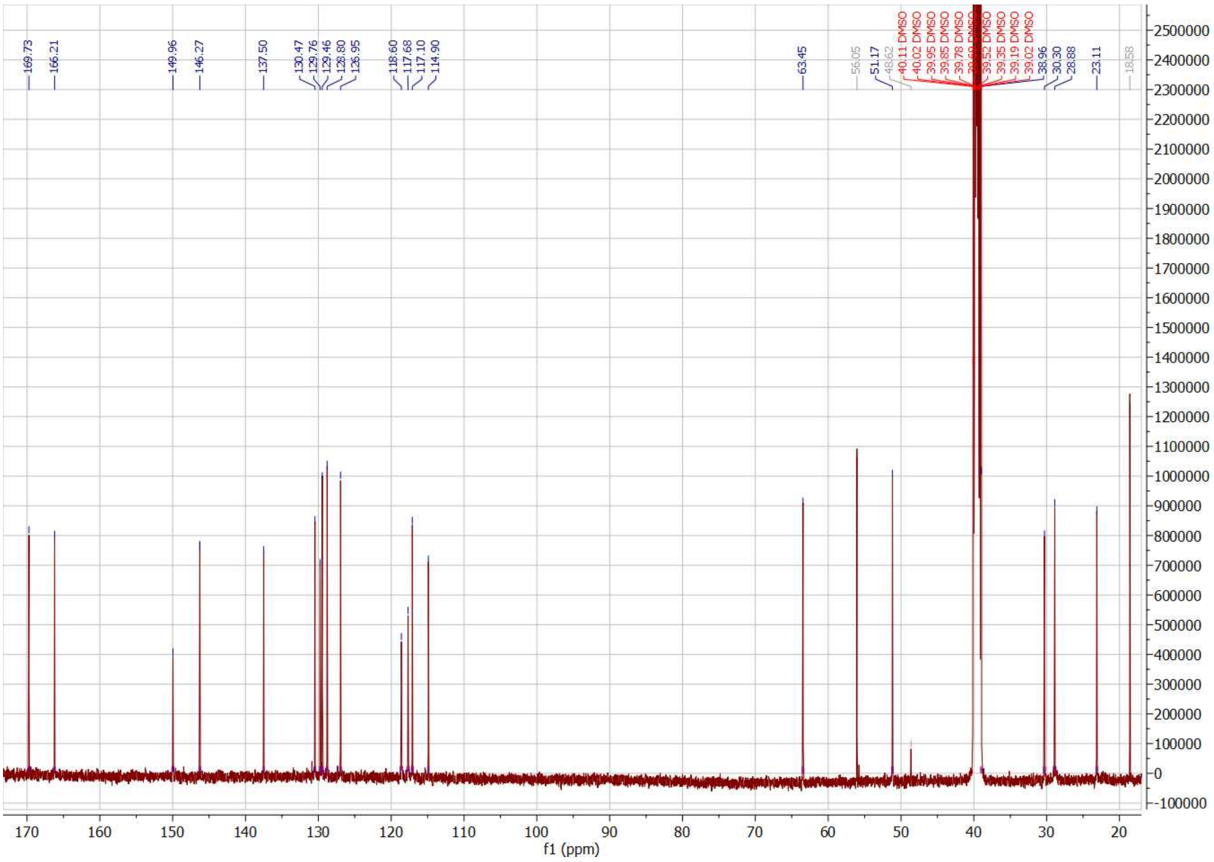

Figure S58. ^{13}C *NMR spectrum of* ***9c*** *in DMSO-*d_6*.*

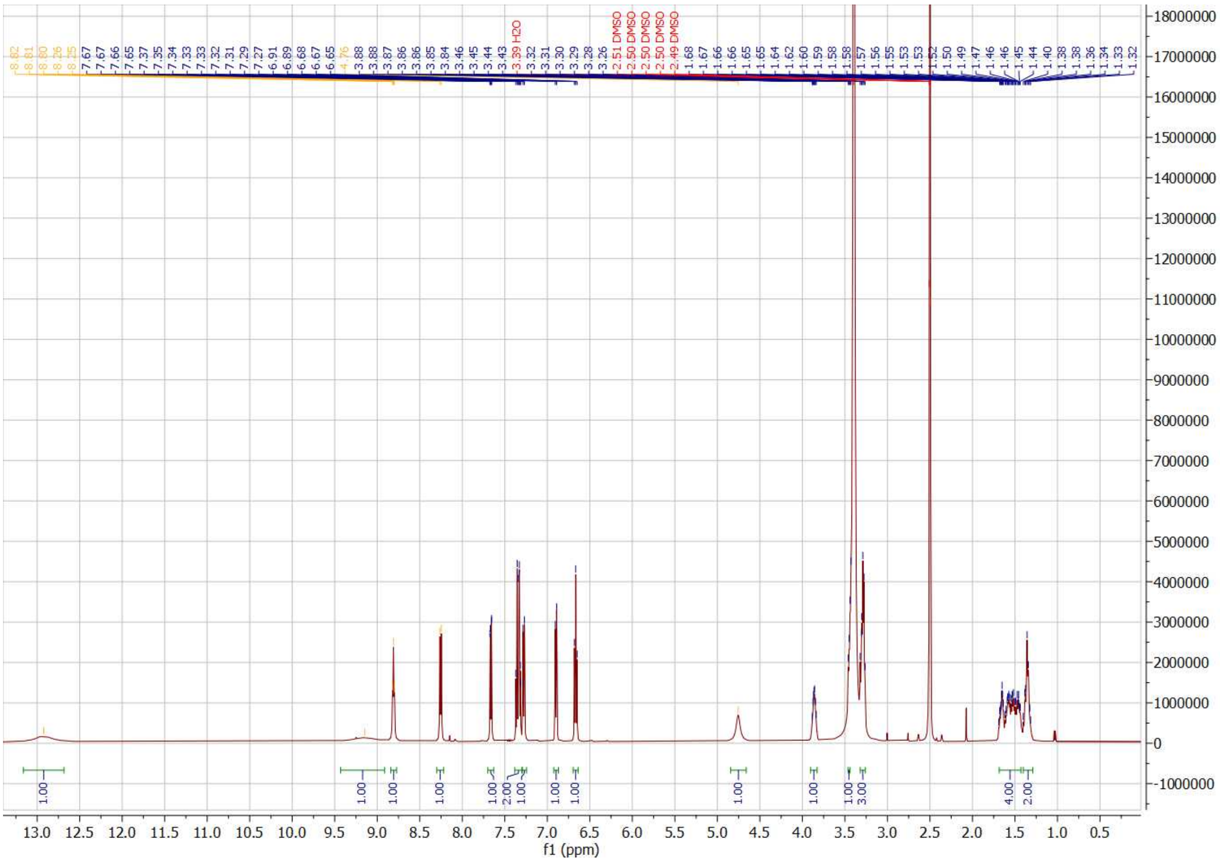

*Figure S59. ^{1}H NMR spectrum of **9d** in DMSO-d_6.*

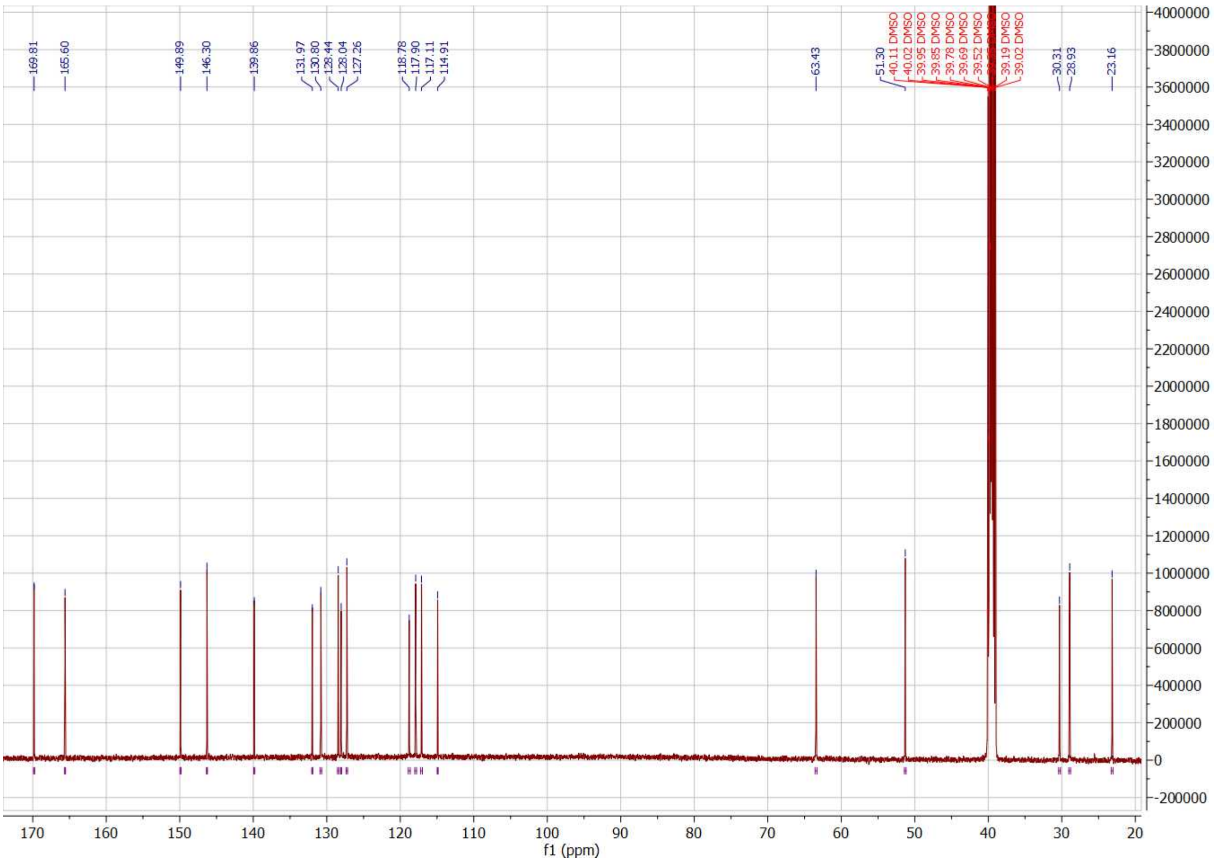

*Figure S60. ^{13}C NMR spectrum of **9d** in DMSO-d$_6$.*

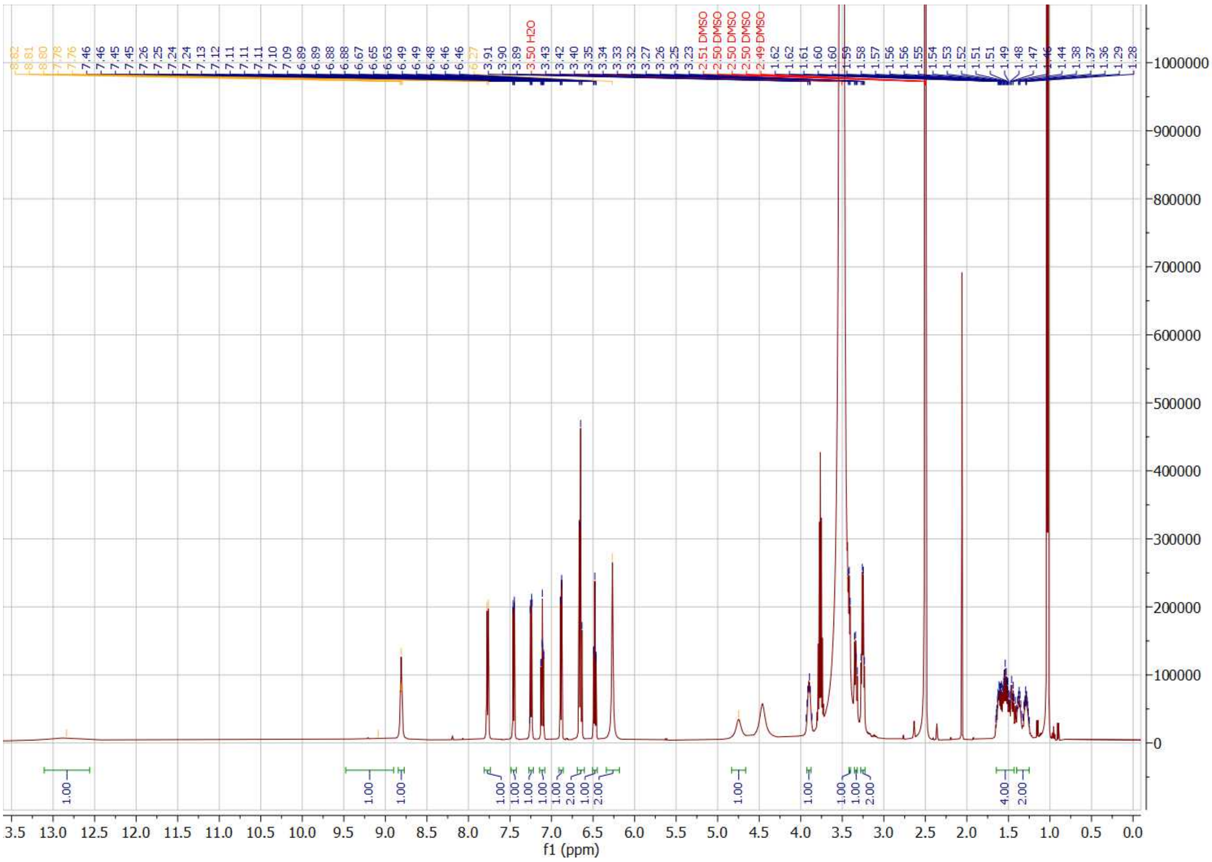

*Figure S61. ^{1}H NMR spectrum of **9e** in DMSO-d_6.*

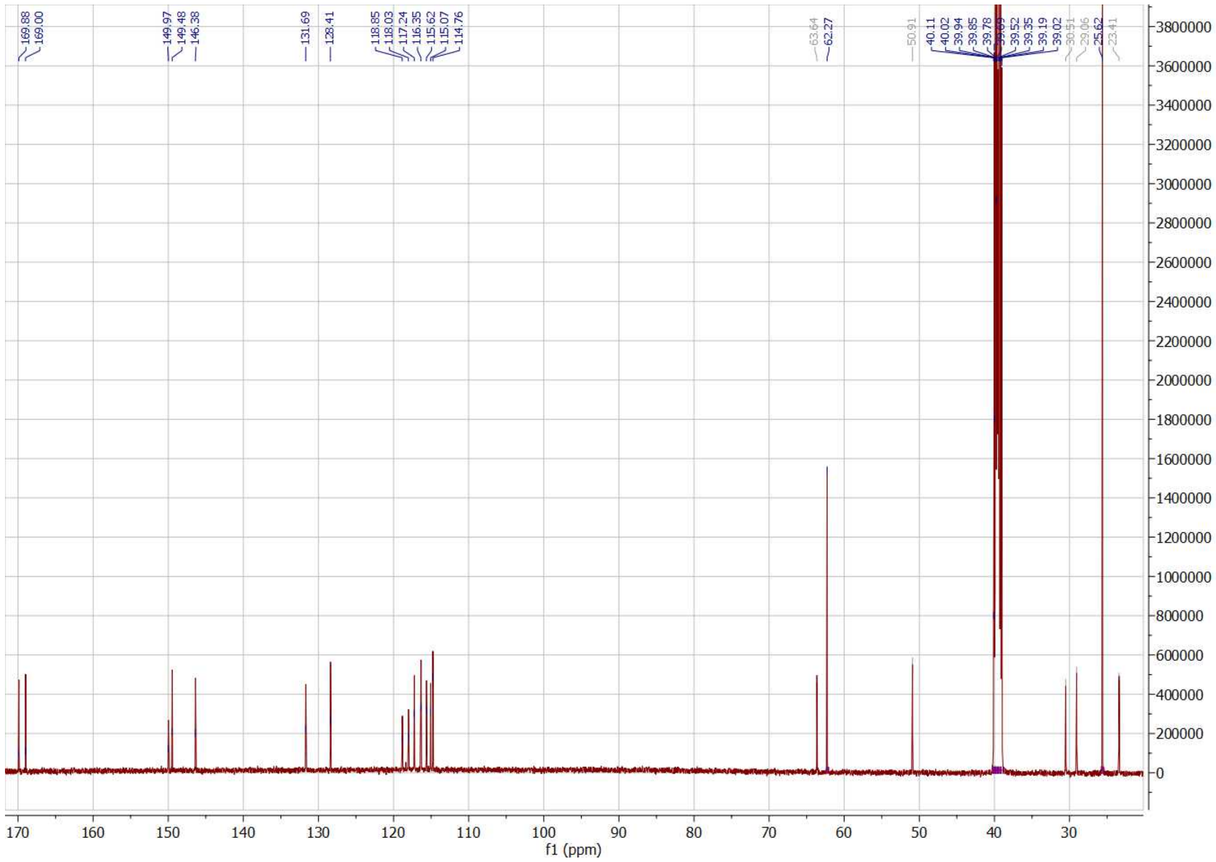

*Figure S62. ^{13}C NMR spectrum of **9e** in DMSO-d_6.*

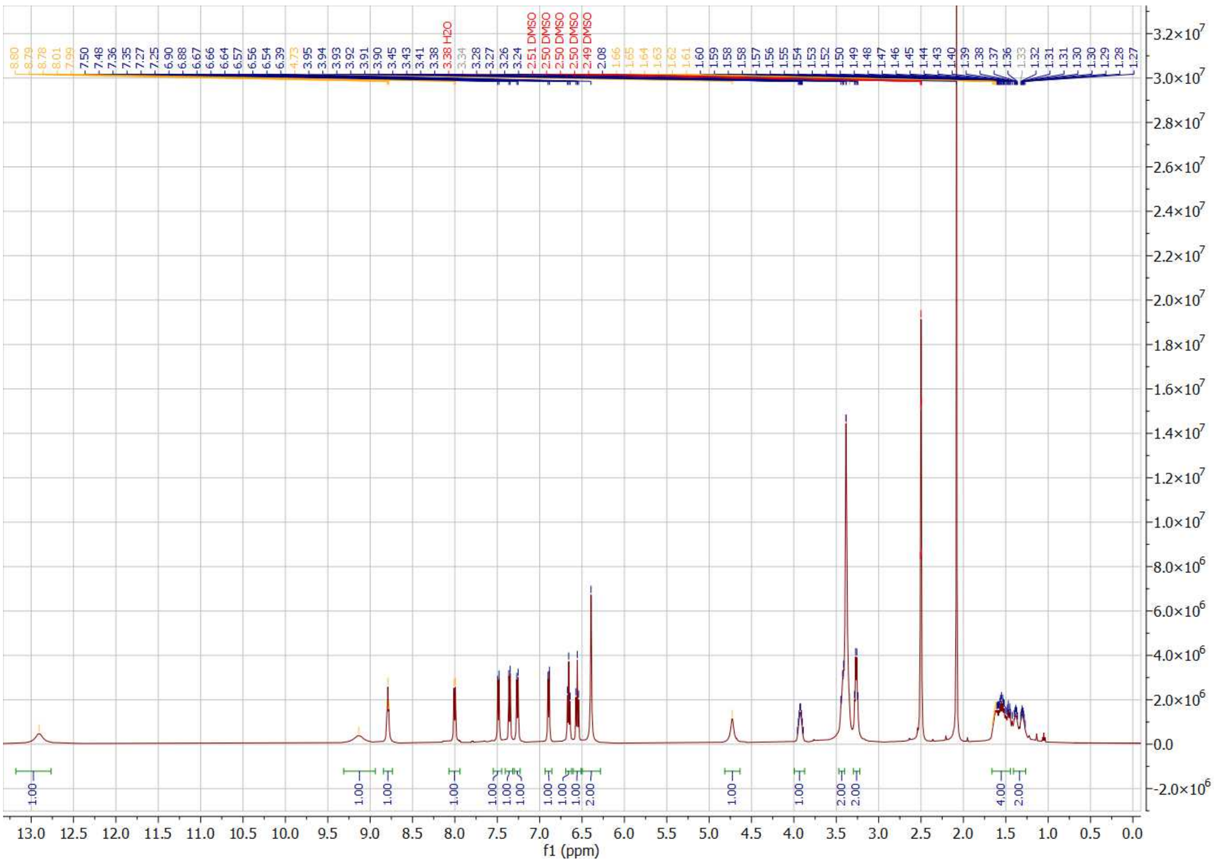

*Figure S63. ^{1}H NMR spectrum of **9f** in DMSO-d_6.*

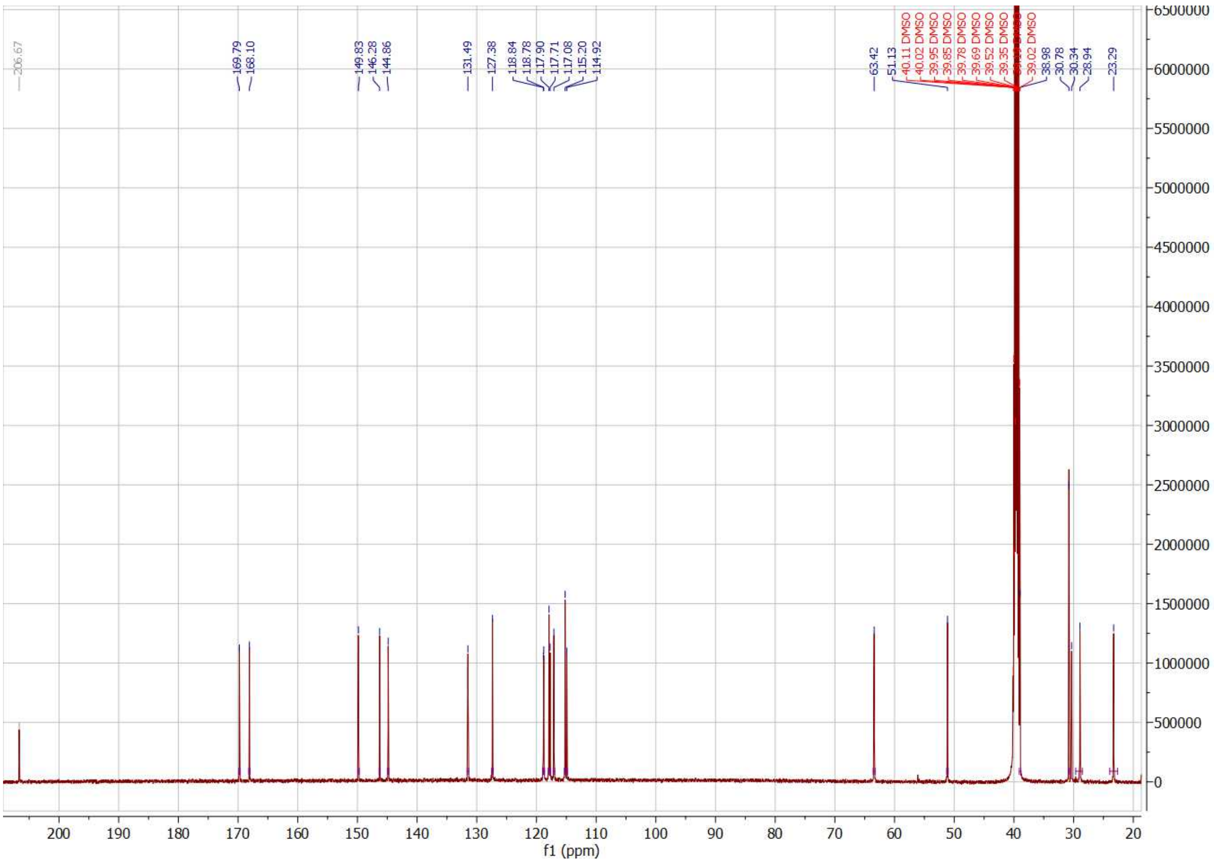

*Figure S64. ^{13}C NMR spectrum of **9f** in DMSO-d_6.*

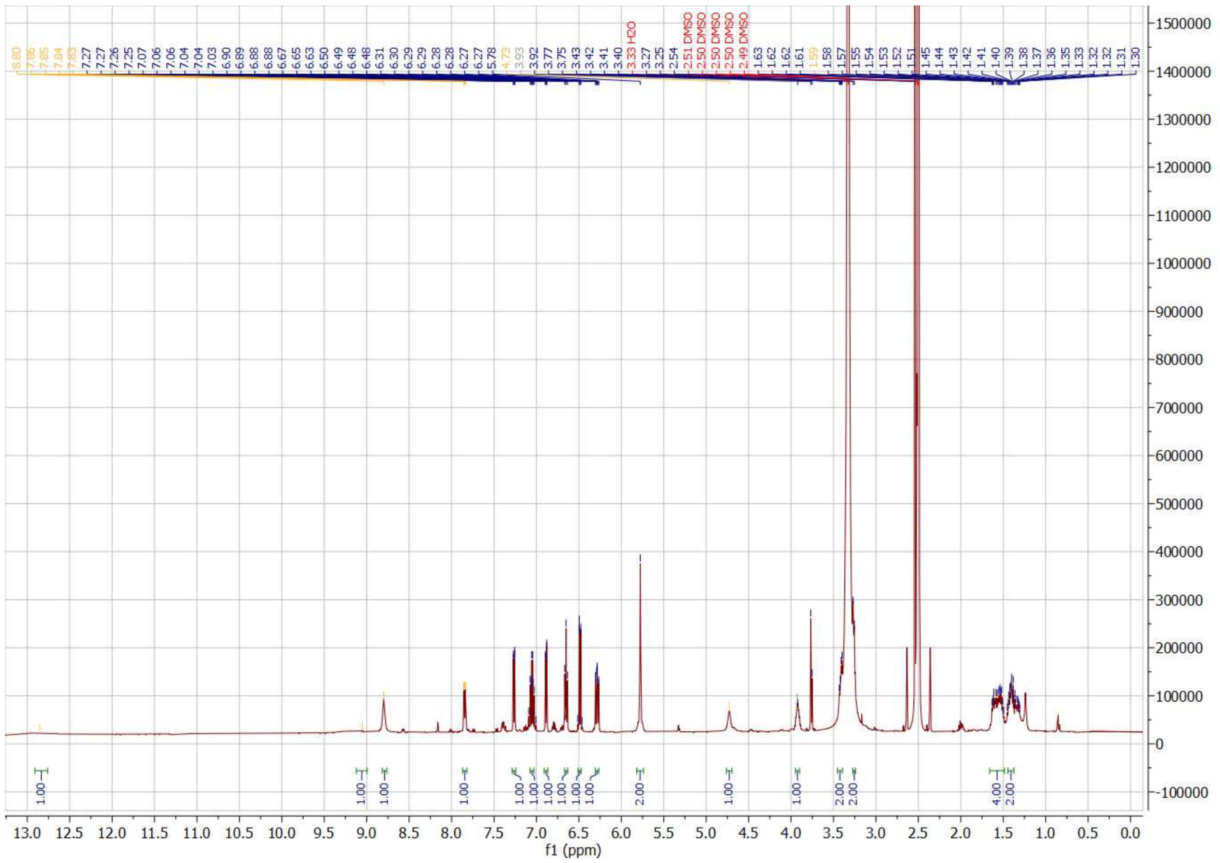

Figure S65. ^{1}H NMR spectrum of **9g** in DMSO-d_6.

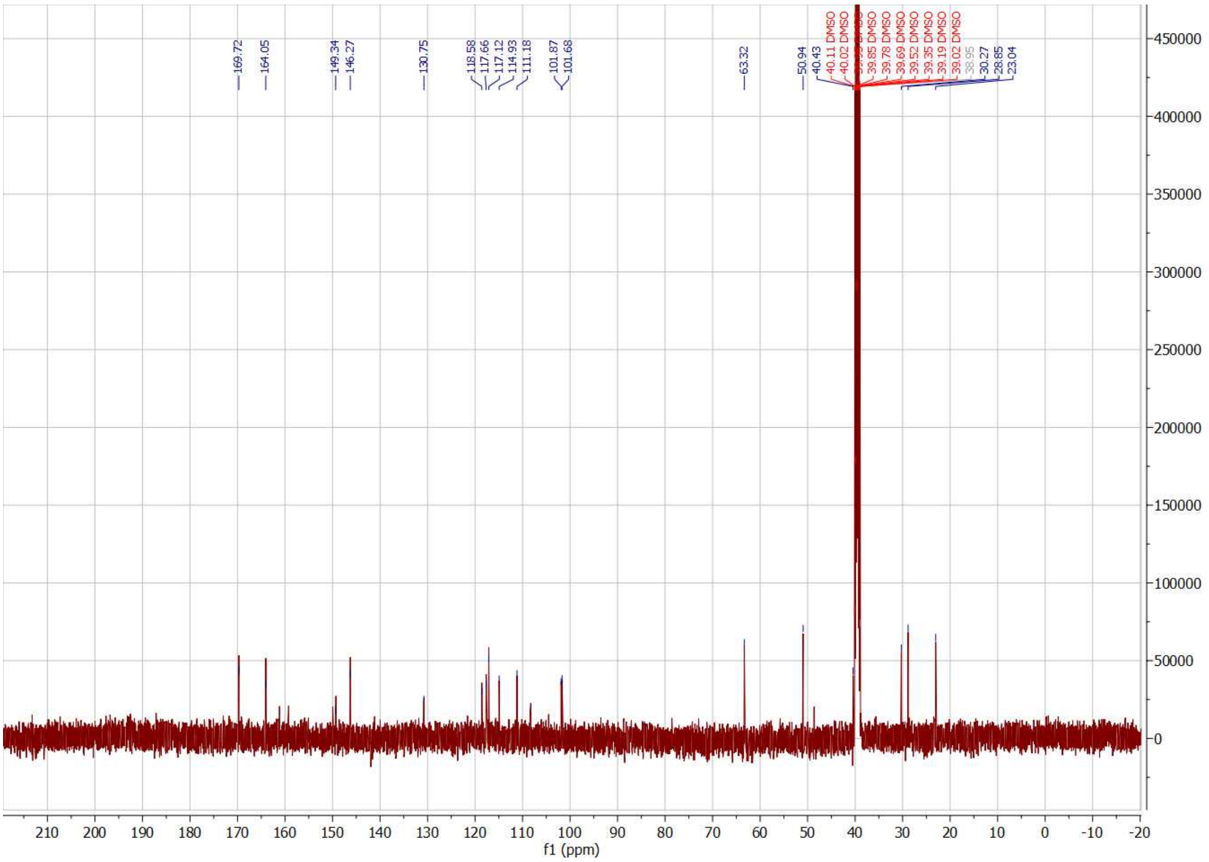

*Figure S66. ^{13}C NMR spectrum of **9g** in DMSO-d_6.*

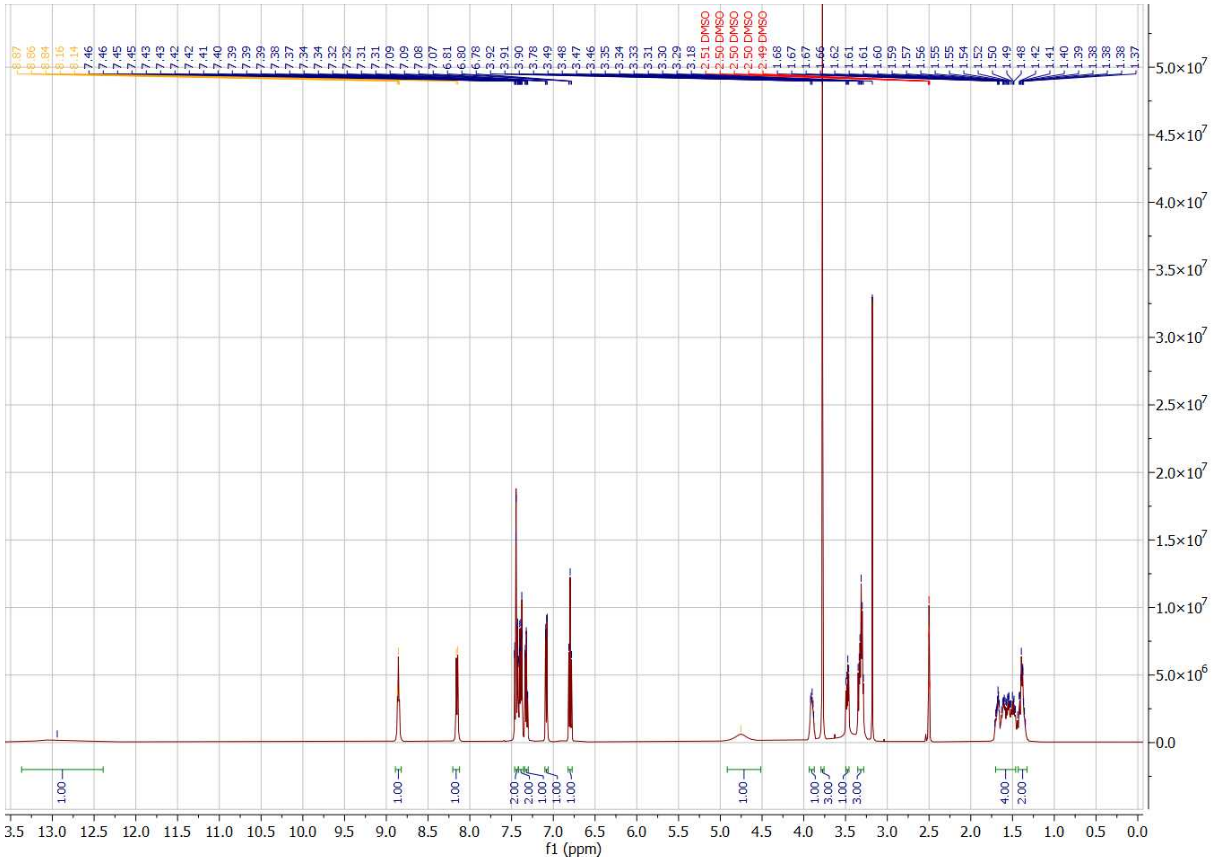

*Figure S67. ^{1}H NMR spectrum of **9h** in DMSO-d_6.*

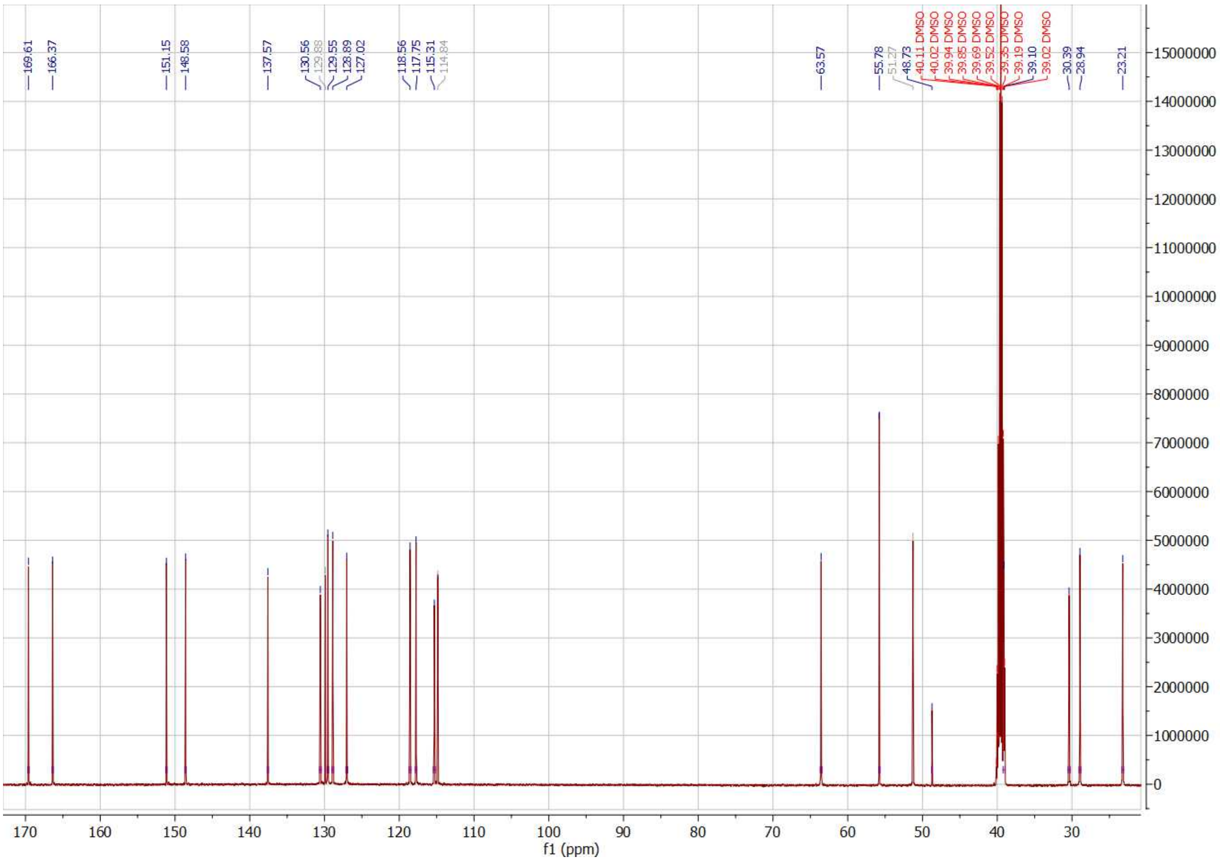

*Figure S68. ^{13}C NMR spectrum of **9h** in DMSO-d_6.*

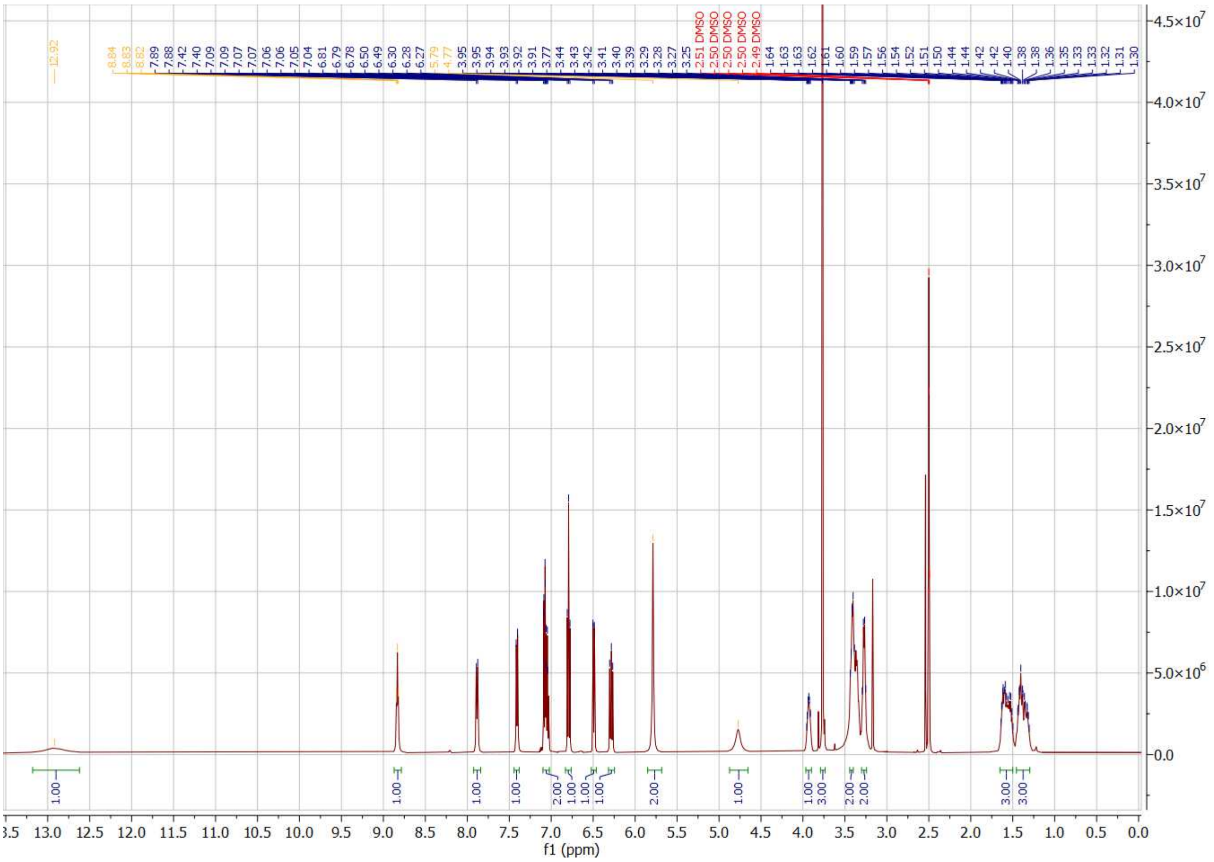

*Figure S69. ^{1}H NMR spectrum of **9i** in DMSO-d_6.*

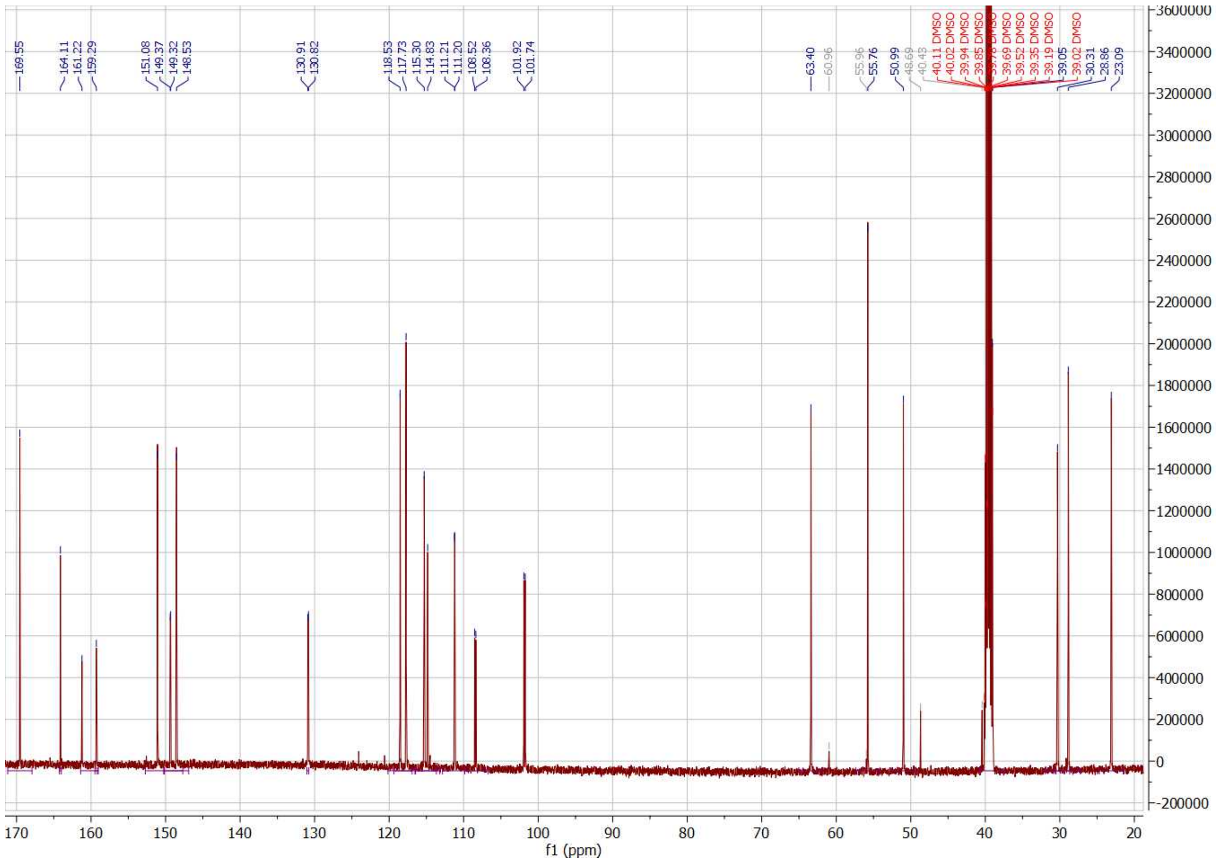

Figure S70. ^{13}C *NMR spectrum of* ***9i*** *in DMSO-* d_6.

2 *In-silico* analysis of the myxochelin N1–N3 biosynthesis

2.1 Metabolome database search of 1–3

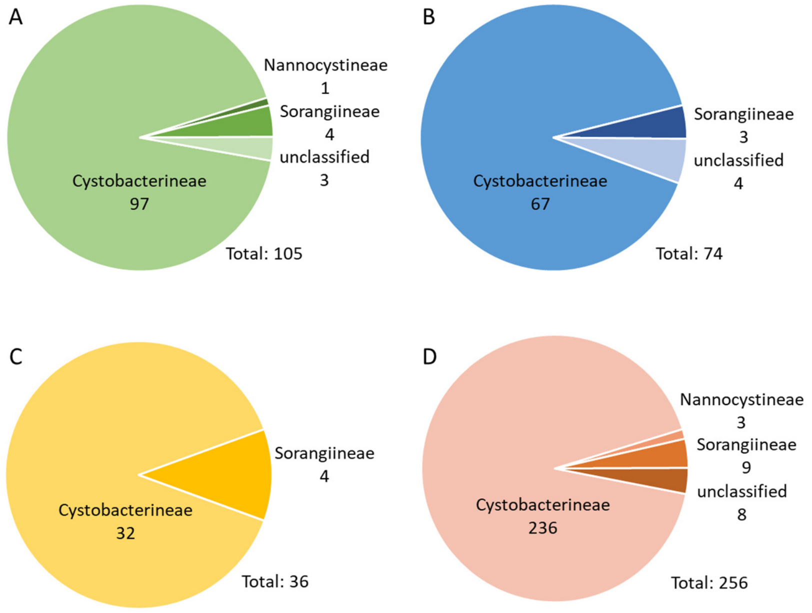

*Figure S71. Alternative producers of **1–3 (A–C)** and myxochelin A (**D**) from our in-house metabolome database sorted by suborder. Search parameters: exact mass deviation <5ppm, retention time deviation <0.3min, area threshold $3*10^4$. The decreasing number of hits from **1** to **3** is most likely due to the threshold of the peak finding algorithm.*

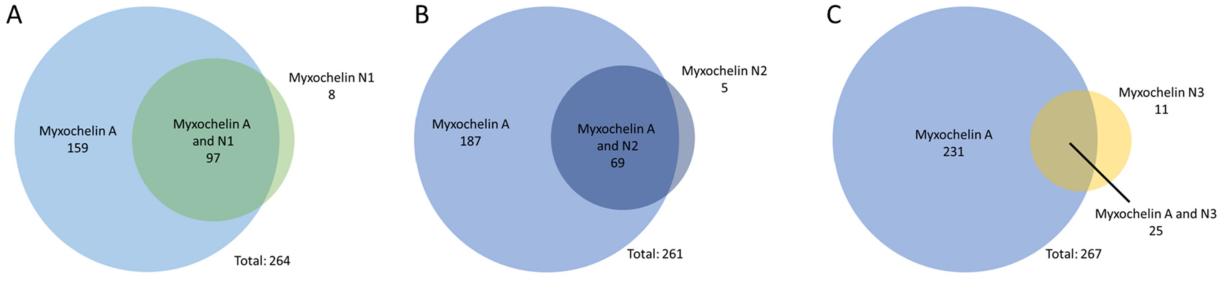

*Figure S72. Venn Diagrams of alternative producers of **1–3 (A–C)** and of Myxochelin A from our in-house metabolome database. Search parameters: exact mass deviation <5ppm, retention time deviation <0.3min, area threshold $3*10^4$.*

The strains that were found to produce **1**, **2**, and/or **3**, but not myxochelin A were manually screened for presence of **1**–**3** and myxochelin A. The 24 hits refer to 22 myxobacterial metabolomes in total. Out of these, 10 hits were found to produce myxochelin A and **1**, **2**, and/or **3** and 12 hits were found to produce neither **1–3** nor myxochelin A. No strain was observed to produce **1**, **2**, and/or **3** in absence of myxochelin A.

2.2 Analysis of the myxochelin BGC

Every coding sequence in the myxochelin biosynthesis gene cluster of MCy9049 was extracted translated and searched with the blastp algorithm against the NCBI RefSeq non-redundant protein sequence database.[1] MxcA (NADP-dependent alcohol oxidoreductase) and MxcB (removal of iron from siderophore complex) are found outside the myxochelin regulon.

Table S1. Tabulated blastP results for the CDS regions present in the MCy9049 myxochelin biosynthetic gene cluster

CDS Name	Length [AA]	Closest homologue [Organism of origin]	Identity [%] and alignment length [AA]	Proposed function	Accession Nr.
mxcC	280	2,3-dihydro-2,3-dihydroxybenzoate dehydrogenase [Corallococcus]	97.3 / 257		WP_120546994
mxcD	429	isochorismate synthase DhbC [Corallococcus sp. NCSPR001]	95.6 / 429		WP_206795025
mxcE	544	(2,3-dihydroxybenzoyl)adenylate synthase [Corallococcus interemptor]	98.3 / 544		WP_121769586
mxcF	301	isochorismatase family protein [Corallococcus sp. AB049A]	98.0 / 301	aryl carrier protein	WP_121723307
mxcG	1576	myxochelin non-ribosomal peptide synthetase MxcG [Corallococcus sp. AB049A]	92.6 / 913		WP_121723308
aroAA5	426	3-deoxy-7-phosphoheptulonate synthase [Corallococcus sp. AB049A]	99.3 / 426	DAHP synthase	WP_121723309
mxcH	856	TonB-dependent siderophore myxochelin receptor MxcH [Corallococcus interemptor]	97.0 / 856		WP_121769582
mxcI	422	hypothetical protein [Corallococcus interemptor]	92.9 / 422	unknown	WP_121769581
mxcK	405	myxochelin export MFS transporter MxcK [Corallococcus sp. AB049A]	96.3 / 405		WP_121725177
mxcL	427	myxochelin B biosynthesis transaminase MxcL [Corallococcus interemptor]	98.1 / 427		WP_121769587

Table S2. A-domain specificity codes for mxcE *homologs from NRPSpredictor2[2]. Prediction for all hits: dihydroxybenzoic acid or salicylic acid*

CDS Name	Organism of origin	8 angstrom signature code	Stachelhaus code
entE	*Escherichia coli* K12	SRSIYAMSSPGGALQVGGAQQVFGMAEGLVNYTR	AMPAQGVVNK
dhbE	*Bacillus subtilis subsp*. 168	SRSLYPLSSPGGALQVGGAQQVFGMAEGLVNYTR	PLPAQGVVNK
mxcE	*Corallococcus sp.* MCy9049	SASLFPLSSPGGALQVGGAQQVFGMAEGLVNYTR	PLPAQGVVNK
mxcE	*Archangium gephyra* DSM 2261	SASLFPLSSPGGALQVGGAQQVFGMAEGLVNYTR	PLPAQGVVNK
mxcE	*Archangium sp.* Cb G35	SGSLFPLSSPGGALQVGGAQQVFGMAEGLVNYTR	PLPAQGVVNK
mxcE	*Corallococcus coralloides* DSM 2259	SASLFPMSSPGGALQVGGAQQVFGMAEGLVNYTR	PMPAQGVVNK
mxcE	*Corallococcus sp.* H22C18031201	SASLFPLSSPGGALQVGGAQQVFGMAEGLVNYTR	PLPAQGVVNK
mxcE	*Cystobacter fuscus* DSM 2262	SGSLFPLSSPGGALQVGGAQQVFGMAEGLVNYTR	PLPAQGVVNK
mxcE	*Cystobacter violaceus* Cb vi76	SGSLFPLSSPGGALQVGGAQQVFGMAEGLVNYTR	PLPAQGVVNK
mxcE	*Haliangium ochraceum* DSM 14365	SASLFTLSSPGGALQVGGAQQVFGMAEGLVNYTR	TLPAQGVVNK
mxcE	*Hyalangium minutum* DSM 14724	SASLFPMSSPGGALQVGGAQQVYGMAEGLVNYTR	PMPAQGVVNK
mxcE	*Myxococcus fulvus* 124B02	SASLFPMSSPGGALQVGGAQQVFGMAEGLVNYTR	PMPAQGVVNK
mxcE	*Myxococcus fulvus* HW-1	SGSLFPLSSPGGAVQVGGAQQVFGMAEGLVNYTR	PLPAQGVVNK
mxcE	*Myxococcus stipitatus* DSM 14675	SASLFPMSSPGGALQVGGAQQVFGMAEGLVNYTR	PMPAQGVVNK
mxcE	*Myxococcus xanthus* DK 1622	SGSLFPLSSPGGAVQVGGAQQVFGMAEGLVNYTR	PLPAQGVVNK
mxcE	*Polyangium fumosum* DSM 14668	SASLFTLSSPGGSVQVGGSQQVFGMAEGLVNYTR	TLPSQGVVNK
mxcE	*Polyangium sorediatum* DSM 14670	SASLFTLSSPGGSVQVGGSQQVFGMAEGLVNYTR	TLPSQGVVNK
mxcE	*Polyangium sp.* SDU3-1	SASLFTLSSPGGAVQVGGAQQVFGMAEGLVNYTR	TLPAQGVVNK
mxcE	*Sandaracinus amylolyticus* DSM 53668	SASLFPLSSPGGALQVGGAQQVFGMAEGLVCYTR	PLPAQGVVCK
mxcE	*Stigmatella aurantiaca* DW4/3-1	SGSLFPLSSPGGALQVGGAQQVYGMAEGLVNYTR	PLPAQGVVNK
mxcE	*Stigmatella aurantiaca* S15	SGSLFPLSSPGGALQVGGAQQVYGMAEGLVNYTR	PLPAQGVVNK
mxcE	*Vitiosangium sp.* GDMCC 1.1324	SASLFPMSSPGGALQVGGAQQVFGMAEGLVNYTR	PMPAQGVVNK

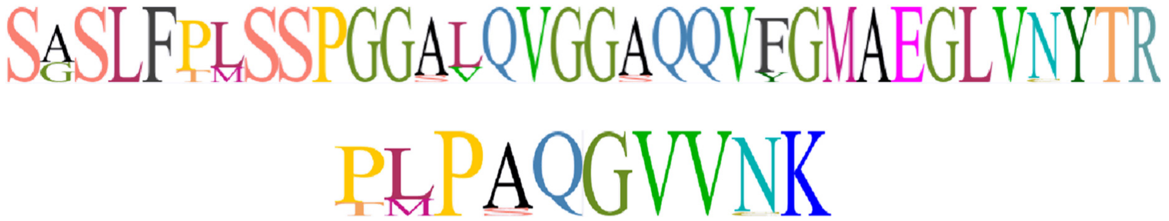

Figure S73. Consensus sequences for 8 angstrom signature code (upper) and Stachelhaus code (lower) of myxobacterial mxcE *translations.*

3 Assessment of biological activities

*Table S3. Minimum inhibitory concentrations (MICs) for the synthetic myxochelins against a panel of microorganisms and MICs of reference drugs in combination with **9a*** (128 µg/mL). -: not determined.

compound	MIC [µg/mL]				
	S. aureus str. Newman	*E. coli* BW25113	*E. coli* K12 Δ*tolC*	*C. albicans* DSM 1665	*M. hiemalis* DSM 2656
**9a**	> 128	> 128	> 128	> 128	> 128
**9b**	> 128	> 128	> 128	> 128	> 128
**9c**	> 128	> 128	> 128	> 128	> 128
**9d**	> 128	> 128	> 128	> 128	> 128
**9e**	> 128	> 128	> 128	> 128	> 128
**9f**	> 128	> 128	> 128	> 128	> 128
**9g**	> 128	> 128	> 128	> 128	> 128
**9h**	> 128	> 128	> 128	> 128	> 128
**9i**	> 128	> 128	> 128	> 128	> 128
Ciprofloxacin	0.125	-	-	-	-
Linezolid	2	-	-	-	-
Gentamicin	0.5	-	-	-	-
Daptomycin	1	-	-	-	-
Ciprofloxacin + **9a**	0.125	-	-	-	-
Linezolid + **9a**	2	-	-	-	-
Gentamicin + **9a**	0.5	-	-	-	-
Daptomycin + **9a**	1	-	-	-	-

4 References

1. O'Leary, N.A.; Wright, M.W.; Brister, J.R.; Ciufo, S.; Haddad, D.; McVeigh, R.; Rajput, B.; Robbertse, B.; Smith-White, B.; Ako-Adjei, D.; et al. Reference sequence (RefSeq) database at NCBI: current status, taxonomic expansion, and functional annotation. *Nucleic Acids Res.* **2016**, *44*, D733-45, doi:10.1093/nar/gkv1189.

2. Röttig, M.; Medema, M.H.; Blin, K.; Weber, T.; Rausch, C.; Kohlbacher, O. NRPSpredictor2—a web server for predicting NRPS adenylation domain specificity. *Nucleic Acids Res.* **2011**, *39*, W362-W367, doi:10.1093/nar/gkr323.
